# Comparative Genomics and Proteomic Analysis of Four Non-tuberculous *Mycobacterium* Species and *Mycobacterium tuberculosis* Complex: Occurrence of Shared Immunogenic Proteins

**DOI:** 10.3389/fmicb.2016.00795

**Published:** 2016-06-07

**Authors:** Nomakorinte Gcebe, Anita Michel, Nicolaas C. Gey van Pittius, Victor Rutten

**Affiliations:** ^1^Tuberculosis Laboratory, Agricultural Research Council - Onderstepoort Veterinary InstituteOnderstepoort, South Africa; ^2^Department of Veterinary Tropical Diseases, Faculty of Veterinary Science, University of PretoriaOnderstepoort, South Africa; ^3^Division of Molecular Biology and Human Genetics, Department of Biomedical Sciences, Faculty of Medicine and Health Sciences, Centre of Excellence for Biomedical Tuberculosis Research, Stellenbosch UniversityTygerberg, South Africa; ^4^Division of Immunology, Department of Infectious Diseases and Immunology, Faculty of Veterinary Medicine, Utrecht UniversityUtrecht, Netherlands

**Keywords:** non-tuberculous mycobacteria, Esx family, PE/PPE, *M. fortuitum*, *M. malmesburii* sp. nov., *M. komanii* sp. nov., *M. nonchromogenicum*

## Abstract

The Esx and PE/PPE families of proteins are among the most immunodominant mycobacterial antigens and have thus been the focus of research to develop vaccines and immunological tests for diagnosis of bovine and human tuberculosis, mainly caused by *Mycobacterium bovis* and *Mycobacterium tuberculosis*, respectively. In non-tuberculous mycobacteria (NTM), multiple copies of genes encoding homologous proteins have mainly been identified in pathogenic Mycobacterium species phylogenically related to *Mycobacterium tuberculosis* and *Mycobacterium bovis*. Only ancestral copies of these genes have been identified in nonpathogenic NTM species like *Mycobacterium smegmatis, Mycobacterium* sp. KMS, *Mycobacterium* sp. MCS, and *Mycobacterium* sp. JLS. In this study we elucidated the genomes of four nonpathogenic NTM species, *viz Mycobacterium komanii* sp. nov., *Mycobacterium malmesburii* sp. nov., *Mycobacterium nonchromogenicum*, and *Mycobacterium fortuitum* ATCC 6841. These genomes were investigated for genes encoding for the Esx and PE/PPE (situated in the *esx* cluster) family of proteins as well as adjacent genes situated in the ESX-1 to ESX-5 regions. To identify proteins actually expressed, comparative proteomic analyses of purified protein derivatives from three of the NTM as well as *Mycobacterium kansasii* ATCC 12478 and the commercially available purified protein derivatives from *Mycobacterium bovis* and *Mycobacterium avium* was performed. The genomic analysis revealed the occurrence in each of the four NTM, orthologs of the genes encoding for the Esx family, the PE and PPE family proteins in *M. bovis* and *M. tuberculosis*. The identification of genes of the ESX-1, ESX-3, and ESX-4 region including *esxA*, e*sxB, ppe68, pe5*, and *pe35* adds to earlier reports of these genes in nonpathogenic NTM like *M. smegmatis, Mycobacterium* sp. JLS and *Mycobacterium* KMS. This report is also the first to identify *esxN* gene situated within the ESX-5 locus in *M. nonchromogenicum*. Our proteomics analysis identified a total of 609 proteins in the six PPDs and 22 of these were identified as shared between PPD of *M.bovis* and one or more of the NTM PPDs. Previously characterized *M tuberculosis/M. bovis* homologous immunogenic proteins detected in one or more of the nonpathogenic NTM in this study included CFP-10 (detected in *M. malmesburii* sp. nov. PPD), GroES (detected in all NTM PPDs but *M. malmesburii* sp. nov.), DnaK (detected in all NTM PPDs), and GroEL (detected in all NTM PPDs). This study confirms reports that the ESX-1, ESX-3, and ESX-4 regions are ancestral regions and thus found in the genomes of most mycobacteria. Identification of NTM homologs of immunogenic proteins warrants further investigation of their ability to cause cross-reactive immune responses with MTBC antigens.

## Introduction

Purified protein derivatives (PPDs) also known as tuberculins have been used for more than 100 years as antigens in the diagnosis of human and bovine tuberculosis (TB), based on the delayed hypersensitivity (skin) reaction they induce. The first preparation of PPD was introduced by Robert Koch in 1890, where a boiled crude extract of Mycobacterium containing a mixture of proteins, both somatic and antigenic, referred to as “old tuberculin,” was prepared as a potential vaccine against tuberculosis in humans. Whilst the “old tuberculin” failed to live up to its initial claim of having curative properties, it formed the foundation for the modern PPD preparations (Burke, 1993). Tuberculin based tests (tuberculin skin test and the interferon gamma assay) have since been used as diagnostic tools for bovine tuberculosis. While these tests generally show satisfactory sensitivities, the major drawback to the tuberculin based assays is the reduced specificity. This is presumably associated with cross-reactive immune responses due to exposure of animals and humans to non-tuberculous mycobacteria (NTM) or environmental mycobacteria and the TB vaccine strain, Bacille Calmette et Guérin (BCG). These cross- reactive immune responses are most likely due to the presence of immunogenic proteins conserved across the *Mycobacterium* genus as major components of PPD derived from *M. bovis* (Schiller et al., 2010). PPD prepared from *Mycobacterium avium* is included as a control antigen representative of the background responses to environmental mycobacterial antigens in the single intradermal comparative cervical tuberculin test (SICCT) as well as the interferon gamma assays used in different countries in bovine tuberculosis (BTB) control strategies (Vordermeier et al., 2006). As an alternative approach to mitigate the frequency of false positive responses to PPD-B induced by environmental mycobacterial antigens a PPD derived from *Mycobacterium fortuitum* (PPD-F) used in the modified BOVIGAM assay, has shown to increase the test specificity in South Africa (Michel et al., 2011).

Interference of NTM and BCG in immune responsiveness against bacteria of the *Mycobacterium tuberculosis* complex (MTBC) has triggered investigation of “specific” or defined antigens which are uniquely present in pathogenic mycobacteria, and absent in most NTM as well as *M. bovis* BCG as markers for the diagnosis of bovine and human tuberculosis (Schiller et al., 2010). Comparative genomics studies have led to the identification and characterization of certain genetic regions in the genomes of mycobacteria that are present in *Mycobacterium tuberculosis* and *Mycobacterium bovis* but absent in *M. bovis* BCG and most NTM. The Region of Difference 1 (RD1), a major deleted region in BCG first described by Mahairas et al. (1996) seems to have been the early event in the attenuation of the present vaccine strain, hence proteins within this region are studied extensively in order to try to understand the virulence of pathogenic MTBC. The two most predominant proteins encoded in the RD1 region, namely the 6 kDa early secreted antigenic target (ESAT-6/EsxA) and the 10 kDa culture filtrate protein (CFP-10/EsxB) have been investigated widely for inclusion into candidate vaccines and used as antigens in the diagnosis of both TB and BTB (Pym et al., 2003; Vordermeier et al., 2006; Ganguly et al., 2008). Functional studies have revealed that RD1 encodes a specialized system for secretion in mycobacteria, involving genes situated both inside the RD1 locus and its flanking regions, known as the ESAT-6 secretion system (Brodin et al., 2004). The ESAT-6 and CFP-10 proteins belong to the Esx family that consists of 23 small secreted proteins encoded by genes *esxA-esxW* and arranged in 11 gene pairs (Bitter et al., 2009). Five of the 11 genomic pairs are contained in five loci: ESX-1 to ESX-5 encoding components of the type VII secretion system (Uplekar et al., 2011). Four of the five loci, except ESX-4, also harbor 11 proteins of the Proline Glutamate (PE), or Proline Proline Glutamate (PPE) family, several of which have been implicated in immune evasion (Akhter et al., 2012; Mustafa, 2014). The PE and PPE and Esx proteins whose genes are adjacent to each other in the genome of *Mycobacterium tuberculosis* interact to form pairs which appear to be essential for their secretion (Renshaw et al., 2005). The secretory proteins have been the focus of research aimed at development of TB vaccines and immunodiagnostic assays, because they are thought to have a potential to induce protective immunity and immune responses of diagnostic value (Ize and Palmer, 2006; Ganguly et al., 2008; Marongui et al., 2013; Mustafa, 2014). Studies have shown however, that some genes encoding for proteins of the Esx family, as well as PE/PPE do occur in pathogenic NTM like *Mycobacterium kansasii Mycobacterium marinum, Mycobacterium szulgai, Mycobacterium gordonae, Mycobacterium gastri, Mycobacterium ulcerans, Mycobacterium* sp. JDM601, and *Mycobacterium riyadhense*, as well as in *Mycobacterium leprae* (Gey van Pittius et al., 2001, 2006; Geluk et al., 2002; 2004; Newton-Foot et al., 2016) while genes encoding for PE/PPE family of proteins were also identified in *Mycobacterium abscessus* and *Mycobacterium avium* (Sorensen et al., 1995; Vordermeier et al., 2007; van Ingen et al., 2009; Mustafa, 2014). The immunogenicity and cross-reactivity against MTBC antigens of the ESAT-6 and CFP 10 proteins of pathogenic NTM was as well-demonstrated in *M. kansasii* (Waters et al., 2010). Therefore use of these antigens in BTB diagnosis may be hampered by the exposure of humans and animals to pathogenic NTM leading to false positive BTB immunological test results.

Comparative genomic studies of rapidly growing mycobacteria (RGM) have shown that, contrary to the genomes of pathogenic MTBC and other pathogenic slow-growing NTM like *M. kansasii* and *M. marinum* which contain the five ESX regions (ESX-1 to ESX-5), the nonpathogenic RGM such as *Mycobacterium flavescens, M*. sp. JLS, *M*. sp. MCS, *M*. sp. KMS, *Mycobacterium phlei, Mycobacterium thermoresistible, Mycobacterium gilvum* PYR-GCK, *Mycobacterium vaccae, Mycobacterium vanbaalenii*, and *M. smegmatis* genomes only harbor ESX-1, ESX-3, and ESX-4 (Gey van Pittius et al., 2006; Newton-Foot et al., 2016). It was therefore hypothesized that the three regions (ESX-1, ESX-3, and ESX-4) are genetic factors of all mycobacteria and ESX-2 and ESX-5 are probably absent in all RGM (Gey van Pittius et al., 2006).

To assess the presence of homologous proteins in NTM potentially able to elicit cross-reactive immune responses against MTBC antigens we elucidated the genomes of four NTM species, *viz Mycobacterium nonchromogenicum, Mycobacterium fortuitum* ATCC 6841, *Mycobacterium malmesburii* sp. nov., and *Mycobacterium komanii* sp. nov. and compared these with the genomes of *Mycobacterium tuberculosis* H37Rv and *Mycobacterium bovis* AF2122/97 to identify orthologous genes situated in the ESX-1 to ESX-5. In addition, the proteomes of PPDs derived from the NTM *viz M. nonchromogenicum* (PPD-N), *M. fortuitum* ATCC 6841 (PPD-F), *M. malmesburii* sp. nov (PPD-M), and *M. kansasii* ATCC 12478 (PPD-K) were defined to identify the proteins actually produced and compared to those of the commercially available PPDs derived from *M. avium* and *M. bovis*. This is the first report of the occurrence of selected genes encoding proteins that were previously shown to be immunogenic in *M. bovis*/*M. tuberculosis* in the four NTM genomes as well as the expression of proteins they code for.

## Materials and methods

### Bacterial cultures

*M. malmesburii* sp. nov. and *M. komanii* sp. nov. were isolated from cattle nasal swabs during an NTM survey in South Africa (Gcebe et al., 2013). These two species were previously identified and characterized as novel NTM (Gcebe et al., unpublished). The *M. nonchromogenicum* strain was isolated from soil during an NTM survey (Gcebe et al., 2013). *M. fortuitum* ATCC 6841 and *M. kansasii* ATCC 12478 were obtained from the Agricultural Research Council-Onderstepoort Veterinary Institute, South Africa. The identity of all the strains used was confirmed by PCR-sequencing assays targeting the 16S rDNA (Gcebe et al., 2013), *hsp65* (Telenti et al., 1993), *rpoB* (Adékambi et al., 2006), and *sod*A (Adékambi and Drancourt, 2004). For PPD production, liquid cultures were prepared in Middlebrook 7H9 medium (Becton Dickinson, USA) supplemented with 0.1% OADC and glycerol, incubated under continued shaking at 200 *g* at 37°C (optimum growth temperature) for 4 weeks for the rapid growing mycobacteria (*M. fortuitum* and *M. malmesburii* sp. nov.) and 6 weeks for the slow growing NTM (*M. kansasii* and *M. nonchromogenicum*) or until turbid growth was observed. The liquid cultures were screened for contaminants before PPDs were prepared by spread plating each culture on two nutrient agar plates. The plates were incubated at 25 and 37°C respectively, and evaluated after 2 and 5 days for fungal or any bacterial growth not typical of mycobacteria.

### Whole genome sequencing, assembly, and annotation

DNA was extracted from bacterial cultures (*M. fortuitum, M. nonchromogenicum, M. komanii* sp. nov*.*, and *M. malmesburii* sp. nov.) in Löwenstein -Jensen medium (Becton Dickinson, USA) using the Qiagen nucleic acid extraction kit (Whitehead Scientific, South Africa). Genomic DNA paired-end libraries were generated using the Nextera DNA sample preparation kit (Illumina) and indexed using the Nextera index kit (Illumina). Sequencing was performed as paired-end reads (2 × 250 bp) employing the Illumina Miseq system using the Miseq reagent kit v2 at the Agricultural Research Council, as per manufacturer's instructions. *De novo* assembly of the sequencing reads was performed using SPAdes, which mainly involves construction of DNA sequences of contigs and the mapping of reads to contigs (Bankevich et al., 2012). Each assembly was evaluated using QUAST (Gurevich et al., 2013). The assembled genome sequences were annotated using Prokka annotation pipeline (Seemann, 2014). This involved predicting tRNA, rRNA, and mRNA genes in the sequences and assigning putative gene products to the protein-coding genes (CDSs) based on their similarity to sequences in a database of curated *Mycobacterium* genes. To gauge the similarity of the NTM genomes to those of *M. bovis* AF2122/97 (NC 002945.3) and *M. tuberculosis* H37Rv (NC 000962.2), alignment of each of the processed sets of reads to these reference genomes was performed using Burrow's Wheeler Aligner (BWA) (Li and Durbin, 2009). BLAST Ring Image Generator (Alikhan et al., 2011) was used to visualize the pairwise BLAST results of CDS features in *M. bovis* and each NTM annotation against that of *M. tuberculosis* and against the respective closest NTM relative.

### Investigating the closest sequence relatives of the NTM strains

Basic local Alignment Search tool (BLAST) search of the largest contigs from each NTM assembly was performed against the NCBI Genbank database (http://www.ncbi.nlm.nih.gov/genbank/) to identify the closest known sequence relative of each NTM species. BLAST searches were also performed for the annotation CDS features for each NTM species against the reference genomes for their respective best hits and visualized using BLAST Ring Image Generator (BRIG) (Alikhan et al., 2011).

### Identification of predicted immunogenic proteins in NTM genomic annotations

BLAST searches were conducted to quantify the similarity of the annotated amino acid sequences in the NTM assemblies (*M. fortuitum, M. malmesburii* sp. nov*., M. komanii* sp. nov., and *M. nonchromogenicum*) to immunogenic proteins in *M. bovis, M. tuberculosis*, and *M. smegmatis*. Esx family proteins, PE and PPE proteins situated within the ESX-1 to ESX-5 clusters were chosen for this investigation (see Table 1 for the complete list of targeted proteins). BLASTP searches against the NCBI Genbank database were also conducted for the respective NTM protein sequences to quantify similarity to other sequences in the database. Multiple amino acid sequence alignments of the individual proteins: (Esx, PE, PPE situated in ESX-1 and ESX-3) and from different mycobacteria were performed using Clustalw (Thompson et al., 1994) from MEGA (version 6), to assess sequence similarities of the *M. bovis*/*M. tuberculosis* and the NTM orthologs at immunogenic epitope level. Amino acid and nucleotide sequences from *M. bovis and M. tuberculosis* were retrieved from Genolist (http://genolist.pasteur.fr) and those of *M. smegmatis* were retrieved from Smegmalist (http://mycobrowser.epfl.ch/smegmalist.html) databases. NTM protein orthologs that showed amino acid sequence similarities of < 50% to the respective *M. bovis* antigen were not included in the alignments. To confirm orthology of the *esx* gene clusters present in NTM in this study (ESX-1, ESX-3, and ESX-4 as well as ESX-2 of *M. nonchromogenicum*) to the MBTC (http://genolist.pasteur.fr) and *M. smegmatis* MC^2^155 (http://mycobrowser.epfl.ch/smegmalist.html) ESAT-6 regions the four NTM genomes were also assessed for the genes adjacent to the *esx* and *pe/ppe*. The genomic organization of the ESX-1, ESX-3, and ESX-4 of the NTM including ESX-2 of *M. nonchromogenicum* was also evaluated.

**Table 1:** **List of target genes within ESX-1 to ESX-5 loci of ***M. bovis*** and ***M. tuberculosis*****.

**Name/Gene ID**	**Species**	**RefSeq/Genbank**	**Locus Tag**	**Protein product**
esxA	M.tb	WP_000339993.1	Rv3875	6 kDa early secretory antigenic target (ESAT-6)
	*M. bovis*		Mb3905	6 kDa early secretory antigenic target (ESAT-6)
esxB	M. tb	WP_003399940.1	Rv3874	10 kDa culture filtrate antigen LHP (CFP10)
	*M. bovis*		Mb3904	10 kDa culture filtrate antigen LHP (CFP10)
esxC	M.tb	WP_003899750	Rv3890c	ESAT-6 like protein EsxC (ESAT-6 like protein 11)
	*M. bovis*		Mb3919c	Putative esat-6 like protein 11
esxD	M.tb	WP_003400035.1	Rv3891C	Possible esat-6 like protein EsxD
	*M. bovis*		Mb3920C	Conserved hypothetical protein
esxG	M.tb	WP_003401503.1	Rv0287	ESAT-6 like protein EsxG
	*M. bovis*		Mb0295	Hypothetical protein
esxH	M. tb	WP_003401514.1	Rv0288	ESAT-6 like protein EsxH
	*M. bovis*		Mb0296	low molecular weight protein antigen 7 CFP7 (TB10.4)
esxT	M. tb	WP_003900059.1	Rv3444C	ESAT-6 like protein EsxT
	*M. bovis*		Mb3474C	Hypothetical protein
esxU	M. tb	WP_003900060.1	Rv3445C	ESAT-6 like protein EsxU
	*M. bovis*		Mb3475C	Hypothetical protein
esxN	M. tb	WP_003408840	Rv1793	ESAT-6 like protein EsxN
	*M. bovis*		Mb1821	ESAT-6 like protein 5
pe35	M. tb	WP_003912361.1	Rv3872	PE family protein
	*M. bovis*		Mb3902	PE family- like protein
ppe68	M. tb	WP_003399879.1	Rv3873	PPE family protein
	*M. bovis*		Mb3903	PPE family protein
pe5	M. tb	WP_003401485.1	Rv0285	PE family protein
	*M. bovis*	NP_853957.1	Mb0293	PE family protein
ppe4	M. tb	WP_003401488.1	Rv0286	PPE family protein
	*M. bovis*		Mb0293	PPE family protein
ppe25	M. tb	WP_003901254.1	Rv1787	PPE family protein
	*M. bovis*		Mb1815	PPE family protein
pe18	M.tb	WP_003408976.1	Rv1788	PE family protein
	*M. bovis*		Mb1816	PE family protein
ppe26	M. tb	WP_003408979.1	Rv1789	PPE family protein
	*M. bovis*		Mb1817	PPE family protein
ppe27	M. tb	WP_003408805.1	Rv1790	PPE family protein PPE27
	*M. bovis*		Mb1818	PPE family protein
pe19	M. tb	WP_003408807.1	Rv1791	PE family protein
	*M. bovis*		Mb1819	PE family protein

### PCR verification of the presence of genes in ESX-1, ESX-3 as well as *esxN* in NTM

We set out experiments to confirm the presence of ESX-1 in the four NTM genomes by PCR and sequencing of the two most investigated genes in this region: *esxA* and *esxB*. The presence of ESX-3 in the four newly sequenced NTM was verified by amplification of *esxH*. Since it was previously shown by hybridization experiments that ESX-5 might be absent in *Mycobacterium nonchromogenicum* we verified the presence of *esxN* ortholog (situated in ESX-5 locus) in *M. nonchromogenicum* by PCR and sequencing.

The primers used for amplification of *esxA* were MSMEG_0066 –F (5′-AATTTCGCCGGT ATCGAGGG- 3′) located at position 19–38 bp of the MSMEG_0066 gene (an *M. smegmatis* MC^2^155 *esxA* ortholog), and MSMEG_0066-R (5′ CAG GCAAACATTCCCGTGAC 3′) located at position 278–268 bp of the *esxA* ortholog of *M. smegmatis* MC^2^155 (MSMEG_0066).

The primers for amplification of *esxB* were MSMEG_0065-F (5′ GCGAATTTCGAGCGCATCTC 3′) situated at position 46–65 bp, and MSMEG_0065-R (5′ GATGTTCATCGACGACGCAAG 3′) situated at position 300–280 bp.

Primers used for *esxH* were esxH-13 (5′- ARGTACAACTACCCGGCGATG-3′-) situated at position 13–33 bp of MSMEG_0621 gene (an *M. smegmatis* ortholog of *esxH*), and esxH-245 (5′-GCCATGGTGTTCTGCTCGT-3′) situated at position 245–226 bp of MSMEG_0621 gene.

Finally, primers used for amplification of *esxN* were esxN-F (5-′ GACGATTAATTACCAGTTCG-3′) situated at position 3–20 bp of the gene in *M. tuberculosis*, and esxN-R (5′-GGTTTGCGCCATGTTGT-3′) situated at position 255–239 bp of the gene.

#### PCR

Boiled culture suspensions were prepared from colonies as template for DNA amplification. The following PCR conditions were used for amplification of each of the three gene fragments: A 50 μl PCR mixture was prepared, containing 28.5 μl de-ionized water, 3 μl MgCl_2_(25 mM), 1 μl dNTP mix (10 mM), 4.75 μl of 10x PCR buffer (160 mM) (Tris -HCl, MgCl_2,_ Tween 20,(NH_4_)_2,_SO_4_), 0.75 μl Taq DNA Polymerase (5 U/μl) (Supertherm ™), 1 μl of each forward and reverse primers (50 pmol), and 10 μl of DNA template. The PCR cycling parameters were as follows: Fourty five cycles of denaturation at 94°C for 1 min, annealing at 60°C (55°C for *esxN*) for 1 min, and elongation at 72°C for 1 min and final extension at 72°C for 10 min. The PCR products were ran on a 1.5% agarose gel and visualized under ultra violet light (UV).

#### Sequencing and sequence analysis

Sequencing of the PCR products was done at Inqaba Biotechnologies (Pretoria, South Africa). Sequencing was performed in both directions using the forward and reverse primer sequences that were initially used for amplification. Sequences from both strands were edited manually and pairwise alignments undertaken using the BioEdit Sequence alignment editor (version 7.1.9) and Molecular Evolutionary Genetics Analysis (MEGA) platform (http://www.megasoftware.net) (version 6) (Tamura et al., 2013). The resulting consensus sequences were analyzed on the NCBI platform for gene sequence identity/similarity using BLAST (http://blast.ncbi.nlm.nih.gov/Blast.cgi).

### PPD production

PPDs were prepared from *M. nonchromogenicum* (PPD- N)*, M. malmesburii* sp. nov. (PPD-M), *M. fortuitum* ATCC 6841 (PPD-F), and *M. kansasii* ATCC 12478 (PPD-K). Three preparations of each PPD [one at a different time interval (first preparation) and two done at the same time (second and third preparations)] were done following a modified protocol by Landi (1963). Briefly, liquid cultures were inactivated by steaming at 121°C for 20 min. and filter- sterilized using the Whatman 40 filter paper and a vacuum pump. Each filtrate was then precipitated by adding 40% trichloroacetic acid (TCA) to a final concentration of 4% v/v) and left for at least 12 h at 4–8°C. Afterwards the precipitated filtrates were mixed manually by shaking and centrifuged at room temperature for 20 min at 3900 g. The supernatants were discarded and the pellets washed twice by suspending them in 1% TCA and careful mixing, followed by centrifugation at 3900 g for 20 min at room temperature. The supernatants were discarded and the pellets suspended in 10% NaCl, then centrifuged for 20 min at 3900 g. After discarding the supernatant, the pellet was harvested by turning the tube upside down, on a piece of sterile filter paper and allowed to dry, weighed, diluted with 0.005% PPD buffer (0.005% Tween 80 in PBS: PH = 7.38) and stored at 4–8°C until peptide digestion. The purified protein derivatives from *M. bovis* (PPD-B) and *M. avium* (PPD-A) were obtained from Prionics at the Netherlands.

### In-solution digest of PPDs with trypsin (IAA)

Trypsin digestion of each PPD preparation was done at the Centre for Proteomics and Genomics Research (CPGR, South Africa). All reagents used were analytical grade or equivalent. Twenty microgram of protein was dissolved in 10 μl of 50 mM triethylammonium bicarbonate (TEAB; Sigma). Protein was reduced by adding 1 μl of 100 mM tris (2-carboxyethyl) phosphine (Sigma) prepared in 50 mM TEAB, and incubated at 60°C for 1 h. Samples were cooled to room temperature and the protein was alkylated by adding 1 μl of 200 mM iodoacetamide (Sigma) prepared in 50 mM TEAB. Samples were incubated at room temperature in the dark for 30 min. The sample volume was adjusted to 50 μl with 50 mM TEAB and then 5 μl of 1 μg/μl trypsin (Promega), prepared in MilliPore water, was added and digestion was allowed to take place at 37°C for 18 h, followed by vacuum centrifugation.

### Mass spectrometry

#### LC–MS/MS analysis

LC-MS/MS analysis was conducted in triplicate for NTM PPDs from the second and the third preparations and once for the first preparation and the commercial PPDs (PPD from *M. bovis* and from *M. avium*) using a Q-Exactive quadrupole-Orbitrap mass spectrometer (Thermo Fisher Scientific, USA) coupled with a Dionex Ultimate 3000 nano-HPLC system at CPGR. The mobile phases consisted of solvent A (0.1% formic acid in water) and solvent B (80% ACN, 10% water, and 0.1% formic acid). The peptides (as an estimate 500 ng for each sample) were re-suspended in sample loading buffer (95% water, 5% Acetonitrile, 0.05% TFA) and loaded on a C18 trap column (100 μm × 20 mm × 5 μm). Chromatographic separation was performed with a C18 column (75 μm × 1 50 mm × 3 μm). The gradient was delivered at 300 nl/min and consisted of a linear gradient of mobile phase B initiating from solvent B, 6–60% over 156 min. The mass spectrometer was operated in positive ion mode with a capillary temperature of 250°C. The applied electrospray voltage was 1.95 kV. Details of data acquisition are listed in Table 2.

**Table 2:** **Data acquisition**.

**FULL SCAN**	
Resolution	70,000 (@ *m/z* 200)
AGC target value	3e6
Scan range	320–2000 m/z
Maximal injection time (ms)	100
**DATA-DEPENDENT MS/MS**	
Resolution	17,500 (@ *m/z* 200)
AGC target value	2e5
Maximal injection time (ms)	50
Isolation window width (Da)	3
NCE (%)	27
**DATA-DEPENDENT SETTINGS**	
Underfill ratio (%)	1
Charge exclusion	Charge states 1,6-8,>8
Peptide match	preferred
Exclusion isotopes	on
Dynamic exclusion (s)	60

### LC-MS/MS data analysis

#### Database searching

All MS/MS samples were analyzed using Mascot (Matrix Science, London, UK; version 2.4.1) and X! Tandem (The GPM, thegpm.org; version CYCLONE (2010.12.01.1). Mascot was set up to search the *Mycobacterium* database (derived from UniProtKB, 843597 entries) assuming the digestion enzyme trypsin. X! Tandem was set up to search a subset of the *Mycobacterium* database including also assuming trypsin. Mascot and X! Tandem were searched with a fragment ion mass tolerance of 0.020 Da and a parent ion tolerance of 10.0 PPM. Carbamidomethyl of cysteine were specified in Mascot and X! Tandem as fixed modifications. Gln->pyro-Glu of the n-terminus, deamidation of asparagine and glutamine, oxidation of methionine were specified in Mascot and X! Tandem variable modifications.

#### Criteria for protein identification

Scaffold (version Scaffold_4.3.4, Proteome Software Inc., Portland, OR) was used to validate MS/MS based peptide and protein identifications. Peptide identifications were accepted if they could be established at greater than 9.0% probability to achieve an FDR less than 0.1% by the Scaffold Local FDR algorithm (Keller et al., 2002). Protein identifications were accepted if they could be established at >100.0% probability to achieve an FDR less than 1.0% and contained at least four identified peptides (Keller et al., 2002). Protein probabilities were assigned by the Protein Prophet algorithm (Nesvizhskii et al., 2003). Proteins that contained similar peptides and could not be differentiated based on MS/MS analysis alone were grouped to satisfy the principles of parsimony.

## Results

### Alignment of the four NTM sequence reads with the genomes of *M. bovis* and *M. tuberculosis*

Alignment of the NTM reads (DNA sequences) and the reference genomes [*M. bovis* (NC 002945.3) and *M. tuberculosis* (NC 000962.3)] showed very little similarity between the NTM genomes and each of the reference strains. *M. komanii* sp. nov. genome seems to be the closest of the NTM to the reference genomes (18.61 and 18.69% of the sequence reads maps to *M. bovis* and *M. tuberculosis* genomes, respectively), followed by *M. nonchromogenicum* (15.71 and 15.8%), and *M. fortuitum* (10.58 and 10.63%). *M. malmesburii* sp. nov. showed the least similarity of its genome to that of the reference strains (8.83 and 8.89%). The GC contents of the NTM genomes were 66.19% for *M. fortuitum*, 66.25% for *M. nonchromogenicum*, 67.4% for *M. malmesburii* sp. nov., and 67.33% for *M. komanii* sp. nov., which is as high as the GC contents of *M. tuberculosis* and *M. bovis* (both have GC contents of 66.5%).

### *De novo* assembly of the NTM DNA sequence reads

The result for the assembly of the DNA sequence reads is summarized in Table 3. The number of contigs, the length of the largest contig as well as the N50 (the length for which the set of contigs of that length or longer contains at least half the assembly bases) and L50 (the number of contigs of that length for each assembly is presented). The overwhelming majority of the sequenced bases fall within the few largest contigs, but each assembly also produced a large number of very short contigs. The assembly for *M. komanii* sp. nov. produced 63 contigs, with its largest contig 292, 570 bases in size. While the assembly for *M. nonchromogenicum* produced the largest contig of all (425, 774 bases), it also produced the highest number of contigs (642 bases). The *M. fortuitum* and *M. malmesburii* sp. nov assemblies provided high total numbers of contigs (179 and 255 respectively), their largest contigs were 195, 004 and 135, 683 bases respectively.

**Table 3:** **Summary of the ***de novo*** assembly results**.

**NTM Species**	**Strain ID**	**number of contigs (*n*)**	**Largest contig (*bp*)**	**Total Length (*bp*)**	**L50**	**N50**
*M. fortuitum*	ATCC 6841	179	195 004	6 275 573	27	82 193
*M. nonchromogenicum*	NCK 8460	642	425 774	6 623 407	17	131 682
*M. malmesburii* sp. nov.	WCM 7299	255	135 683	5 426 606	42	42 651
*M. komanii* sp. nov.	GPK 1020	63	298 570	5 370 343	12	162 386

### Annotation of the assembled genome

The summary of the annotation is provided in Table 4. The numbers of protein coding genes, sequences encoding tRNA and rRNA and signal peptides identified in each of the four NTM genomes are indicated. The annotated assemblies were deposited into the European Nucleotide Archive (ENA) database (http://www.ebi.ac.uk/ena). The sequence identifiers i.e., locus tag and the accession numbers are BN1849 and CVRX01000001-CVRX01000338, respectively for *M. fortuitum*; BN1848 and CVTC01000001-CVTC01002268, respectively for *M. nonchromogenicum*; BN1837 and CVTB01000001-CVTB01000324, respectively for *M. malmesburii* sp. nov.; and finally BN1838 and CVTA01000001-CVTA0100065, respectively for *M. komanii* sp. nov.

**Table 4:** **Annotation of the assembled genome, showing number of CDS, tRNA, rRNA, and signal peptides**.

**NTM species**	**Strain ID**	**CDS**	**tRNA**	**rRNA**	**Signal peptides**
*M. fortuitum*	ATCC 6841	6097	66	3	499
*M. nonchromogenicum*	NCK 8460	6903	70	3	544
*M. malmesburii* sp. nov.	WCM 7299	5298	54	2	401
*M. komanii* sp. nov.	GPK 1020	5141	53	2	408

*NTM, non-tuberculous mycobacterium, CDS, coding sequences*.

### Closest sequence relatives of the four NTM genomes (*M. malmesburii* sp. nov., *M. komanii* sp. nov., *M. nonchromogenicum*, and *M. fortuitum* ATCC 6841)

Table 5 represents the BLAST results for each NTM's largest contig. *Mycobacterium fortuitum* and to a lesser extent *M. nonchromogenicum* have the closest resemblance to *M. smegmatis* MC^2^ 155 (accession number CP001663.1) with *M. malmesburii* sp. nov. displaying similarity to *M. rhodesiae* (CP003169.1). *M. komanii* sp. nov. seems to be the most novel organism scoring its highest BLAST score with *Mycobacterium*. sp. JLS (CP000580.1).

**Table 5:** **BLAST results using the largest contig of each NTM against Genbank nucleotide database (***E*** < 1 × 10^**6**^)**.

**NTM species**	**Species hit**	**BLAST score**	**Query coverage (%)**	**Identity (%)**
*M. fortuitum*	*M. smegmatis* MC^2^ 155	11121	65	81
*M. nonchromogenicum*	*M. smegmatis* MC^2^ 155	8822	69	83
*M. malmesburii* sp. nov.	*M. rhodesiae* NBB3	8503	52	81
*M. komanii* sp. nov.	*Mycobacterium* sp.JLS	5565	45	81

### Genome assembly visualization by alignment to reference genomes (i.e., *M. rhodesiae, M. smegmatis* MC^2^155, *M. tuberculosis* H37Rv, and *Mycobacterium*. sp. JLS)

**Figure 1:**
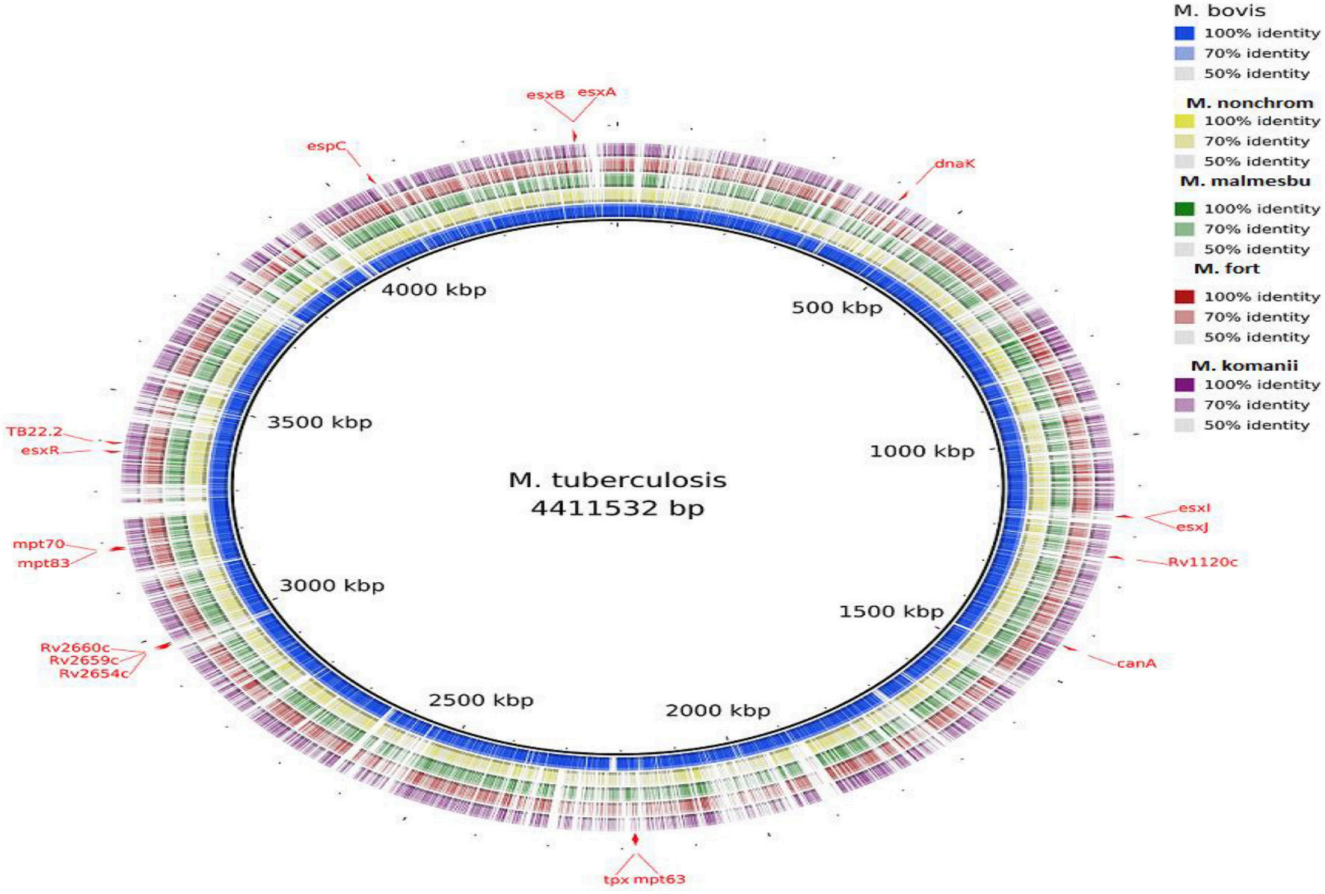
**Alignment of ***M. bovis*** and NTM predicted CDS regions to those of ***M. tuberculosis*** visualized using BRIG**. Immunogenic proteins of interest are highlighted in red (also see Table 1).

**Figure 2:**
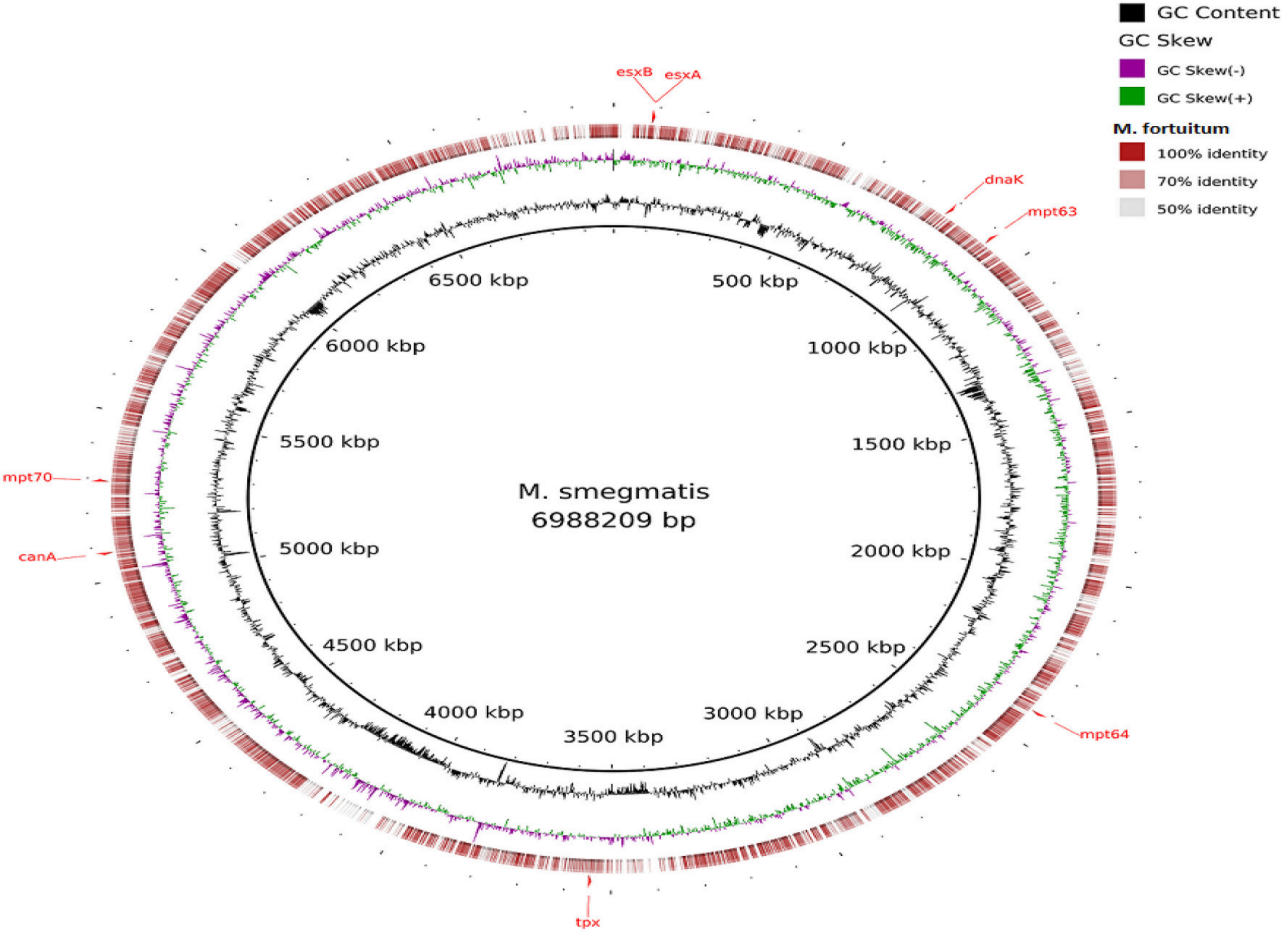
**Alignment of ***M. fortuitum*** predicted CDS regions to those of ***M. smegmatis*** MC^**2**^ 155 (CP001663.1) using BRIG**. Immunogenic proteins of interest identified in M. smegmatis are highlighted in red (esxB: locus tag MSMEG_0065, product hypothetical protein; esxA: locus tagMSMEG_0066, product early secretory antigenic target, 6 kDa; dnaK: locus tag MSMEG_0709, product of chaperone protein DnaK; mpt63: locus tag MSMEG_0828, product immunogenic protein MPT63; mpt64: locus tagMSMEG_2331, product immunogenic protein MPB64/MPT64; mpt70: locus tag MSMEG_5196, product fasciclin domain-containing protein; canA: locus tag MSMEG_4985, product carbonic anhydrase; tpx: locus tagMSMEG_3479, product thiol peroxidase).

**Figure 3:**
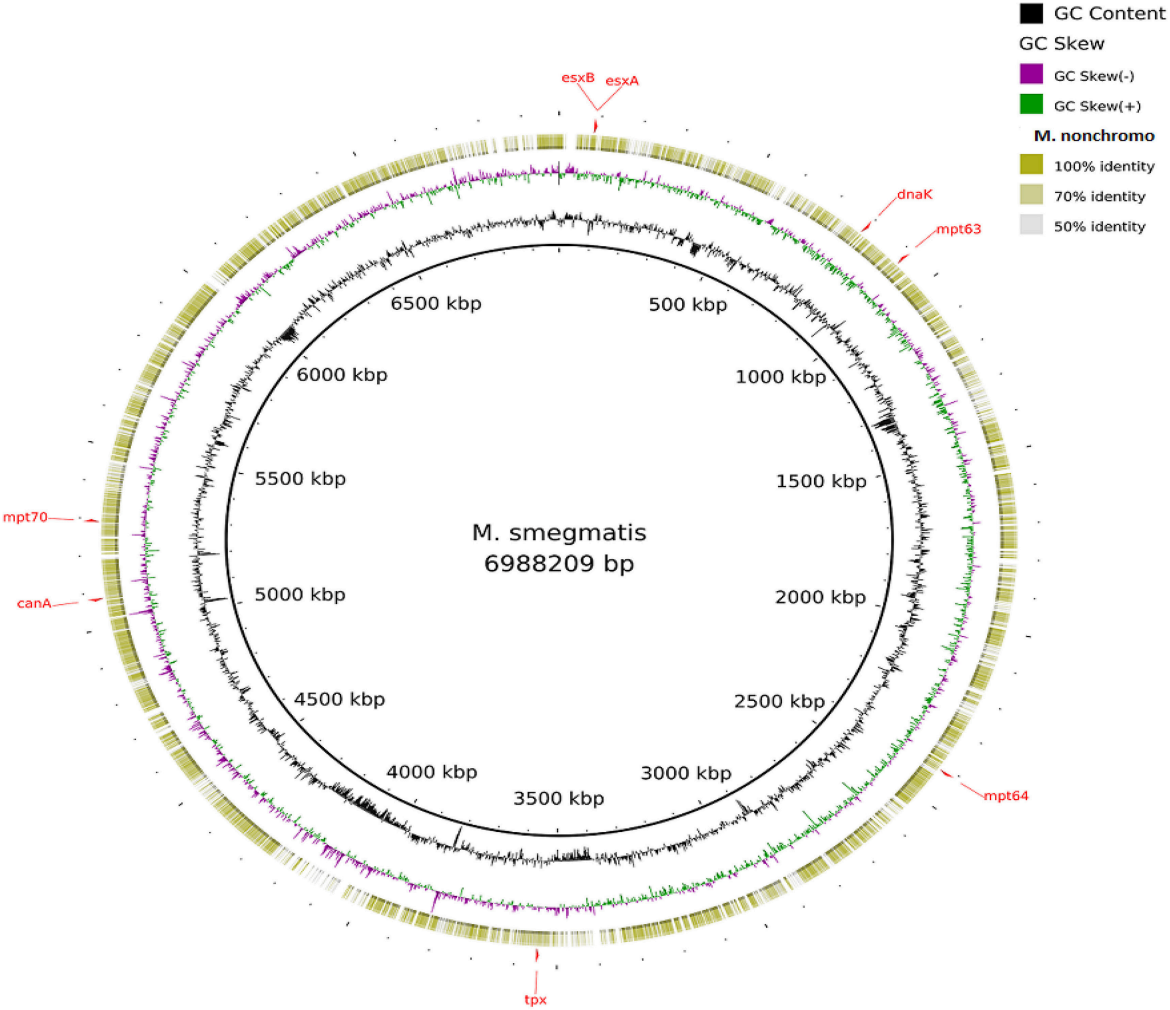
**Alignment of ***M. nonchromogenicum*** predicted CDS regions to those of ***M. smegmatis*** MC^**2**^ 155 (CP001663.1) using BRIG**. Immunogenic proteins of interest identified in M. smegmatis are highlighted in red (esxB: locus tag MSMEG_0065, product hypothetical protein; esxA: locus tagMSMEG_0066, product early secretory antigenic target, 6 kDa; dnaK: locus tag MSMEG_0709, product of chaperone protein DnaK; mpt63: locus tag MSMEG_0828, product immunogenic protein MPT63; mpt64: locus tagMSMEG_2331, product immunogenic protein MPB64/MPT64; mpt70: locus tag MSMEG_5196, product fasciclin domain-containing protein; canA: locus tag MSMEG_4985, product carbonic anhydrase; tpx: locus tagMSMEG_3479, product thiol peroxidase).

**Figure 4:**
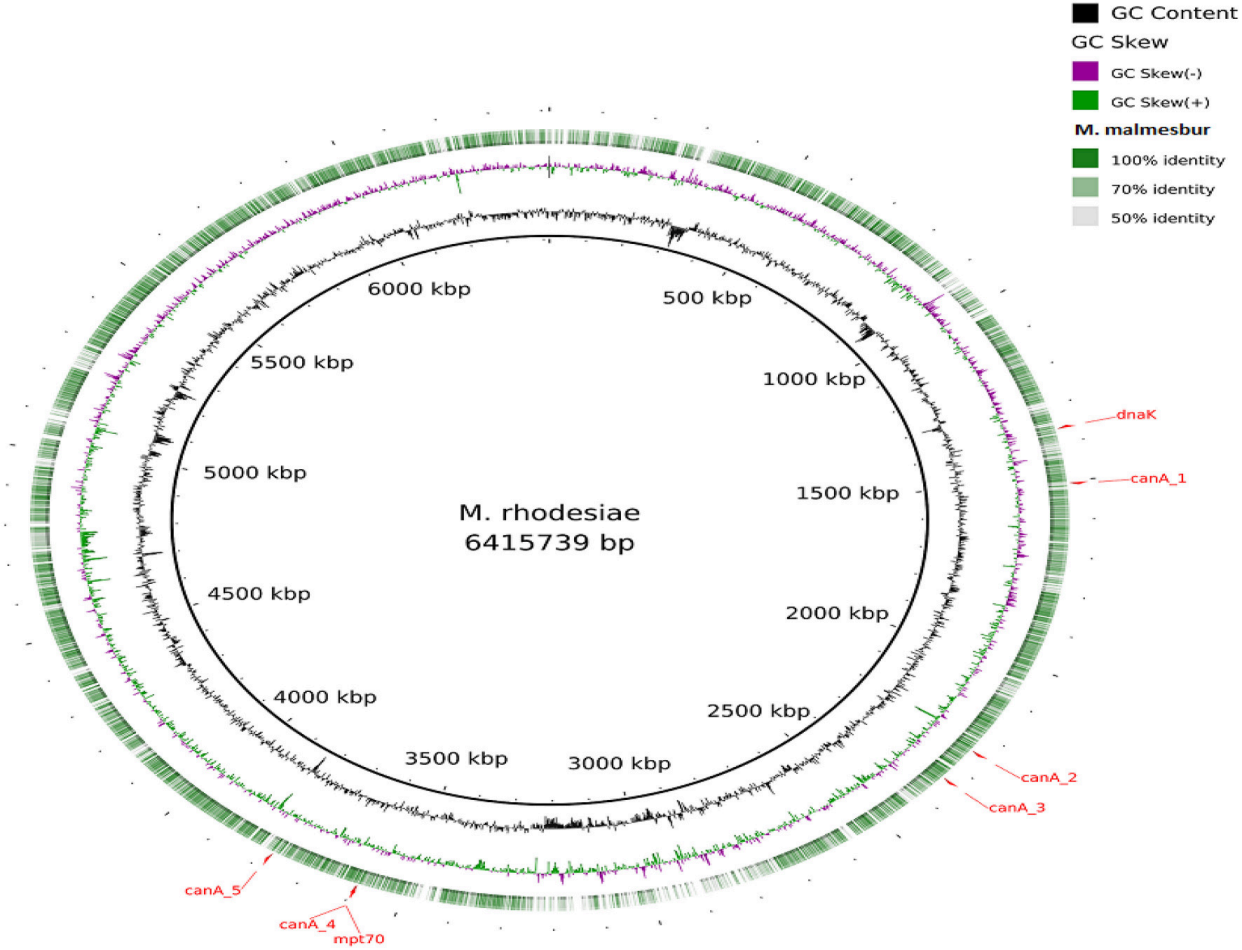
**Alignment of ***M. malmesburii*** sp. nov. predicted CDS regions to those of ***M. rhodesiae*** NBB3 (CP003169.1) using BRIG**. Immunogenic proteins of interest identified in M. rhodesiae are highlighted in red (dnaK: locus tagMycrhN_1341, product chaperone protein DnaK; mpt70: locus tag MycrhN_3596, product secreted/surface protein with fasciclin-like repeats; canA_1: locus tag MycrhN_1479, product sulfate permease-like transporter, MFS superfamily; canA_2: locus tag MycrhN_2217, product carbonic anhydrase; canA_3: locus tagMycrhN_2307, product isoleucine patch superfamily enzyme, carbonic anhydrase/acetyltransferase; canA_4: locus tag MycrhN_3599, product isoleucine patch superfamily enzyme, carbonic anhydrase/acetyltransferase; canA_5: locus tag MycrhN_3776, product carbonic anhydrase. Carbonic anhydrase genes have been numbered according to genomic position).

**Figure 5:**
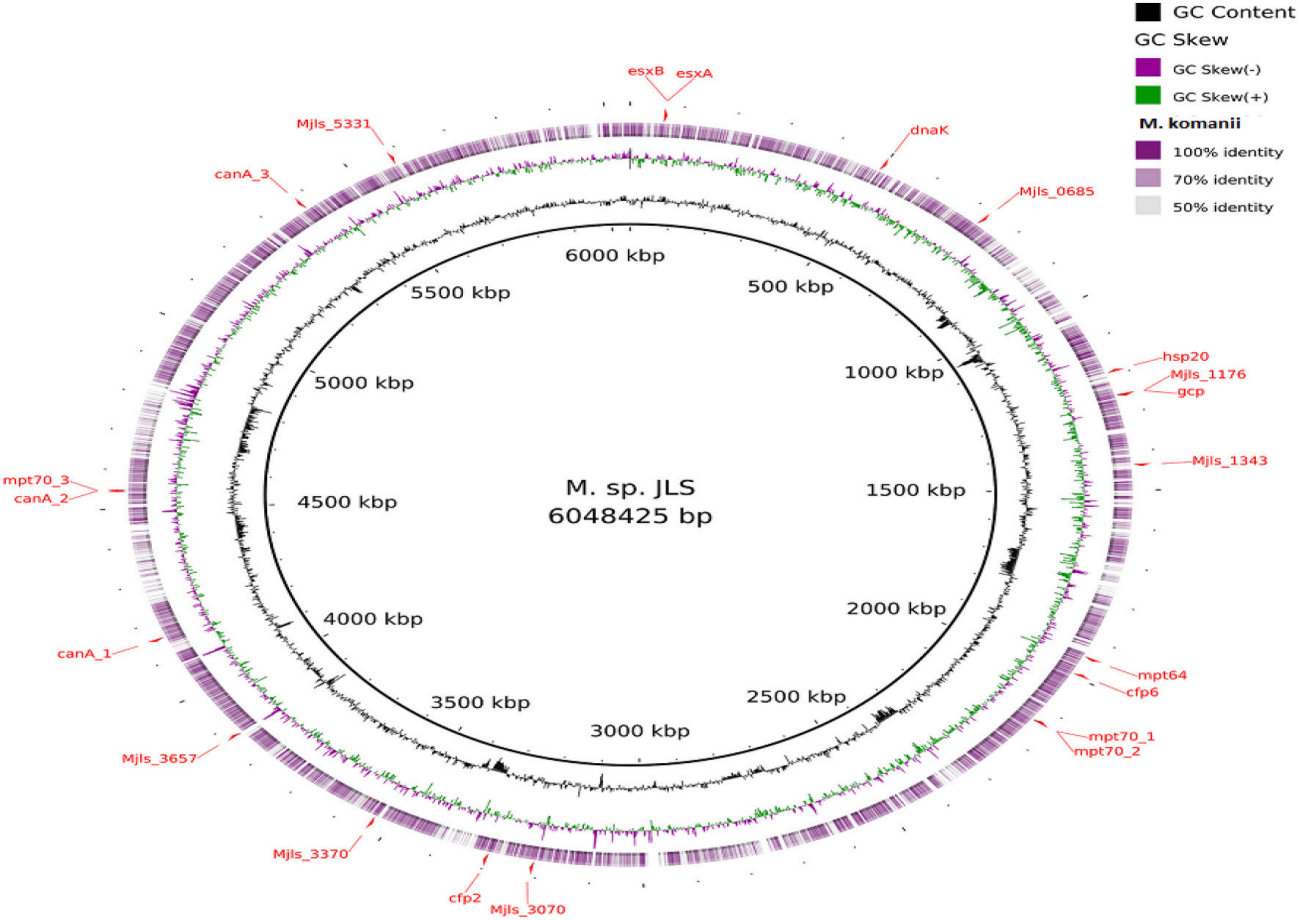
**Alignment of ***M. komanii*** sp. nov. predicted CDS regions to those of M. sp. JLS (CP000580.1) using BRIG**. Immunogenic proteins of interest identified in M. sp. JLS are highlighted in red (esxB: locus tag Mjls_0060, product hypothetical protein; esxA: locus tag Mjls_0061, product 6 kDa early secretory antigenic targetEsaT6; hsp20: locus tag Mjls_1109, product heat shock protein Hsp20; Mjls_1343: locus tag Mjls_1343, product of polysaccharide biosynthesis protein; cfp6: locus tag Mjls_1885, product low molecular weight protein antigen 6; cfp2: locus tag Mjls_3145, product low molecular weight antigen; Mjls_5331: locus tag Mjls_5331, product lipoprotein antigen family protein; dnaK: locus tag Mjls_0449, product molecular chaperone DnaK;mpt64: locus tag Mjls_1842, product immunogenic protein MPB64/MPT64; mpt70_1: locus tag Mjls_2023, product beta-Ig-H3/fasciclin; mpt70_2: locus tag Mjls_2024, product beta-Ig-H3/fasciclin; mpt70_3: locus tagMjls_4307, product beta-Ig-H3/fasciclin; Mjls_1176: locus tag Mjls_1176, product peptidase M22, glycoprotease; gcp: locus tag Mjls_1178, product putative DNA-binding/iron metalloprotein/AP endonuclease; canA_1: locus tag Mjls_3936, product carbonic anhydrase; canA_2: locus tag Mjls_4306, product carbonic anhydrase;canA_3: locus tag Mjls_5131, product carbonic anhydrase; Mjls_0685: locus tag Mjls_0685, product alkyl hydro peroxide reductase/ Thiol specific antioxidant/ Mal allergen; Mjls_3070: locus tag Mjls_3070, product alkyl hydro peroxide reductase; Mjls_3370: locus tag Mjls_3370, product alkyl hydro peroxide reductase; Mjls_3657: locus tag Mjls_3657, product alkyl hydro peroxide reductase.

Visualization of the alignment of the *M. bovis* and the predicted CDS regions of the four NTM species, i.e., *M. nonchromogenicum, M. malmesburii* sp. nov., *M. komanii* sp. nov., and *M. fortuitum* to those of *M. tuberculosis* H37Rv using BLAST Ring Image Generator (BRIG) is given in Figure 1, while alignment of each NTM predicted CDS to their closest relatives is shown by the images in Figure 2 (*M. fortuitum* and *M. smegmatis* MC^2^155), Figure 3 (*M. nonchromogenicum* and *M. smegmatis* MC^2^155), Figure 4 (*M. malmesburii* sp. nov. and *M. rhodesiae*), and Figure 5 (*M. komanii* sp. nov. and *Mycobacterium* sp. JLS). The positions of the immunogenic proteins of interest in the reference genomes are also highlighted.

### Orthologs of the Esx and PE/PPE family proteins situated within the ESX-1 to ESX-5 regions

**Figure 6:**
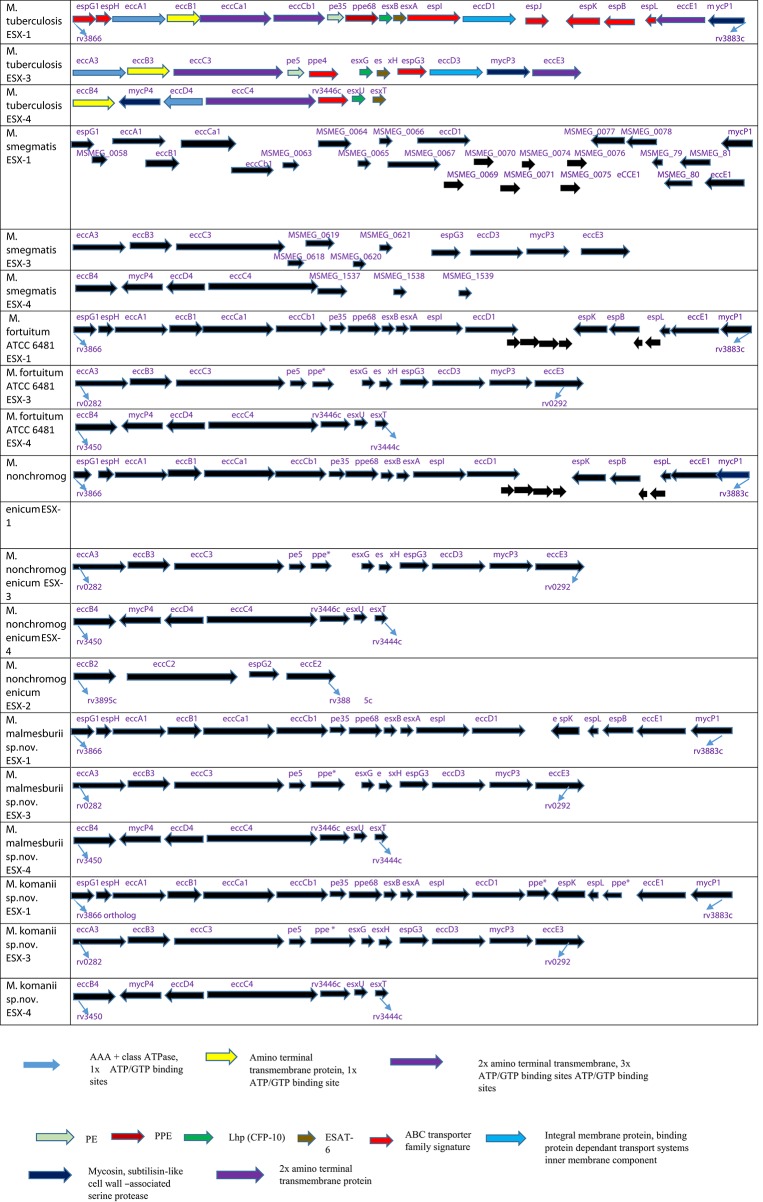
**Schematic representation of the genomic arrangement of orthologs of genes present in three ESAT-6 gene cluster regions: ESX-1, ESX-3, and ESX-4 of ***M. fortuitum, M. malmesburii*** sp. nov., ***M. nonchromogenicum*** (as well as ESX-2), and ***M. komanii*** sp. nov., compared to ***M. tuberculosis*** and ***M. smegmatis*****. *M. tuberculosis* annotation was also used for the NTM orthologs (except for *M. smegmatis*). Protein coding sequences are represented by blocked arrows reflecting the relative lengths of the genes and the direction of transcription. Genes of *M. tuberculosis* are color coded according to the protein families they encode for (Gey van Pittius et al., 2006). The unannotated genes in NTM encode for hypothetical proteins.

The orthologs of the Esx and PE/PPE family proteins (situated within the ESX-1 to ESX-5 regions) deducted from the newly sequenced NTM genomes are listed in Table 6. The ESX-1, ESX-3 and ESX-4 loci were found in all four of the newly annotated NTM genomes, while the ESX-2 and ESX-5 loci seem to be absent in three of the NTM genomes (*M. fortuitum, M. malmesburii* sp. nov., and *M. komanii* sp. nov). This is consistent with what was observed in the genomes of other rapidly growing NTM like *M. smegmatis, Mycobacterium* sp. JLS, *Mycobacterium vanbaalenii, Mycobacterium vaccae, Mycobacterium* sp. MCS, *Mycobacterium thermoresistible, Mycobacterium* sp. KMS, and *M. flavescens* where only three of the ESAT-6 loci (ESX-1, ESX-3, and ESX-4) were found to be present (Gey van Pittius et al., 2001, 2006; Newton-Foot et al., 2016). In-order to confirm the orthology of the NTM *esx* and *pe/ppe* genes to those of *M. bovis*/*M. tuberculosis* as well as the potential of their protein products to be secreted, we also investigated the newly sequenced NTM genomes for the adjacent genes situated in the ESX loci, as well as the genomic organization of these loci. The organization of these genes in the ESX- loci is illustrated in Figure 6. *EsxA* and *esxB* as well as *esxG* and *esxH* were found next to each other in the genomes of the newly sequenced NTM. The *pe35* and *ppe68* genes were found to occur next to each other in the genomes of the four newly sequenced NTM. One member of the *pe5/ppe4* pair (*ppe4*), was not detected in any of the newly sequenced NTM. Comparing the ESX loci of *M. tuberculosis* and *M. bovis* to those of the newly sequenced NTM, we found that, all the genes of the ESX-1 locus and their organization (as known to occur in *M. tuberculosis* and *M. bovis*) except *espJ* (whose function is unknown), were found in all four newly sequenced NTM as shown in Figure 6. Likewise all the genes of the ESX-3 (except *ppe4*) and ESX-4 loci (as known to occur in *M. tuberculosis* and *M. bovis*) were also found in all four newly sequenced NTM (Figure 6). The only NTM species that was found to harbor an Esx ortholog of the ESX-5 locus (*esxN*) as well as 4 of the 12 ESX-2 genes was *M. nonchromogenicum*.

**Table 6:** **Orthologs of the Esx and PE/PPE family proteins in the four NTM genomes**.

**Locus**	**esx/pe/ppe gene tag**	**Percentage homology of Esx and PE/PPE amino acid sequences deducted from the different NTM to** ***M. bovis***			
		***M. komanii sp. nov.***	***M. malmesburii sp. nov***	***M. fortuitum***	***M. nonchromogenicum***
**ESX-1**	esxA/Rv3875/Mb3905	48.75	50	75.79	71.58
	esxB/Rv3874/Mb3905	57.73	57.73	64	64
	pe35/Rv3872/Mb3902	51.55	51.55	52.22	52.22
	ppe68/Rv3873/Mb3903	63.65	47.79	59.44	56.10
**ESX-2**	esxC/Rv3890c/Mb3919c	no	no	no	no
	esxD/Rv3891c/Mb3920c	no	no	no	no
	ppe69/Rv3892c/Mb3921c	no	no	no	no
	pe36/Rv3893c/Mb3922c	no	no	no	no
**ESX-3**	esxG/Rv0287/Mb0295	78.35	78.35	81.5	81.05
	esxH/Rv0288/Mb0296	76	76	75.79	77.94
	pe5/Rv0285/Mb0293	72.28	72.28	74.54	74.51
	ppe4/Rv0286/Mb0294	no	no	no	no
**ESX-4**	esxT/Rv3444c/Mb3474c	73.4	73.96	73.96	73.96
	esxU/Rv3445c/Mb3475c	60.75	56.01	60.42	60.42
**ESX-5**	esxM/Rv1792/Mb1820	no	no	no	no
	esxN/Rv1793/Mb1821	no	no	no	85.11
	ppe25/Rv1787/Mb1815	no	no	no	no
	pe18/Rv1788/Mb1816	no	no	no	no
	ppe26/Rv1789/Mb1817	no	no	no	no
	ppe27/Rv1790/Mb1818	no	no	no	no
	pe19/Rv1791/Mb1819	no	no	no	No

*no, no ortholog detected*.

To quantify amino acid sequence homology of the EsxA and EsxB of *Mycobacterium fortuitum* in this study to other species in the NCBI database we conducted BLASTP searches. Interestingly, the BLAST searches using the *M. fortuitum* EsxA and EsxB amino acid sequences revealed the existence of their orthologs in additional NTM species, most notably *M. vulneris* (98% similarity to *M. fortuitum* at amino acid level for both proteins), *M. mageritense* (82 and 74% similarity to *M. fortuitum* respectively, at amino acid level), and *M. farcinogenes* (96 and 92% similarity to *M. fortuitum* respectively, at amino acid level).

### Verification of the presence the *esx* genes in the newly sequenced NTM

The presence of *esxA* and *esxB* were confirmed in *M. fortuitum* by PCR and sequencing using primers designed from *M. smegmatis* sequences followed by BLAST searches. We demonstrated the presence of both these gene fragments in *Mycobacterium fortuitum* ATCC 6841 whose nucleotide sequences were 89 and 85% identical to the *M. smegmatis esxA* and *esxB* orthologs. *M. tuberculosis*/*M. bovis* derived primers did not amplify any of the NTM (results not shown). Likewise the *M. smegmatis* derived primers could not amplify these genes in other NTM (*M. nonchromogenicum, M. malmesburii* sp. nov., and *M. komanii* sp. nov.).

The presence of *esxH* was also confirmed by PCR and sequencing in *M. fortuitum* (74% identical to the *M. smegmatis esxH* ortholog); *M. komanii* sp. nov. (82% identical to the *M. smegmatis* ortholog); and finally in *M. malmesburii* sp. nov., (84% identical to the *M. smegmatis* ortholog). The *M. nonchromogenicum* ortholog did not amplify when these primers were used.

Since DNA-DNA hybridization analysis by Gey van Pittius et al. (2006) previously suggested that ESX-5 may be absent in *M. nonchromogenicum* or the species was evolutionary so far removed from the slow growers that gene homology was insufficient to allow hybridization, we set out to verify the presence of *esxN* in this species by PCR and sequencing using primers designed from the *esxN/rv1793c* gene of *M. tuberculosis*. The highest BLAST hit of the sequenced gene fragment reveals that this sequence was 91% closely related to *Mycobacterium* species JDM601 *esxN* gene.

### Comparison of amino acid sequence of the *M. bovis*/*M. tuberculosis* immunogenic epitopes of ESAT-6, CFP-10, TB10.4, PPE68, to the NTM homologs

#### ESAT-6 (EsxA)

**Figure 7:**
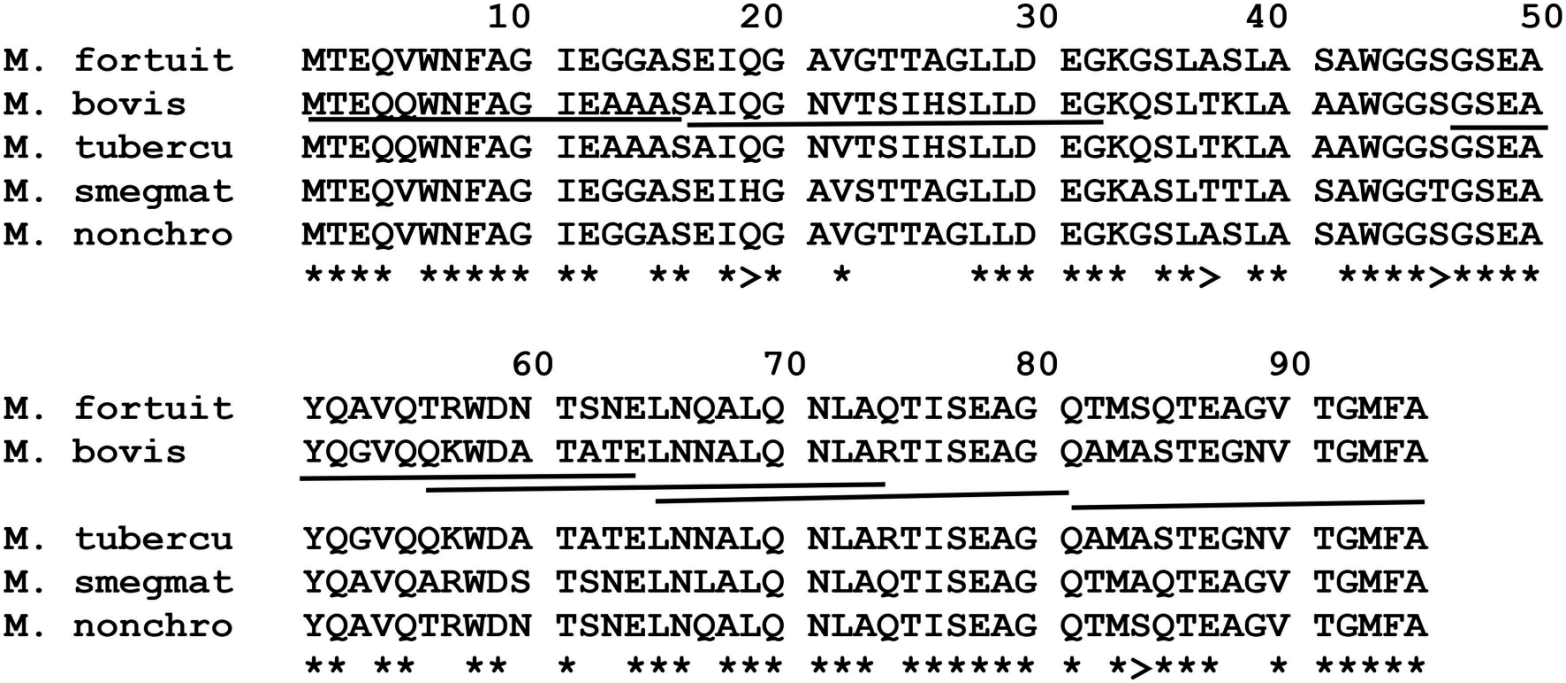
**Alignment of EsxA aa sequences of ***M. fortuitum*** ATCC 6841 (M. fortuit), I MC^**2**^155 (M. smegmat), ***M. bovis***, and ***M. tuberculosis*** (M. tubercu)**. ^*^Represents identical sequences observed in all species, and >indicates presence of the same aa residue in at least one of the NTM species. Highly immunogenic epitopes of *M. bovis* as described by Vordermeier et al. (2000, 2003, 2007) are underlined.

The amino acid sequence alignment of *M. fortuitum, M. nonchromogenicum, M. smegmatis, M. bovis*/*M. tuberculosis* ESAT-6 homologs is demonstrated in Figure 7. Immunogenic epitopes (underlined sequences in Figure 7) of *M. bovis* ESAT-6 as demonstrated by Vordermeier et al. (2000, 2003, 2007) were found in *M. fortuitum, M. nonchromogenicum* as well as in *M. smegmatis* orthologs. Comparison of *M. bovis, M. nonchromogenicum, M. smegmatis*, and *M. fortuitum* immunodominant epitopes revealed the following results: amino acid sequence similarities of 13/16 (81.28%) between the three NTM and *M. bovis* for the epitope at position 1-16; 9/17 (52.9%) and 10/17 (58.8%) amino acid residues were identical between *M. smegmatis* and *M. bovis* and between *M. nonchromogenicum, M. fortuitum*, and *M. bovis* respectively, for the epitope at position 16-32, while 12/18 (66%) amino acid sequence identity was observed between the three NTM and *M. bovis* for the epitope at position 47-64; 12/19 (63%) for the epitope at position 56-74; 15/17 (88%) for the epitope at position 65-81 were identical between the three NTM and *M. bovis*. Finally 11/15 identity between *M. fortuitum, M. nonchromogenicum, and M. bovis*, and 12/15 between *M. smegmatis* and *M. bovis* for epitope at position 81-95. Amino acid sequence similarities of 90 and 95% for the ESAT-6 were observed between *M. smegmatis* and *M. fortuitum* as well as *M. smegmatis* and *M. nonchromogenicum*, respectively.

#### CFP-10 (EsxB)

**Figure 8:**
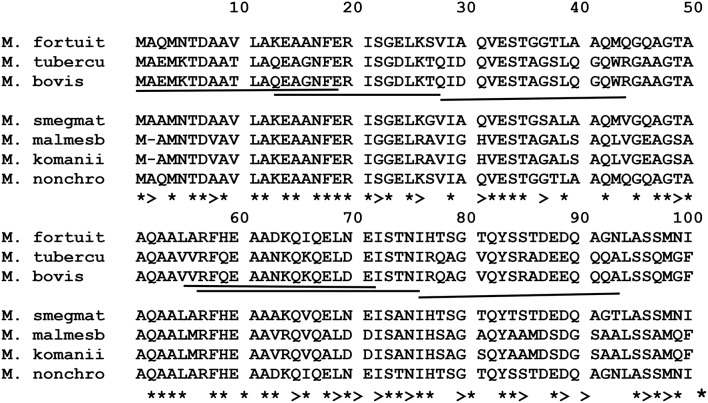
**Alignment of EsxB aa sequences of ***M. fortuitum*** ATCC 6841 (M. fortuit), ***M. smegmatis*** MC^**2**^ 155 (M. smegmat), ***M. malmesburii*** sp. nov (M. malmesb), ***M. komanii*** sp. nov., ***M. bovis***, and ***M. tuberculosis*** (M. tubercu)**. ^*^Represents identical sequences observed in all species, and >indicates presence of the same aa residue in at least one of the NTM species. Highly immunogenic epitopes of *M. bovis* as described by Vordermeier et al. (2000, 2003, 2007) are underlined.

Comparison of amino acid sequences of the CFP-10 homologs of *M. fortuitum, M. nonchromogenicum, M. smegmatis, M. malmesburii* sp. nov, *M. komanii* sp. nov, *M. bovis*, and *M. tuberculosis* is illustrated by the amino acid alignment in Figure 8 and immunogenic epitopes as demonstrated by Vordermeier et al. (2003, 2007) are underlined. Comparing the CFP-10 amino acid sequences of *M. bovis* and the NTM orthologs revealed the following: for the epitope at position 1-19, a total of 14/19 (73.7%) of the amino acid residues were identical between *M. bovis, M. smegmatis, M. nonchromogenicum*, and *M. fortuitum*, while only 12/19 (63%) amino acid residues were identical between *M. bovis, M. komanii* sp. nov., and *M. malmesburii* sp. nov. For the epitope at position 13-28, a total of 11/16 (68.75%) of the amino acids were found to be identical between *M. smegmatis, M. nonchromogenicum, M. fortuitum*, and *M. bovis*, while 10/16 (62.5%) were identical between *M. komanii* sp. nov., *M. malmesburii* p. nov., and *M. bovis*. The epitope at position 28-44 had 9/16 (56.25%) amino acid residues identical between *M. bovis, M. fortuitum, M. nonchromogenicum*, and *M. smegmatis*, while 8/16 (50%) were identical between *M. bovis, M. komanii* sp. nov. and *M. malmesburii* sp. nov. The epitope at positon 55-72 showed sequence identity of 12/16 (75%) of the amino acid residues between *M. bovis, M. fortuitum, M. nonchromogenicum, M. smegmatis, M. komanii* sp. nov., and *M. malmesburii* sp. nov. For the epitope at position 56-76, 12/20 (60%) of the amino acids were identical between all five NTM and *M. bovis*. Finally, for epitope at position 76-93, 9/18 (50%) of the amino acid residues were identical between *M. bovis, M. fortuitum, M. nonchromogenicum*, and *M. smegmatis*, while 6/18 (33.3%) were identical between *M. bovis, M. komanii* sp. nov., and *M. malmesburii* sp. nov.

#### TB10.4 (EsxH)

**Figure 9:**
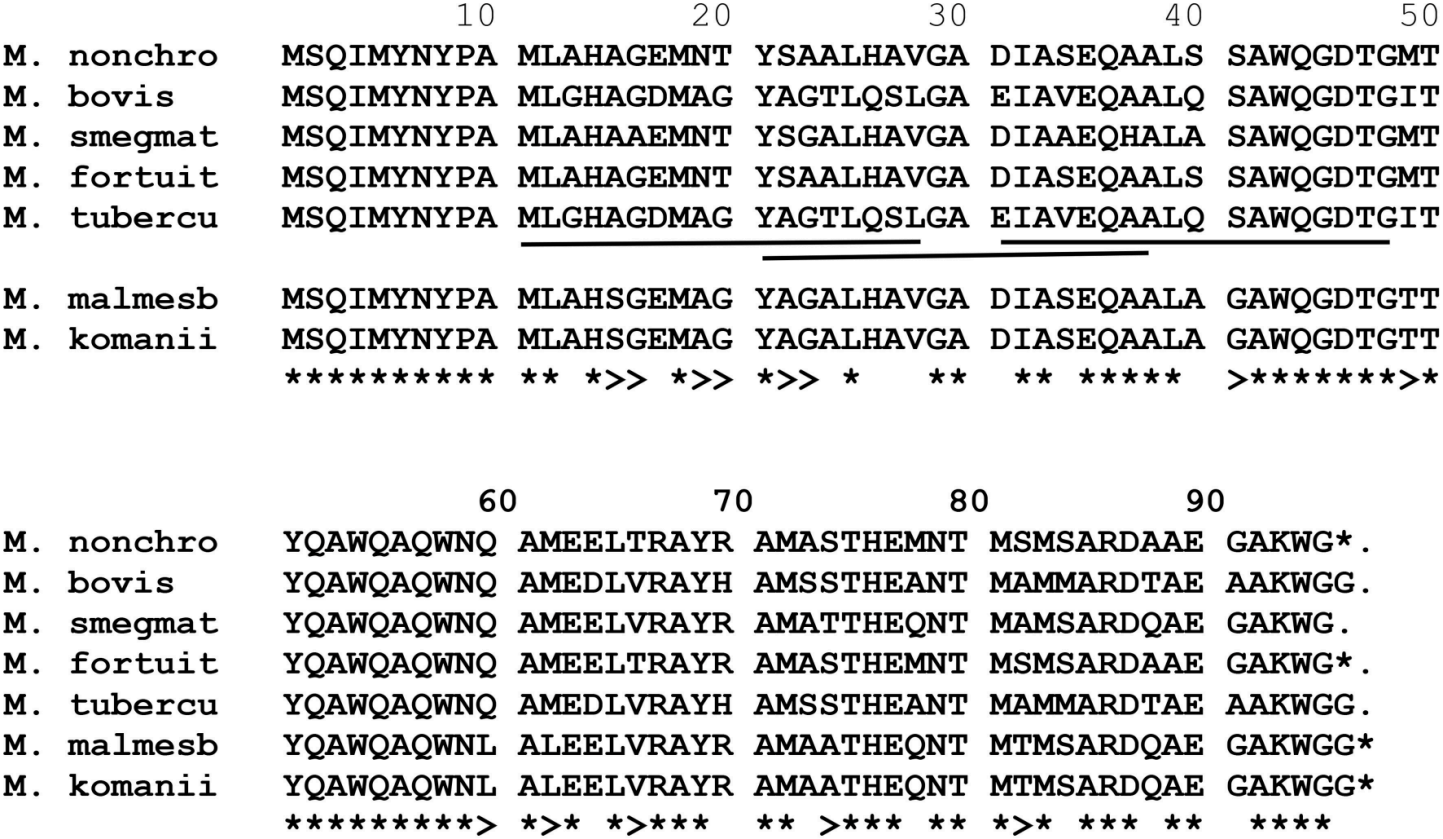
**Alignment of EsxH aa sequences of ***M. fortuitum*** ATCC 6841 (M. fortuit), ***M. smegmatis*** MC^**2**^155 (M. smegmat), ***M. malmesburii*** sp. nov. (M. malmesb), ***M. komanii*** sp. nov., ***M. nonchromogenicum*** (M. nonchro), ***M. bovis***, and ***M. tuberculosis*** (M. tubercu)**. ^*^Represents identical sequences observed in all species, and >indicates presence of the same aa residue in at least one of the NTM species. Highly immunogenic epitopes of *M. tuberculosis* as described by Skjøt et al. (2002) are underlined.

Alignment of the EsxH amino acid sequences of the NTM, *M. bovis*, and *M. tuberculosis* species is illustrated in Figure 9 and immunogenic epitopes described by Skjøt et al. (2002) are underlined. For the epitope at position 11-28, amino acid sequence similarities of 61% were observed between each EsxH orthologs of *M. komanii* sp. nov., *M. malmesburii* sp. nov., and *M. bovis/M. tuberculosis* while *M. nonchromogenicum* and *M. fortuitum* each shared 44.4% similarities with the *M. bovis*/*M. tuberculosis* epitope. For the epitope at position 21-38, 66.7% identity was observed between each of the two NTM species (*M. komanii* sp. nov. and *M. malmesburii* sp. nov.) and *M. tuberculosis*/*M. bovis*. This epitope was 55.5% identical to *M. fortuitum* as well as *M. nonchromogenicum* sequence. Finally, for the epitope at position 31-48, 77.7% amino acid sequence identity was observed between each of *M. komanii* sp. nov. and *M. malmesburii* sp. nov., and *M. tuberculosis*/*M. bovis*. A 83.3% sequence identity for this epitope was seen between each of *M. fortuitum* and *M. nonchromogenicum*, and *M. tuberculosis*/*M. bovis*.

#### PPE68 (Rv3873)

**Figure 10:**
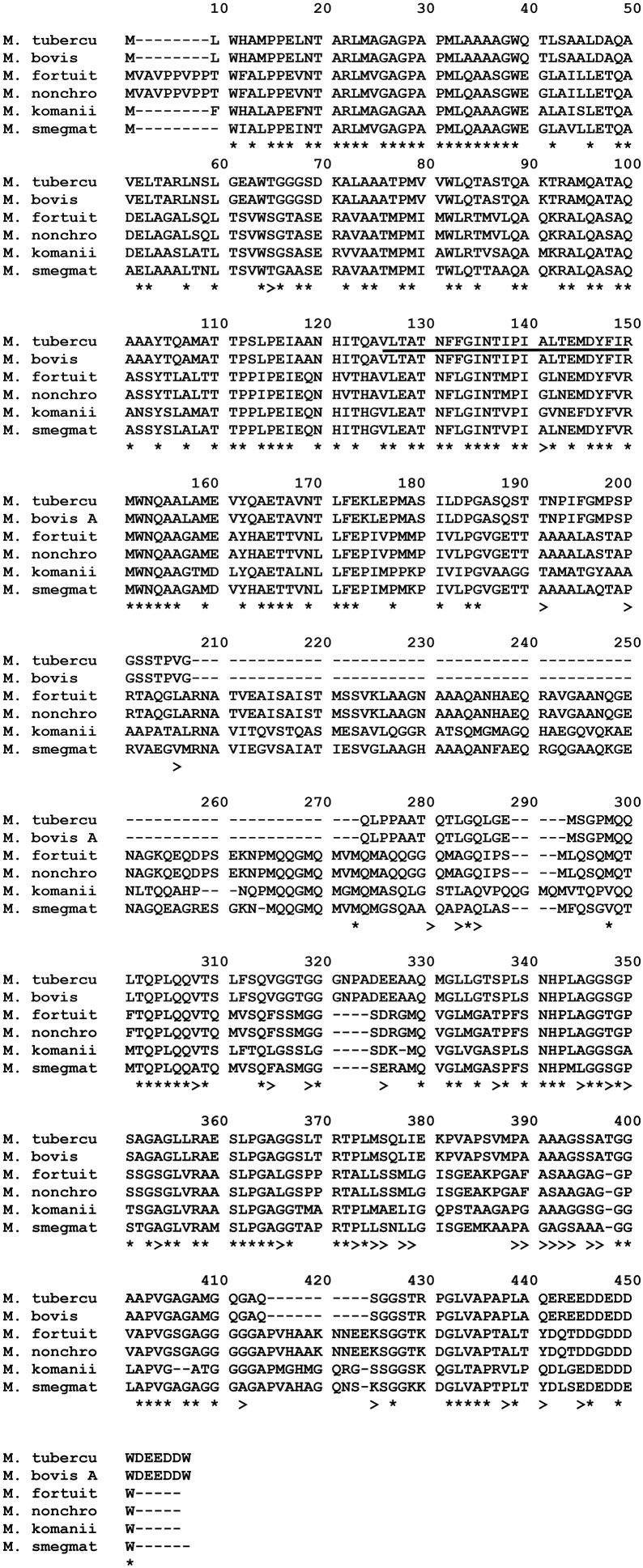
**Alignment of PPE68 aa sequences of ***M. fortuitum*** ATCC 6841 (M. fortuit), ***M. smegmatis*** MC^**2**^ 155 (M. smegmat), ***M. nonchromogenicum*** (M. nonchro), ***M. komanii*** sp. nov., ***M. bovis***, and ***M. tuberculosis*** (M. tubercu)**. ^*^Indicates identical aa sequences in all species, and >indicates same aa residue in at least one of the NTM species and *M. bovis/M. tuberculosis*. Highly immunogenic epitope of *M. tuberculosis*, as described by Mustafa (2014) are Underlined.

Alignment of the corresponding amino acid sequences of *ppe68* orthologs of the NTM, *M. bovis*, and *M. tuberculosis* is illustrated in Figure 10. The immunodominant *M. tuberculosis* epitope “VLTATNFFGINTIPIALTEMDYFIR” described by Mustafa (2014) was also found in *M. fortuitum, M. komanii*, and *M. nonchromogenicum* showing 18/25 (72%) of the amino acid residues to be identical between the NTM and *M. tuberculosis. M. smegmatis* showed 19/25 (76%) amino acid sequence identity of this region to that of *M. tuberculosis*.

### Identification of proteins present in the PPD preparations

**Figure 11:**
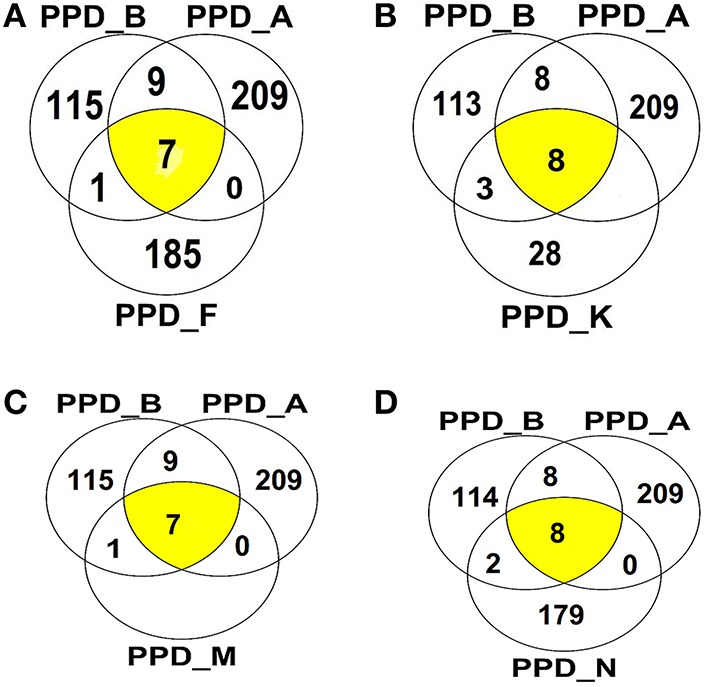
**Venn diagrams illustrating overlap of proteins identified among (A) PPDA-PPD-B, PPD-F, (B) PPD-A, PPD-B, PPD-K, (C) PPD-A, PPD-B, PPD-M, (D) PPD-A, PPD-B, and PPD-N**.

Using Mass spectrometry, 608 different proteins were identified in all the PPD preparations combined (consensus from the different analysis) (Supplementary data). One hundred and thirty two were identified in PPD-B. Among the NTM PPDs, PPD-A showed the highest number of proteins (*n* = 225) followed by PPD-F (*n* = 193), PPD-N (*n* = 189), and PPD-M (*n* = 136) while 39 proteins were identified in PPD-K. The molecular mass of the majority of the proteins was found to be in the range of 10–50 kDA. Twenty two proteins were identified as shared between the NTM PPD preparations and PPD-B (Table 7). Identities and functions of these proteins are also listed in Supplementary Table 8. These proteins include previously characterized immunodominant MTBC proteins like: the 10 kDa culture filtrate protein/CFP-10 (shared between PPD-B and PPD-M); the 10 kDa chaperonin GroES (shared between the NTM PPDs except PPD-M, and PPD-B); 60 kDa chaperonin GroEL (shared by all the NTM PPDs and PPD-B); Chaperone protein DNAK (shared by all the NTM PPDs and PPD-B); Secreted antigen Ag85C (shared between PPD-A and PPD-B), 6 kDa early secretory antigenic target/ESAT-6 (shared between PPD-K and PPD-B) and EsxN (shared between PPD-K and PPD-B). The Venn diagrams in Figure 11 illustrate the number of shared proteins between PPD-B, PPD-A and different NTM PPDs, as well as unique proteins of each PPD. There was a higher degree of protein overlap between PPD-A and PPD-B (16/22 shared proteins) than between the other NTM PPDs and PPD-B. Eight proteins were shared between PPD-B and each of the following NTM PPDs: PPD-M, PPD- while 11 proteins were shared between PPD-B and PPD-K and 10 between PPD-B and PPD-N. One hundred and ten proteins were identified as unique to PPD-B. Four hundred and seventy seven proteins were identified as unique to NTM. Some of the proteins detected only in NTM PPDs have been previously characterized as immunogenic in *M. bovis*/*M. tuberculosis* and were also in the NTM database entries. These include most notably: *M. fortuitum* MPB63 (identified in PPD-F and PPD-N), *M. fortuitum* Ag85C (identified in PPD-F, PPD-M, and PPD-N), *M. fortuitum* Ag85A (identified in PPD-F and PPD-N), *M. kansasii* Ag85B (identified in PPD-K), *M. avium* MPB64 (identified in PPD-A), *M. avium* Ag85A and Ag85B (both identified in PPD-A).

**Table 7:** **Common proteins identified among the six PPD preparations**.

**Identified Proteins (16/561)**	**Accession Number**	**Molecular Weight**	**PPD-A**	**PPD-B**	**PPD-F**	**PPD-K**	**PPD-M**	**PPD-N**	**Function**	**References**
10 kDa culture fíltrate antigen EsxB^*^, *Mycobacterium tuberculosis*	A2VMP9_MYCTX	11 kDa		+			+		Immunodominant antigen involved in secretion, comple × formation, immunogenicity and virulence	Brodin et al., 2005
10 kDa chaperonin GroES^*^, *Mycobacterium tuberculosis*	A2VPK5_MYCTX	11 kDa	+	+	+	+		+	Immunodominant protein, ATP binding	Uniprot, Prasad et al., 2013
60 kDa chaperonin GroEL^*^, *Mycobacterium tuberculosis*	R4LUN2_MYCTX A4KEC8_MYCTX	57 kDa	+	+	+	+	+	+	Immunodominant protein	Uniprot, Prasad et al., 2013
50S ribosomal protein L7/L12, *Mycobacterium parascrofulaceum*	D5PC72_9MYCO	13 kDa	+	+			+		Involved in interaction with translocation factors	Gudkov, 1997
Elongation factor Tu, tuf, *Mycobacterium fortuitum*	K0VK30_MYCFO	44 kDa	+	+	+	+	+	+	GPTase activity	Uniprot
DivIVA domain containing protein, *M. avium* subsp *paratuberculosis*	F7P246_MYCPC	28 kDa	+	+	+	+			Cell shape maintenance	He and de Buck, 2010
Chaperone protein DnaK^*^, *Mycobacterium tuberculosis*	A2VF50_MYCTX	67 kDa	+	+	+	+	+	+	Highly antigenic and act as co-repressor for heat shock protein transcription repressor (hspR)	Das Gupta et al., 2008
Aconitate hydratase, *Mycobacterium. sylvaticum*	V7KF43_MYCAV	102 kDa	+	+			+	+	Unknown function	Uniprot
Elongation factor Tu, *Mycobacterium avium*,	EFTU_MYCA1	44 kDa	+	+			+	+	GTPase activity	Uniprot
Secreted antigen 85-c fbpC^*^, Mycobacterium bovis	A1KEV0_MYCBP	37 kDa	+	+					Proteins of antigen 85 are responsible for high affinity of mycobacteria	Uniprot
Malate synthétase, *Mycobacterium avium*	MASZ_MYCA1	80 kDa	+	+					Enzyme of glyoxylate shunt	Kumar and Bhakuni, 2011
Diacylglycerol acyltransferase/mycolyltransferase Ag85B, *Mycobacterium smegmatis*	A85B_MYCS2	35 kDa	+	+					Proteins of antigen 85 are responsible for high affinity of mycobacteria	Uniprot
Probable glycéraldéhyde 3-phosphate déhydrogénase, *Mycobacterium bovis* BCG	A1KIM5_MYCBP	36 kDa	+	+					Phases of glycolysis	Uniprot
Iron-dependent repressor IdeR, *Mycobacterium avium*	A0QIP3_MYCA1	25 kDa	+	+					DNA binding transcription factor	Uniprot
Putative ESAT-6 like protein 5, esxN ^*^, *Mycobacterium bovis* (strain BCG/Pasteur 1173P2)	A1KJK3_MYCBP	10 kDa		+		+			Reported to induce T-cell response	Deng et al., 2014
DNA-directed RNA polymerase subunit beta', rpoC, *Mycobacterium tuberculosis*	S5ES53_MYCTX	147 kDa		+				+	Catalyzes the transcription of DNA into RNA	Uniprot
30S ribosomal protein, S4, *Mycobacterium fortuitum* subsp fortuitum DSM 46621	KOV712_MYCFO	53 kDa		+	+			+	Binds directly to 16R rRNA, Essential in invitro growth of Mycobacterium tuberculosis	Griffin et al., 2011
6 kDa early secretory antigenic target esxA (Esat-6)^*^, *Mycobacterium tuberculosis*	A2VMQ0_MYCTX	10 kDa		+		+			Immunodominant antigen involved in secretion, complex formation, immunogenicity and virulence	Brodin et al., 2005
ATP synthase subunit beta, *Mycobacterium avium*	A0QCX8|ATPB_MYCA1	53 kDa	+	+	+	+	+	+	Produces ATP from ADP in the presence of a proton gradient across the membrane. The catalytic sites are hosted primarily by the beta subunits	Uniprot
DNA-directed RNA polymerase subunit alpha, rpoA, *Mycobacterium kansasii* ATCC 12478	U5WSX2|U5WSX2_MYCKA	38 kDa	+	+	+	+		+	Catalyzes the transcription of DNA into RNA	Uniprot
Forkhead-associated protein, *Mycobacterium avium* (strain 104)	A0QGN6|A0QGN6_MYCA1	17 kDa	+	+		+			Function is unknown	
Universal stress protein, *Mycobacterium kansasii* ATCC 12478	U5WXR9|U5WXR9_MYCKA	15 kDa		+		+			Response to stress	Uniprot

+, Proteins identified in the respective PPDs; Uniprot, Uniprot database (www.uniprot.org),

* *Previously characterized immunogenic proteins of MTBC*.

## Discussion

In this study we first compared whole genome sequences of four NTM species to those of *M. tuberculosis* and *M. bovis*, mainly focusing on the presence in NTM of genes homologous to those encoding immunogenic proteins of the Esx and the PE/PPE families in *M. bovis*/*M. tuberculosis*. As a second approach, comparative proteomic analysis of PPDs derived from four NTM species to those of commercially available PPDs of *M. bovis* and *M. avium*–was conducted to identify the actual presence of homologous proteins. Three of the investigated NTM species *i.e M. nonchromogenicum, M. malmesburii* sp. nov., and *M. komanii* sp. nov. had previously been described as being among the most abundant NTM in cattle, buffalo and the environments of these animals in South Africa. *M. fortuitum* ATCC 6841 and its PPD was chosen because it is currently used in the modified BOVIGAM™ assay (Michel et al., 2011; Gcebe et al., 2013). *M. malmesburii* sp. nov and *M. komanii* sp. nov. strains used in this study were both isolated from cattle nose mucous membranes and *M. nonchromogenicum* from soil in South Africa (Gcebe et al., 2013). *M. malmesburii* sp. nov. and *M. komanii* sp. nov. are novel rapidly growing mycobacteria (RGM) (colonies taking < 7 days to appear on solid medium). *M. nonchromogenicum* (a slow growing mycobacterial (SGM) species (colonies take >7 days to appear on solid growth medium) and *M. fortuitum* (a RGM) have also been isolated from cattle tissue in Great Britain, France, and Northern Ireland (Pollock and Anderson, 1997; Hughes et al., 2005; Vordermeier et al., 2007; Biet and Boschiroli, 2014) These two species were also reported to be among the most frequently isolated NTM in cattle in other parts of Africa (Diguimbaye-Djaibé et al., 2006; Berg et al., 2009). Exposure of cattle to these NTM species may constitute a possible cause of cross-reactive immune response to PPD and may lead to misdiagnosis of BTB. This further highlighted the need to investigate these NTM in much more detail in terms of their genetic make-up as well as the proteomes of their PPDs in order to assess the presence of immunogenic proteins in NTM potentially causing cross-reactivity with MTBC antigens. Comparative genomics revealed that overall there was very little similarity between the NTM genomes and those of *M. bovis* and *M. tuberculosis*. Despite these differences, orthologs of genes encoding for the most investigated antigens of the Esx family of MTBC including EsxA/ESAT-6, EsxB/CFP-10, EsxH/TB10.4, EsxG/TB 9.8 and EsxN; and the PE/PPE family proteins (PE35, PE5 and PPE68) were identified in the four newly sequenced NTM (Skjøt et al., 2000; Vordermeier et al., 2007; Zvi et al., 2008; Hoang et al., 2013; Amoudy et al., 2014; Mustafa, 2014). Only ESX-1, ESX-3, and ESX-4 loci were detected in the newly sequenced RGM, while genes of ESX-2 as well as *esxN* of ESX-5 were also identified in *M. nonchromogenicum* by whole genome analysis. The presence of *esxA* and *esxB* (situated within the ESX-1 locus) were confirmed in *M. fortuitum* by PCR and sequencing, while *esxN* (situated in ESX-5 locus) was confirmed in *M. nonchromogenicum* also by PCR and sequencing. The *esxA* and *esxB* could not be amplified in *M. nonchromogenicum, M. malmesburii* sp. nov., and *M. komanii* sp. nov. using *M. tuberculosis*/*M. bovis* (result not shown) as well as *M. smegmatis* primers, probably due to huge nucleotide sequence differences between these species. The *esxH* gene (situated in ESX-3 locus) was also confirmed by PCR and sequencing in three of the NTM except *M. nonchromogenicum* using *M. smegmatis* derived primers. This was possibly due to little homology between the *M. nonchromogenicum esxH* sequence and that of *M. smegmatis*, for these primers to anneal. The *esxA, esxB*, and *esxH* genes were however, identified in all the four NTM genomes by whole genome sequence analysis.

Studies have shown that the *esx* genes like *esxA* and *esxB* as well as *esxG* and *esxH* interact to form a 1:1 heterodimer which may be essential for their secretion (Uplekar et al., 2011). Likewise the *pe* and *ppe* genes are thought to form pairs (Gey van Pittius et al., 2006; Uplekar et al., 2011). In this study the *esxA* and *esxB*; *esxG* and *esxH*; *pe35* and *ppe68* genes were found to occur next to each other in the genomes of the four newly sequenced NTM. This confirmed that *esxA, esxB, esxG, esxH, pe35*, and *ppe68* genes in these loci were true orthologs of *M. tuberculosis* and *M. bovis esx, pe*, and *ppe* genes. A closer inspection of the genes adjacent to the *esx, pe*, and *ppe* genes situated within the ESX-loci revealed the occurrence of orthologs of genes encoding for the ATP- dependent chaperones of the AAA family, membrane bound ATPAses, transmembrane proteins chaperonin, and mycosin- subtilin like proteins. These genes form part of the type VII secretory system, therefore if expressed in these NTM, the Esx, PE, and PPE proteins may be secreted.

In NTM; ESAT-6, CFP-10, TB10.4, PE35, PE5, and PPE68 antigens of *M. bovis* and *M. tuberculosis* have been mainly shown to cross-react with homologs of other pathogenic Mycobacterium species that are phylogenetically closely related to MTBC including *M. kansasii, M, marinum, M. ulcerans*, as well as in *M. leprae* (Geluk et al., 2002, 2004; Skjøt et al., 2002; Vordermeier et al., 2007; Amoudy et al., 2014; Mustafa, 2014). Identification of the genes of the Esx family and the PE/PPE family proteins in nonpathogenic NTM in this study add to earlier reports of their occurrence in nonpathogenic *M. smegmatis, Mycobacterium* sp. MCS, *Mycobacterium* sp. JLS, *Mycobacterium phlei, Mycobacterium thermoresistible, Mycobacterium gilvum* PYR-GCK, *Mycobacterium vaccae, Mycobacterium vanbaalenii*, and *Mycobacterium* sp. KMS (Gey van Pittius et al., 2006; Newton-Foot et al., 2016). Expression of the proteins of the Esx family, the PE/PPE family as well as the other antigens in nonpathogenic NTM have not been investigated. For that reason we did comparative investigation for the presence of these proteins in PPDs of three of the nonpathogenic NTM: *M. fortuitum* (PPD-F)*, M. malmesburii* sp. nov. (PPD-M) and *M. nonchromogenicum* (PPD-M) and in addition *M. kansasii* (PPD-K) compared to *M. bovis* (PPD-B) and *M. avium* (PPD-A) by Mass Spectrometric analysis. *Mycobacterium kansasii* derived PPD was included in this study, since its antigens have been investigated for their cross reactivity with *M. bovis* (Vordermeier et al., 2007). This bacterium has been isolated from cattle in Great Britain and the United States of America (Vordermeier et al., 2007; Thacker et al., 2013). Even though proteins are largely degraded during PPD preparation, we identified as many as 609 proteins in the combined six PPDs and we found 22 of these proteins to be shared between PPD-B and one or more of the NTM PPDs, including PPD-A. In the different NTM PPDs we identified homologs of some of previously characterized immunogenic MTBC proteins including CFP-10 (present in PPD-M), GroES (present in all PPDs except PPD-M), GroEL (present in all PPDs), EsxN (PPD-K), ESAT-6 (PPD-K), and DnaK (all PPDs except PPD-M). The occurrence of these immunogenic antigens in nonpathogenic RGM PPDs could potentially lead to cross-reactive immune responses that may interfere with the diagnosis of bovine tuberculosis. In fact these proteins may be responsible for cross-reactivity seen between the *M. fortuitum* PPD and *M. bovis* PPD in South Africa (Michel et al., 2011).

In an attempt to predict cross-reactivity of the NTM homologs of ESAT-6, CFP-10, EsxH, and PPE68 more precisely, we compared amino acid sequences in these proteins with those of defined immunogenic epitopes in the proteins of *M. bovis* and *M. tuberculosis* (Skjøt et al., 2000; Vordermeier et al., 2003, 2007; Mustafa, 2014). This was done despite the fact that some of the genes encoding for these proteins were identified at DNA level, but not represented as proteins in the respective NTM PPD preparations. These proteins, with the exception of *M. malmesburii* sp. nov.'s CFP-10 which was found in PPD-M, could have been degraded during PPD preparation, and possibly differential sensitivity for procedures of PPD production and trypsin treatment before Mass spectrometry or were not abundantly expressed in NTM to allow detection by LC-MS/MS (Borsuk et al., 2009). In all cases as illustrated in the amino acid alignments in Figures 6–10, homologies of >50% of the previously characterized immunogenic epitopes in *M. bovis* were seen in the different NTM homologs. For instance, six epitopes of *M. bovis* ESAT-6 recognized by bovine T-cells in the context of BoLA-DR and BoLA DQ, as described by Vordermeier et al. (2003, 2007) had amino acid sequence homologies of as high as 81.28% and as low as 52.9% to those of the *M. fortuitum* and *M. nonchromogenicum* orthologs. Likewise amino acid sequence homologies of >50% between the four *M. bovis* CFP-10 immunogenic epitopes and those of the NTM species was observed. The same was seen with PPE68 immunogenic epitope (VLTATNFFGINTIPIALTEMDYFIR) described by Mustafa (2014) where 72% of the amino acid residues were identical between the NTM and *M. tuberculosis*. Still with the sequence homology of < 100% of the NTM CFP-10, ESAT-6, PPE68, and EsxH epitopes and the *M. bovis* antigens, we could not unambiguously predicts T- cell cross reactivity. Several studies have reported contrasting results with regards to the relation of sequence identity and cross-reactivity of antigens. For instance, antigen cross-recognition has been observed with *M. leprae* ESAT-6 and CFP-10 on *M. tuberculosis* patients despite very low sequence identity (36 and 40% respectively at amino acid level) to the *M. tuberculosis* homologs (Geluk et al., 2002, 2004). Likewise, Hewinson et al. (2006) also demonstrated that sequence identity between epitopic regions from unrelated otherwise non-homologous mycobacterial antigens of >50% in the 16–20 mer regions indicated cross-reactivity in cattle (Hewinson et al., 2006). Contrary, other peptides that displayed similar degrees of sequence identity were not cross-reactive (Hewinson et al., 2006).

The NTM homologs in this study could potentially induce cross reactivity against MTBC antigens and should be tested in animal experiments.

In conclusion, in this study we identified genes and proteins present in and expressed by members of the *Mycobacterium tuberculosis* complex known as markers for BTB diagnosis in selected nonpathogenic NTM genomes and proteomes. The identification of the genes encoding ESAT-6, CFP-10, PE35, PPE68, and PE5 in nonpathogenic NTM in this study adds to earlier findings of these genes in nonpathogenic like *M. smegmatis* and *Mycobacterium* sp. KMS. Expression of one of the most studied *M. bovis* immunogenic proteins, CFP-10 in *M. malmesburii* sp. nov. and that of other previously characterized MTBC immunogenic proteins such as GroES, DnaK, GroEL was shown by mass spectrometric analysis of the PPDs. We also identified immunogenic epitopes of ESAT-6, CFP-10, EsxH, and PPE68 through genomic analysis, in some or all of the four newly sequenced nonpathogenic NTM, also suggesting the potential of these proteins, to elicit cross reactive immune responses against MTBC antigens. The NTM homologs of the immunogenic proteins need to be further investigated for their cross-reactivity with the *M. bovis* antigens and consequently their interference in BTB assays.

## Author contributions

All authors listed, have made substantial, direct and intellectual contribution to the work, and approved it for publication.

### Conflict of interest statement

The authors declare that the research was conducted in the absence of any commercial or financial relationships that could be construed as a potential conflict of interest.
