# Supplementary material for: Comparative Genomics and Proteomic Analysis of Four Non-tuberculous Mycobacterium Species and Mycobacterium tuberculosis Complex: Occurrence of Shared Immunogenic Proteins
Source: Front Microbiol. 2016 Jun 7;7:795. doi: 10.3389/fmicb.2016.00795 (PMC4894912; doi:10.3389/fmicb.2016.00795)
Supplement: Supplementary file 1 [file Table8.DOCX]

**Table: 8 List of proteins identified from the different PPD preparations**

| **Protein identified** | **Accession number** | **PPD-A** | **PPD-B** | **PPD-F** | **PPD-K** | **PPD-M** | **PPD-N** |
| --- | --- | --- | --- | --- | --- | --- | --- |
| 1. 2,5-diketo-D-gluconic acid reductase OS=Mycobacterium avium subsp. sylvalticum ATCC 49884 GN=dkgA PE=4 SV=1 | V7KCT7_MYCAV | + | 0 | 0 | 0 | 0 | 0 |
| 1. Fatty acid desaturase OS=Mycobacterium fortuitum subsp. fortuitum DSM 46621 GN=MFORT_29479 PE=3 SV=1 | K0UKU9_MYCFO | 0 | 0 | + | 0 | 0 | + |
| 1. Succinyl-CoA ligase [ADP-forming] subunit beta OS=Mycobacterium fortuitum subsp. fortuitum DSM 46621 GN=sucC PE=3 SV=1 | K0UVN8_MYCFO | 0 | 0 | + | 0 | + | + |
| 1. Uncharacterized protein OS=Mycobacterium fortuitum subsp. fortuitum DSM 46621 GN=MFORT_17993 PE=4 SV=1 | K0V3W0_MYCFO | 0 | 0 | + | 0 | + | + |
| 1. LprG protein OS=Mycobacterium fortuitum subsp. fortuitum DSM 46621 GN=MFORT_05138 PE=4 SV=1 | K0VCH2_MYCFO | 0 | 0 | + | 0 | + | + |
| 1. Thioredoxin domain protein OS=Mycobacterium avium (strain 104) GN=MAV_1545 PE=4 SV=1 | A0QCZ3_MYCA1 | + | 0 | 0 | 0 | 0 | 0 |
| 1. **DNA-directed RNA polymerase subunit beta' OS=Mycobacterium tuberculosis EAI5 GN=rpoC PE=3 SV=1** | **S5ES53_MYCTX** | **0** | **+** | **0** | **0** | **0** | **+** |
| 1. Thioredoxin OS=Mycobacterium bovis (strain BCG / Pasteur 1173P2) GN=trxC_2 PE=3 SV=1 | A1KEF8_MYCBP | 0 | + | 0 | 0 | 0 | 0 |
| 1. Membrane protein OS=Mycobacterium avium subsp. silvaticum ATCC 49884 GN=P863_06195 PE=4 SV=1 | V7KQI0_MYCAV | + | 0 | 0 | 0 | 0 | 0 |
| 1. Ribosome-recycling factor OS=Mycobacterium tuberculosis C GN=frr PE=3 SV=1 | A2VLL5_MYCTX | 0 | + | 0 | 0 | 0 | 0 |
| 1. Uncharacterized protein OS=Mycobacterium fortuitum subsp. fortuitum DSM 46621 GN=MFORT_28714 PE=4 SV=1 | K0UM24_MYCFO | 0 | 0 | + | 0 | + | + |
| 1. Acyl carrier protein OS=Mycobacterium fortuitum subsp. fortuitum DSM 46621 GN=acpP PE=3 SV=1 | K0VG08_MYCFO | 0 | 0 | + | 0 | 0 | + |
| 1. Putative molybdenum cofactor synthesis protein OS=Mycobacterium avium (strain 104) GN=MAV_1097 PE=4 SV=1 | A0QBR2_MYCA1 | + | 0 | 0 | 0 | 0 | 0 |
| 1. Heparin-binding hemagglutinin OS=Mycobacterium fortuitum subsp. fortuitum DSM 46621 GN=MFORT_22870 PE=4 SV=1 | K0UQ98_MYCFO | 0 | 0 | + | 0 | + | + |
| 1. 6 kDa early secretory antigenic target OS=Mycobacterium szulgai GN=esat-6 PE=4 SV=1 | B5A908_MYCSZ | 0 | **+** | 0 | 0 | 0 | 0 |
| 1. Cutinase OS=Mycobacterium fortuitum subsp. fortuitum DSM 46621 GN=MFORT_20545 PE=4 SV=1 | K0V6X9_MYCFO | 0 | 0 | + | 0 | + | + |
| 1. Branched-chain amino acid ABC transporter substrate-binding protein OS=Mycobacterium fortuitum subsp. fortuitum DSM 46621 GN=MFORT_27109 PE=4 SV=1 | K0UIH3_MYCFO | 0 | 0 | + | 0 | + | + |
| 1. Succinate dehydrogenase OS=Mycobacterium avium (strain 104) GN=MAV_4910 PE=4 SV=1 | A0QM93_MYCA1 | + | 0 | 0 | 0 | 0 | 0 |
| 1. Uncharacterized protein OS=Mycobacterium avium (strain 104) GN=MAV_2865 PE=4 SV=1 | A0QGL3_MYCA1 | + | 0 | 0 | 0 | 0 | 0 |
| 1. Putative lipoprotein lprA OS=Mycobacterium bovis (strain BCG / Pasteur 1173P2) GN=lprA PE=4 SV=1 | A1KI57_MYCBP | 0 | + | 0 | 0 | 0 | 0 |
| 1. Serine/threonine protein kinase OS=Mycobacterium avium (strain 104) GN=MAV_0017 PE=4 SV=1 | A0Q8T1_MYCA1 | + | 0 | 0 | 0 | 0 | 0 |
| 1. Uncharacterized protein OS=Mycobacterium fortuitum subsp. fortuitum DSM 46621 GN=MFORT_25794 PE=4 SV=1 | K0UY81_MYCFO | 0 | 0 | + | 0 | 0 | 0 |
| 1. Uncharacterized protein OS=Mycobacterium paratuberculosis (strain ATCC BAA-968 / K-10) GN=MAP_0196c PE=4 SV=1 | Q745I9_MYCPA | + | 0 | 0 | 0 | 0 | 0 |
| 1. Uncharacterized protein OS=Mycobacterium avium subsp. paratuberculosis S5 GN=D522_22373 PE=4 SV=1 | L7DEQ1_MYCPC | + | 0 | 0 | 0 | 0 | 0 |
| 1. Fatty acid desaturase, type 2 OS=Mycobacterium fortuitum subsp. fortuitum DSM 46621 GN=MFORT_23050 PE=3 SV=1 | K0UW52_MYCFO | 0 | 0 | + | 0 | 0 | + |
| 1. Protein GrpE OS=Mycobacterium avium subsp. sylvaticum ATCC 49884 GN=grpE PE=3 SV=1 | V7K3P4_MYCAV | + | 0 | 0 | 0 | 0 | 0 |
| 1. NAD-dependent aldehyde dehydrogenase OS=Mycobacterium avium subsp. paratuberculosis S397 GN=MAPs_44030 PE=3 SV=1 | F7P7B3_MYCPC | + | 0 | 0 | 0 | 0 | 0 |
| 1. Transcription termination/antitermination protein NusG OS=Mycobacterium smegmatis JS623 GN=nusG PE=3 SV=1 | L0IPB6_MYCSM | 0 | 0 | + | 0 | 0 | + |
| 1. **A**ntigen 85-C OS=Mycobacterium fortuitum subsp. fortuitum DSM 46621 GN=MFORT_10614 PE=4 SV=1 | K0VH76_MYCFO | 0 | 0 | + | 0 | 0 | + |
| 1. NADH-quinone oxidoreductase subunit C OS=Mycobacterium avium subsp. paratuberculosis S397 GN=nuoC PE=3 SV=1 | F7P9E5_MYCPC | + | 0 | 0 | 0 | 0 | 0 |
| 1. Probable ferredoxin fdxC OS=Mycobacterium bovis (strain BCG / Pasteur 1173P2) GN=fdxC PE=4 SV=1 | A1KHW8_MYCBP | 0 | + | 0 | 0 | 0 | 0 |
| 1. Polyketide synthase OS=Mycobacterium fortuitum subsp. fortuitum DSM 46621 GN=MFORT_11546 PE=4 SV=1 | K0V3Z1_MYCFO | 0 | 0 | + | 0 | 0 | + |
| 1. Uncharacterized protein OS=Mycobacterium fortuitum subsp. fortuitum DSM 46621 GN=MFORT_23237 PE=4 SV=1 | K0VDE3_MYCFO | 0 | 0 | + | 0 | 0 | + |
| 1. Uncharacterized protein OS=Mycobacterium fortuitum subsp. fortuitum DSM 46621 GN=MFORT_00015 PE=4 SV=1 | K0VB17_MYCFO | 0 | 0 | + | 0 | + | + |
| 1. Elongation factor Ts OS=Mycobacterium paratuberculosis (strain ATCC BAA-968 / K-10) GN=tsf PE=3 SV=1 | EFTS_MYCPA | + | 0 | 0 | 0 | 0 | 0 |
| 1. Phosphoribosylformylglycinamidine synthase, PurS protein OS=Mycobacterium avium (strain 104) GN=purS PE=4 SV=1 | A0QAS2_MYCA1 | + | 0 | 0 | 0 | 0 | 0 |
| 1. Transcription elongation factor GreA OS=Mycobacterium tuberculosis str. Haarlem GN=greA PE=3 SV=1 | A4KG15_MYCTX | 0 | + | 0 | 0 | 0 | 0 |
| 1. Uncharacterized protein OS=Mycobacterium fortuitum subsp. fortuitum DSM 46621 GN=MFORT_30474 PE=4 SV=1 | K0V1X8_MYCFO | 0 | 0 | + | 0 | 0 | + |
| 1. Uncharacterized protein OS=Mycobacterium fortuitum subsp. fortuitum DSM 46621 GN=MFORT_13860 PE=4 SV=1 | K0V3J8_MYCFO | 0 | 0 | + | 0 | 0 | + |
| 1. Uncharacterized protein OS=Mycobacterium fortuitum subsp. fortuitum DSM 46621 GN=MFORT_17021 PE=4 SV=1 | K0V4Y4_MYCFO | 0 | 0 | + | 0 | 0 | + |
| 1. Major membrane protein 1 OS=Mycobacterium avium (strain 104) GN=MAV_2054 PE=4 SV=1 | A0QED3_MYCA1 | + | 0 | 0 | 0 | 0 | 0 |
| 1. 35 kDa protein OS=Mycobacterium tuberculosis (strain ATCC 25177 / H37Ra) GN=MRA_2770 PE=3 SV=1 | 35KD_MYCTA | 0 | + | 0 | 0 | 0 | 0 |
| 1. F420-dependent glucose-6-phosphate dehydrogenase OS=Mycobacterium fortuitum GN=fgd PE=3 SV=1 | FGD_MYCFO | + | 0 | + | 0 | 0 | + |
| 1. Esterase, , antigen 85-A OS=Mycobacterium bovis BCG str. Korea 1168P GN=K60_039460 PE=4 SV=1 | M1IRK4_MYCBI | 0 | + | 0 | 0 | 0 | 0 |
| 1. Uncharacterized protein OS=Mycobacterium fortuitum subsp. fortuitum DSM 46621 GN=MFORT_00599 PE=4 SV=1 | K0VEG0_MYCFO | 0 | 0 | + | 0 | + | + |
| 1. Low molecular weight antigen cfp2 OS=Mycobacterium bovis (strain BCG / Pasteur 1173P2) GN=cfp2 PE=4 SV=1 | A1KL66_MYCBP | 0 | + | 0 | 0 | 0 | 0 |
| 1. Cfp10 (Fragment) OS=Mycobacterium riyadhense GN=cfp10 PE=4 SV=1 | B2CXA0_9MYCO | 0 | + | 0 | 0 | 0 | 0 |
| 1. Uncharacterized protein OS=Mycobacterium avium subsp. paratuberculosis S397 GN=MAPs_40010 PE=4 SV=1 | F7P667_MYCPC | + | 0 | 0 | 0 | 0 | 0 |
| 1. Ketol-acid reductoisomerase OS=Mycobacterium tuberculosis (strain KZN 1435 / MDR) GN=ilvC PE=3 SV=1 | C6DW46_MYCTK | 0 | + | 0 | 0 | 0 | 0 |
| 1. Esterase OS=Mycobacterium fortuitum subsp. fortuitum DSM 46621 GN=MFORT_23557 PE=4 SV=1 | K0UPB9_MYCFO | 0 | 0 | + | 0 | + | + |
| 1. Adenosylhomocysteinase OS=Mycobacterium avium subsp. paratuberculosis S5 GN=ahcY PE=3 SV=1 | L7DDN5_MYCPC | + | 0 | 0 | 0 | 0 | 0 |
| 1. Glyceraldehyde-3-phosphate dehydrogenase, type I OS=Mycobacterium avium (strain 104) GN=gap PE=3 SV=1 | A0QHY5_MYCA1 | + | 0 | 0 | 0 | 0 | 0 |
| 1. Immunogenic protein MPB64/MPT64 OS=Mycobacterium avium (strain 104) GN=MAV_3901 PE=4 SV=1 | A0QJH5_MYCA1 | + | 0 | 0 | 0 | 0 | 0 |
| 1. Glycosyl hydrolase family 16 OS=Mycobacterium avium subsp. paratuberculosis S397 GN=MAPs_42630 PE=4 SV=1 | F7P6X5_MYCPC | + | 0 | 0 | 0 | 0 | 0 |
| 1. Putative esterase OS=Mycobacterium vaccae ATCC 25954 GN=MVAC_22785 PE=4 SV=1 | K0UUC4_MYCVA | 0 | 0 | 0 | 0 | + | 0 |
| 1. ATP-dependent Clp protease proteolytic subunit OS=Mycobacterium fortuitum subsp. fortuitum DSM 46621 GN=clpP PE=3 SV=1 | K0VDN3_MYCFO | 0 | 0 | + | 0 | 0 | 0 |
| 1. Transaldolase OS=Mycobacterium fortuitum subsp. fortuitum DSM 46621 GN=tal PE=3 SV=1 | K0UPX8_MYCFO | 0 | 0 | + | 0 | + | + |
| 1. Uncharacterized protein OS=Mycobacterium paratuberculosis (strain ATCC BAA-968 / K-10) GN=MAP_0262 PE=4 SV=1 | Q745Q4_MYCPA | + | 0 | 0 | 0 | 0 | 0 |
| 1. Iron-regulated heparin binding hemagglutinin hbhA OS=Mycobacterium tuberculosis CAS/NITR204 GN=J113_03365 PE=4 SV=1 | R4M5A7_MYCTX | 0 | + | 0 | 0 | 0 | 0 |
| 1. Uncharacterized protein OS=Mycobacterium bovis (strain BCG / Pasteur 1173P2) GN=BCG_3219c PE=4 SV=1 | A1KNJ2_MYCBP | 0 | + | 0 | 0 | 0 | 0 |
| 1. Conserved protein with fha domain, fhaa OS=Mycobacterium bovis (strain ATCC BAA-935 / AF2122/97) GN=fhaa PE=4 SV=1 | Q7U303_MYCBO | 0 | + | 0 | 0 | 0 | 0 |
| 1. 10 kDa culture filtrate antigen CFP-10 OS=Mycobacterium sp. 012931 GN=MMSP_0744 PE=4 SV=1 | S7QYG9_9MYCO | 0 | + | 0 | 0 | 0 | 0 |
| 1. Isocitrate lyase OS=Mycobacterium fortuitum subsp. fortuitum DSM 46621 GN=MFORT_23697 PE=4 SV=1 | K0UV53_MYCFO | 0 | 0 | + | 0 | 0 | 0 |
| 1. Heparin binding hemagglutinin hbha OS=Mycobacterium avium (strain 104) GN=MAV_4675 PE=4 SV=1 | A0QLL5_MYCA1 | + | 0 | 0 | 0 | 0 | 0 |
| 1. Thiosulfate sulfurtransferase OS=Mycobacterium fortuitum subsp. fortuitum DSM 46621 GN=MFORT_17411 PE=4 SV=1 | K0V0H1_MYCFO | 0 | 0 | 0 | 0 | 0 | + |
| 1. Uncharacterized protein OS=Mycobacterium fortuitum subsp. fortuitum DSM 46621 GN=MFORT_03396 PE=4 SV=1 | K0V9L0_MYCFO | 0 | 0 | + | 0 | + | 0 |
| 1. Cutinase Cfp21 OS=Mycobacterium fortuitum subsp. fortuitum DSM 46621 GN=MFORT_20173 PE=4 SV=1 | K0VHN0_MYCFO | 0 | 0 | + | 0 | + | + |
| 1. Phosphate-binding protein PstS (Fragment) OS=Mycobacterium avium 09-5983 GN=O983_17710 PE=3 SV=1 | V7LKA4_MYCAV | + | 0 | 0 | 0 | 0 | 0 |
| 1. Uncharacterized protein OS=Mycobacterium tuberculosis RGTB423 GN=MRGA423_01200 PE=4 SV=1 | H8HY34_MYCTX | 0 | + | 0 | 0 | 0 | 0 |
| 1. Phosphorylase OS=Mycobacterium avium (strain 104) GN=MAV_1549 PE=3 SV=1 | A0QCZ5_MYCA1 | + | 0 | 0 | 0 | 0 | 0 |
| 1. Hydrolase OS=Mycobacterium avium subsp. avium 10-9275 GN=O972_10695 PE=4 SV=1 | V7L2H8_MYCAV | + | 0 | 0 | 0 | 0 | 0 |
| 1. Uncharacterized protein OS=Mycobacterium avium subsp. paratuberculosis S5 GN=D522_16748 PE=4 SV=1 | L7DHI8_MYCPC | + | 0 | 0 | 0 | 0 | 0 |
| 1. Uncharacterized protein OS=Mycobacterium avium (strain 104) GN=MAV_2964 PE=4 SV=1 | A0QGW2_MYCA1 | + | 0 | 0 | 0 | 0 | 0 |
| 1. Uncharacterized protein OS=Mycobacterium fortuitum subsp. fortuitum DSM 46621 GN=MFORT_08336 PE=4 SV=1 | K0VTN4_MYCFO | 0 | 0 | + | 0 | 0 | 0 |
| 1. Elongation factor G OS=Mycobacterium fortuitum subsp. fortuitum DSM 46621 GN=fusA PE=3 SV=1 | K0V804_MYCFO | 0 | 0 | + | 0 | + | + |
| 1. Uncharacterized protein OS=Mycobacterium fortuitum subsp. fortuitum DSM 46621 GN=MFORT_30809 PE=4 SV=1 | K0UQ34_MYCFO | 0 | 0 | + | 0 | 0 | + |
| 1. 3-hydroxyacyl-CoA dehydrogenase OS=Mycobacterium fortuitum subsp. fortuitum DSM 46621 GN=MFORT_10926 PE=3 SV=1 | K0V597_MYCFO | 0 | 0 | + | 0 | 0 | + |
| 1. Uncharacterized protein OS=Mycobacterium fortuitum subsp. fortuitum DSM 46621 GN=MFORT_02889 PE=4 SV=1 | K0V9Y7_MYCFO | 0 | 0 | + | 0 | + | 0 |
| 1. Metallopeptidase, zinc binding protein OS=Mycobacterium fortuitum subsp. fortuitum DSM 46621 GN=MFORT_20965 PE=4 SV=1 | K0UYS2_MYCFO | 0 | 0 | + | 0 | + | + |
| 1. Protein RecA OS=Mycobacterium fortuitum subsp. fortuitum DSM 46621 GN=recA PE=3 SV=1 | K0VHA3_MYCFO | 0 | 0 | 0 | 0 | + | + |
| 1. GatB/Yqey domain protein OS=Mycobacterium avium subsp. avium 10-9275 GN=O972_19940 PE=4 SV=1 | V7KTY5_MYCAV | + | 0 | 0 | 0 | 0 | 0 |
| 1. Uncharacterized protein OS=Mycobacterium avium (strain 104) GN=MAV_2908 PE=4 SV=1 | A0QGQ6_MYCA1 | + | 0 | 0 | 0 | 0 | 0 |
| 1. NlpC/P60 family protein OS=Mycobacterium avium (strain 104) GN=MAV_3208 PE=4 SV=1 | A0QHK2_MYCA1 | + | 0 | 0 | 0 | 0 | 0 |
| 1. 2,5-diketo-D-gluconic acid reductase OS=Mycobacterium kansasii ATCC 12478 GN=dkgA PE=4 SV=1 | U5WVJ4_MYCKA | 0 | 0 | 0 | + | 0 | 0 |
| 1. DivIVA protein OS=Mycobacterium fortuitum subsp. fortuitum DSM 46621 GN=MFORT_02086 PE=3 SV=1 | K0VAA8_MYCFO | 0 | 0 | + | 0 | 0 | 0 |
| 1. **60 kDa chaperonin OS=Mycobacterium tuberculosis str. Haarlem/NITR202 GN=groEL PE=3 SV=1** | **R4M2L4_MYCTX** | **0** | **+** | **+** | **+** | **0** | **0** |
| 1. Uncharacterized protein OS=Mycobacterium fortuitum subsp. fortuitum DSM 46621 GN=MFORT_28534 PE=4 SV=1 | K0UFS1_MYCFO | 0 | 0 | + | 0 | + | 0 |
| 1. Uncharacterized protein OS=Mycobacterium avium (strain 104) GN=MAV_1705 PE=4 SV=1 | A0QDE5_MYCA1 | + | 0 | 0 | 0 | 0 | 0 |
| 1. Hydrolase OS=Mycobacterium avium (strain 104) GN=MAV_3654 PE=4 SV=1 | A0QIU2_MYCA1 | + | 0 | 0 | 0 | 0 | 0 |
| 1. Uncharacterized protein (Fragment) OS=Mycobacterium avium subsp. sylvaticum ATCC 49884 GN=P863_23660 PE=4 SV=1 | V7JXY4_MYCAV | + | 0 | 0 | 0 | 0 | 0 |
| 1. Glutamine synthetase OS=Mycobacterium bovis (strain BCG / Pasteur 1173P2) GN=glnA1 PE=3 SV=1 | A1KKR3_MYCBP | 0 | + | 0 | 0 | 0 | 0 |
| 1. Glyoxalase OS=Mycobacterium fortuitum subsp. fortuitum DSM 46621 GN=MFORT_22715 PE=4 SV=1 | K0VE80_MYCFO | 0 | 0 | + | 0 | + | + |
| 1. Thioredoxin OS=Mycobacterium fortuitum subsp. fortuitum DSM 46621 GN=MFORT_22065 PE=3 SV=1 | K0UWY7_MYCFO | 0 | 0 | 0 | 0 | 0 | + |
| 1. Short-chain dehydrogenase/reductase SDR OS=Mycobacterium fortuitum subsp. fortuitum DSM 46621 GN=MFORT_11296 PE=3 SV=1 | K0V488_MYCFO | 0 | 0 | + | 0 | 0 | + |
| 1. Putative esterase OS=Mycobacterium phlei RIVM601174 GN=MPHLEI_14132 PE=4 SV=1 | I0RQ96_MYCPH | 0 | 0 | 0 | 0 | + | 0 |
| 1. Putative thiosulfate sulfurtransferase OS=Mycobacterium avium (strain 104) GN=MAV_4253 PE=4 SV=1 | A0QKF6_MYCA1 | + | 0 | 0 | 0 | 0 | 0 |
| 1. Cutinase cfp21 OS=Mycobacterium tuberculosis C GN=TBCG_01933 PE=4 SV=1 | A2VJ93_MYCTX | 0 | + | 0 | 0 | 0 | 0 |
| 1. Pyruvate dehydrogenase E1 component OS=Mycobacterium tuberculosis str. Haarlem/NITR202 GN=aceE PE=3 SV=1 | R4LZF6_MYCTX | 0 | + | 0 | 0 | 0 | 0 |
| 1. S-adenosylmethionine synthase OS=Mycobacterium tuberculosis str. Haarlem/NITR202 GN=metK PE=3 SV=1 | R4M544_MYCTX | 0 | + | 0 | 0 | 0 | 0 |
| 1. Catalase-peroxidase OS=Mycobacterium fortuitum subsp. fortuitum DSM 46621 GN=katG PE=3 SV=1 | K0USD3_MYCFO | 0 | 0 | + | 0 | 0 | + |
| 1. Fructose-1,6-bisphosphate aldolase OS=Mycobacterium fortuitum subsp. fortuitum DSM 46621 GN=MFORT_17643 PE=4 SV=1 | K0UZU8_MYCFO | 0 | 0 | + | 0 | + | + |
| 1. Uncharacterized protein OS=Mycobacterium fortuitum subsp. fortuitum DSM 46621 GN=MFORT_12651 PE=4 SV=1 | K0V3A6_MYCFO | 0 | 0 | + | 0 | 0 | 0 |
| 1. Uncharacterized protein OS=Mycobacterium fortuitum subsp. fortuitum DSM 46621 GN=MFORT_04181 PE=4 SV=1 | K0V9C1_MYCFO | 0 | 0 | + | 0 | + | + |
| 1. Uncharacterized protein (Fragment) OS=Mycobacterium fortuitum subsp. fortuitum DSM 46621 GN=MFORT_11656 PE=4 SV=1 | K0VRV3_MYCFO | 0 | 0 | + | 0 | + | + |
| 1. Uncharacterized protein OS=Mycobacterium fortuitum subsp. fortuitum DSM 46621 GN=MFORT_25242 PE=4 SV=1 | K0VAE7_MYCFO | 0 | 0 | + | 0 | 0 | 0 |
| 1. Uncharacterized protein OS=Mycobacterium xenopi RIVM700367 GN=MXEN_15035 PE=4 SV=1 | I0RL64_MYCXE | + | 0 | 0 | 0 | 0 | 0 |
| 1. Chaperone protein HtpG OS=Mycobacterium avium subsp. paratuberculosis S397 GN=htpG PE=3 SV=1 | F7P2R7_MYCPC | + | 0 | 0 | 0 | 0 | 0 |
| 1. Uncharacterized protein OS=Mycobacterium avium (strain 104) GN=MAV_3912 PE=4 SV=1 | A0QJI6_MYCA1 | + | 0 | 0 | 0 | 0 | 0 |
| 1. Putative esterase OS=Mycobacterium phlei RIVM601174 GN=MPHLEI_26607 PE=4 SV=1 | I0RCH3_MYCPH | 0 | 0 | 0 | 0 | + | 0 |
| 1. Fatty acid desaturase OS=Mycobacterium fortuitum subsp. fortuitum DSM 46621 GN=MFORT_23927 PE=3 SV=1 | K0VCH0_MYCFO | 0 | 0 | 0 | 0 | 0 | + |
| 1. Response regulator OS=Mycobacterium marinum str. Europe GN=MMEU_0581 PE=4 SV=1 | S7RIY0_MYCMR | 0 | + | 0 | 0 | 0 | 0 |
| 1. Anti-sigma factor antagonist OS=Mycobacterium avium (strain 104) GN=MAV_0435 PE=3 SV=1 | A0Q9Y1_MYCA1 | + | 0 | 0 | 0 | 0 | 0 |
| 1. Uncharacterized protein OS=Mycobacterium fortuitum subsp. fortuitum DSM 46621 GN=MFORT_26639 PE=4 SV=1 | K0V7Z6_MYCFO | 0 | 0 | + | 0 | + | + |
| 1. Sulfate-binding protein OS=Mycobacterium fortuitum subsp. fortuitum DSM 46621 GN=MFORT_16029 PE=4 SV=1 | K0VPE1_MYCFO | 0 | 0 | + | 0 | + | + |
| 1. Zn-dependent hydrolase OS=Mycobacterium avium 05-4293 GN=O984_14515 PE=4 SV=1 | V7J0S9_MYCAV | + | 0 | 0 | 0 | 0 | 0 |
| 1. Prokaryotic ubiquitin-like protein Pup OS=Mycobacterium avium 10-5581 GN=pup PE=3 SV=1 | V7JJI1_MYCAV | + | 0 | 0 | 0 | 0 | 0 |
| 1. Glyoxalase family protein OS=Mycobacterium avium (strain 104) GN=MAV_4702 PE=4 SV=1 | A0QLP1_MYCA1 | + | 0 | 0 | 0 | 0 | 0 |
| 1. Lipoprotein lprG OS=Mycobacterium tuberculosis C GN=TBCG_01389 PE=4 SV=1 | A2VHU1_MYCTX | 0 | + | 0 | 0 | 0 | 0 |
| 1. Succinate dehydrogenase flavoprotein subunit OS=Mycobacterium xenopi RIVM700367 GN=sdhA PE=4 SV=1 | I0RNQ2_MYCXE | 0 | + | 0 | 0 | 0 | 0 |
| 1. Uncharacterized protein OS=Mycobacterium avium subsp. paratuberculosis S397 GN=MAPs_43810 PE=3 SV=1 | F7P791_MYCPC | + | 0 | 0 | 0 | 0 | 0 |
| 1. Bacterioferritin OS=Mycobacterium bovis (strain BCG / Pasteur 1173P2) GN=bfrA PE=3 SV=1 | A1KJT8_MYCBP | 0 | + | 0 | 0 | 0 | 0 |
| 1. Uncharacterized protein OS=Mycobacterium fortuitum subsp. fortuitum DSM 46621 GN=MFORT_02298 PE=4 SV=1 | K0VA15_MYCFO | 0 | 0 | + | 0 | 0 | + |
| 1. Acetyl-CoA acetyltransferase OS=Mycobacterium fortuitum subsp. fortuitum DSM 46621 GN=MFORT_25574 PE=3 SV=1 | K0UKM1_MYCFO | 0 | 0 | + | 0 | 0 | + |
| 1. Uncharacterized protein OS=Mycobacterium avium (strain 104) GN=MAV_3591 PE=4 SV=1 | A0QIN0_MYCA1 | + | 0 | 0 | 0 | 0 | 0 |
| 1. Methyltransferase OS=Mycobacterium fortuitum subsp. fortuitum DSM 46621 GN=MFORT_21845 PE=4 SV=1 | K0URJ0_MYCFO | 0 | 0 | + | 0 | 0 | + |
| 1. LpqE protein OS=Mycobacterium fortuitum subsp. fortuitum DSM 46621 GN=MFORT_20455 PE=4 SV=1 | K0UTS6_MYCFO | 0 | 0 | + | 0 | 0 | + |
| 1. Uncharacterized protein OS=Mycobacterium fortuitum subsp. fortuitum DSM 46621 GN=MFORT_00255 PE=4 SV=1 | K0VXS8_MYCFO | 0 | 0 | + | 0 | 0 | 0 |
| 1. Sulfonate binding protein OS=Mycobacterium avium (strain 104) GN=MAV_0141 PE=4 SV=1 | A0Q948_MYCA1 | + | 0 | 0 | 0 | 0 | 0 |
| 1. Serine esterase, cutinase family protein OS=Mycobacterium avium (strain 104) GN=MAV_4394 PE=4 SV=1 | A0QKU1_MYCA1 | + | 0 | 0 | 0 | 0 | 0 |
| 1. Uncharacterized protein OS=Mycobacterium fortuitum subsp. fortuitum DSM 46621 GN=MFORT_22160 PE=4 SV=1 | K0URR7_MYCFO | 0 | 0 | 0 | 0 | 0 | + |
| 1. ATP synthase epsilon chain OS=Mycobacterium fortuitum subsp. fortuitum DSM 46621 GN=atpC PE=3 SV=1 | K0UKU8_MYCFO | 0 | 0 | + | 0 | + | + |
| 1. Uncharacterized protein OS=Mycobacterium avium (strain 104) GN=MAV_2933 PE=4 SV=1 | A0QGT1_MYCA1 | + | 0 | 0 | 0 | 0 | 0 |
| 1. Cell division protein FtsZ OS=Mycobacterium kansasii GN=ftsZ PE=3 SV=2 | FTSZ_MYCKA | 0 | 0 | + | 0 | + | + |
| 1. Electron transfer flavoprotein subunit beta OS=Mycobacterium fortuitum subsp. fortuitum DSM 46621 GN=MFORT_03276 PE=4 SV=1 | K0V934_MYCFO | 0 | 0 | + | 0 | + | + |
| 1. Uncharacterized protein OS=Mycobacterium avium (strain 104) GN=MAV_2920 PE=4 SV=1 | A0QGR8_MYCA1 | + | 0 | 0 | 0 | 0 | 0 |
| 1. Transketolase OS=Mycobacterium avium (strain 104) GN=tkt PE=3 SV=1 | A0QHX1_MYCA1 | + | 0 | 0 | 0 | 0 | 0 |
| 1. Uncharacterized protein OS=Mycobacterium bovis (strain BCG / Pasteur 1173P2) GN=BCG_0614 PE=4 SV=1 | A1KG46_MYCBP | 0 | + | 0 | 0 | 0 | 0 |
| 1. ATP-binding protein OS=Mycobacterium kansasii ATCC 12478 GN=MKAN_21685 PE=4 SV=1 | U5WTT7_MYCKA | 0 | 0 | 0 | + | 0 | 0 |
| 1. cAMP-binding protein OS=Mycobacterium avium subsp. paratuberculosis S397 GN=MAPs_36470 PE=4 SV=1 | F7P565_MYCPC | + | 0 | 0 | 0 | 0 | 0 |
| 1. ATP synthase gamma chain OS=Mycobacterium gastri 'Wayne' GN=atpG PE=3 SV=1 | W4I0N8_MYCGS | + | 0 | 0 | 0 | 0 | 0 |
| 1. F0F1 ATP synthase subunit delta OS=Mycobacterium fortuitum subsp. fortuitum DSM 46621 GN=MFORT_26179 PE=3 SV=1 | K0UQV8_MYCFO | 0 | 0 | + | 0 | + | + |
| 1. Uncharacterized protein OS=Mycobacterium fortuitum subsp. fortuitum DSM 46621 GN=MFORT_20128 PE=4 SV=1 | K0UZV6_MYCFO | 0 | 0 | + | 0 | 0 | + |
| 1. 3-hydroxyisobutyryl-CoA hydrolase OS=Mycobacterium avium subsp. hominissuis TH135 GN=echA9 PE=4 SV=1 | T2GNN5_MYCAV | + | 0 | 0 | 0 | 0 | 0 |
| 1. Peptidase, M28 family protein OS=Mycobacterium avium (strain 104) GN=MAV_4738 PE=4 SV=1 | A0QLS7_MYCA1 | + | 0 | 0 | 0 | 0 | 0 |
| 1. Uncharacterized protein OS=Mycobacterium bovis (strain BCG / Pasteur 1173P2) GN=BCG_3298 PE=4 SV=1 | A1KNS1_MYCBP | 0 | + | 0 | 0 | 0 | 0 |
| 1. Uncharacterized protein OS=Mycobacterium canettii CIPT 140070017 GN=BN45_20099 PE=4 SV=1 | L0QTC8_9MYCO | 0 | + | 0 | 0 | 0 | 0 |
| 1. Uncharacterized protein OS=Mycobacterium tuberculosis CAS/NITR204 GN=J113_19405 PE=4 SV=1 | R4MJV0_MYCTX | 0 | + | 0 | 0 | 0 | 0 |
| 1. Isocitrate dehydrogenase OS=Mycobacterium fortuitum subsp. fortuitum DSM 46621 GN=MFORT_14487 PE=3 SV=1 | K0VQD7_MYCFO | 0 | 0 | + | 0 | 0 | + |
| 1. Aminopeptidase N OS=Mycobacterium fortuitum subsp. fortuitum DSM 46621 GN=MFORT_02288 PE=4 SV=1 | K0VWN2_MYCFO | 0 | 0 | + | 0 | 0 | + |
| 1. Uncharacterized protein OS=Mycobacterium fortuitum subsp. fortuitum DSM 46621 GN=MFORT_26984 PE=4 SV=1 | K0V7R0_MYCFO | 0 | 0 | 0 | 0 | 0 | + |
| 1. Universal stress protein family protein OS=Mycobacterium fortuitum subsp. fortuitum DSM 46621 GN=MFORT_24062 PE=4 SV=1 | K0UNK4_MYCFO | 0 | 0 | 0 | 0 | 0 | + |
| 1. Uncharacterized protein OS=Mycobacterium avium (strain 104) GN=MAV_0450 PE=4 SV=1 | A0Q9Z4_MYCA1 | + | 0 | 0 | 0 | 0 | 0 |
| 1. NADH-quinone oxidoreductase subunit I OS=Mycobacterium avium subsp. paratuberculosis S397 GN=nuoI PE=3 SV=1 | F7P9D9_MYCPC | + | 0 | 0 | 0 | 0 | 0 |
| 1. Electron transfer flavoprotein (Alpha-subunit) fixB OS=Mycobacterium tuberculosis C GN=TBCG_02963 PE=4 SV=1 | A2VNJ5_MYCTX | 0 | + | 0 | 0 | 0 | 0 |
| 1. Uncharacterized protein OS=Mycobacterium fortuitum subsp. fortuitum DSM 46621 GN=MFORT_17406 PE=4 SV=1 | K0V4V2_MYCFO | 0 | 0 | 0 | 0 | + | + |
| 1. Thioredoxin OS=Mycobacterium fortuitum subsp. fortuitum DSM 46621 GN=MFORT_20665 PE=3 SV=1 | K0UYZ7_MYCFO | 0 | 0 | + | 0 | 0 | + |
| 1. Uncharacterized protein OS=Mycobacterium avium (strain 104) GN=MAV_3785 PE=4 SV=1 | A0QJ69_MYCA1 | + | 0 | 0 | 0 | 0 | 0 |
| 1. 3-hydroxybutyryl-CoA dehydrogenase OS=Mycobacterium fortuitum subsp. fortuitum DSM 46621 GN=MFORT_09710 PE=4 SV=1 | K0V627_MYCFO | 0 | 0 | + | 0 | + | 0 |
| 1. Serine esterase, cutinase OS=Mycobacterium fortuitum subsp. fortuitum DSM 46621 GN=MFORT_06349 PE=4 SV=1 | K0VBV3_MYCFO | 0 | 0 | 0 | 0 | + | + |
| 1. Superoxide dismutase [Cu-Zn] OS=Mycobacterium fortuitum subsp. fortuitum DSM 46621 GN=MFORT_15984 PE=3 SV=1 | K0V1E6_MYCFO | 0 | 0 | + | 0 | 0 | 0 |
| 1. Trigger factor OS=Mycobacterium fortuitum subsp. fortuitum DSM 46621 GN=tig PE=3 SV=1 | K0V9T0_MYCFO | 0 | 0 | + | 0 | + | + |
| 1. Peptidyl-prolyl cis-trans isomerase OS=Mycobacterium bovis (strain BCG / Pasteur 1173P2) GN=ppiA_1 PE=3 SV=1 | A1KEF0_MYCBP | 0 | + | 0 | 0 | 0 | 0 |
| 1. Uncharacterized protein OS=Mycobacterium avium subsp. avium 10-9275 GN=O972_10610 PE=4 SV=1 | V7L6G1_MYCAV | + | 0 | 0 | 0 | 0 | 0 |
| 1. Uncharacterized protein OS=Mycobacterium avium (strain 104) GN=MAV_2351 PE=4 SV=1 | A0QF66_MYCA1 | + | 0 | 0 | 0 | 0 | 0 |
| 1. Uncharacterized protein OS=Mycobacterium bovis (strain BCG / Pasteur 1173P2) GN=BCG_0853 PE=4 SV=1 | A1KGT4_MYCBP | 0 | + | 0 | 0 | 0 | 0 |
| 1. Glyoxalase OS=Mycobacterium avium subsp. avium 10-9275 GN=O972_06050 PE=4 SV=1 | V7LCD1_MYCAV | + | 0 | 0 | 0 | 0 | 0 |
| 1. Peptidase S1 and S6, chymotrypsin/Hap OS=Mycobacterium fortuitum subsp. fortuitum DSM 46621 GN=MFORT_24657 PE=4 SV=1 | K0V088_MYCFO | 0 | 0 | + | 0 | 0 | + |
| 1. Malate synthase G OS=Mycobacterium fortuitum subsp. fortuitum DSM 46621 GN=glcB PE=3 SV=1 | K0V7X3_MYCFO | 0 | 0 | + | 0 | 0 | 0 |
| 1. Serine hydroxymethyltransferase OS=Mycobacterium fortuitum subsp. fortuitum DSM 46621 GN=glyA PE=3 SV=1 | K0UZ83_MYCFO | 0 | 0 | + | 0 | 0 | + |
| 1. 3-oxoacyl-(Acyl carrier protein) synthase II OS=Mycobacterium fortuitum subsp. fortuitum DSM 46621 GN=MFORT_12856 PE=3 SV=1 | K0VR41_MYCFO | 0 | 0 | 0 | 0 | 0 | + |
| 1. Propionyl-CoA carboxylase subunit beta OS=Mycobacterium fortuitum subsp. fortuitum DSM 46621 GN=MFORT_07736 PE=4 SV=1 | K0V743_MYCFO | 0 | 0 | + | 0 | 0 | + |
| 1. Antigen 85-C OS=Mycobacterium fortuitum subsp. fortuitum DSM 46621 GN=MFORT_05814 PE=4 SV=1 | K0V881_MYCFO | **0** | **0** | **+** | 0 | 0 | 0 |
| 1. [NADP+] succinate-semialdehyde dehydrogenase OS=Mycobacterium fortuitum subsp. fortuitum DSM 46621 GN=MFORT_26309 PE=3 SV=1 | K0UXD1_MYCFO | 0 | 0 | + | 0 | 0 | 0 |
| 1. PROBABLE CONSERVED SECRETED PROTEIN TB22.2 OS=Mycobacterium bovis (strain ATCC BAA-935 / AF2122/97) GN=TB22.2 PE=4 SV=1 | Q7TXE4_MYCBO | 0 | + | 0 | 0 | 0 | 0 |
| 1. Probable cutinase cut5 OS=Mycobacterium bovis (strain BCG / Pasteur 1173P2) GN=cut5 PE=4 SV=1 | A1KQ56_MYCBP | 0 | + | 0 | 0 | 0 | 0 |
| 1. Lipoprotein OS=Mycobacterium tuberculosis (strain ATCC 35801 / TMC 107 / Erdman) GN=lppX PE=4 SV=1 | H8EWC8_MYCTE | 0 | + | 0 | 0 | 0 | 0 |
| 1. Uncharacterized protein OS=Mycobacterium fortuitum subsp. fortuitum DSM 46621 GN=MFORT_23232 PE=4 SV=1 | K0V2K8_MYCFO | 0 | 0 | + | 0 | + | + |
| 1. Fumarate hydratase class II OS=Mycobacterium fortuitum subsp. fortuitum DSM 46621 GN=fumC PE=3 SV=1 | K0V3W5_MYCFO | 0 | 0 | + | 0 | 0 | + |
| 1. Uncharacterized protein OS=Mycobacterium kansasii ATCC 12478 GN=MKAN_13555 PE=4 SV=1 | U5WPP2_MYCKA | 0 | 0 | 0 | + | 0 | 0 |
| 1. Peptidyl-prolyl cis-trans isomerase OS=Mycobacterium fortuitum subsp. fortuitum DSM 46621 GN=MFORT_14592 PE=4 SV=1 | K0V7D3_MYCFO | 0 | 0 | + | 0 | + | 0 |
| 1. Enoyl-CoA hydratase OS=Mycobacterium avium (strain 104) GN=MAV_3689 PE=1 SV=1 | A0QIX8_MYCA1 | + | 0 | 0 | 0 | 0 | 0 |
| 1. Uncharacterized protein OS=Mycobacterium avium subsp. hominissuis TH135 GN=MAH_3320 PE=4 SV=1 | T2GXK2_MYCAV | + | 0 | 0 | 0 | 0 | 0 |
| 1. Putative acyl-CoA dehydrogenase OS=Mycobacterium avium (strain 104) GN=MAV_3935 PE=3 SV=1 | A0QJK8_MYCA1 | + | 0 | 0 | 0 | 0 | 0 |
| 1. Peptidase family protein M13 OS=Mycobacterium avium (strain 104) GN=MAV_4977 PE=4 SV=1 | A0QMG0_MYCA1 | + | 0 | 0 | 0 | 0 | 0 |
| 1. Aldehyde dehydrogenase family protein OS=Mycobacterium avium (strain 104) GN=MAV_2812 PE=3 SV=1 | A0QGG1_MYCA1 | + | 0 | 0 | 0 | 0 | 0 |
| 1. Uncharacterized protein OS=Mycobacterium avium (strain 104) GN=MAV_4907 PE=4 SV=1 | A0QM90_MYCA1 | + | 0 | 0 | 0 | 0 | 0 |
| 1. Cutinase OS=Mycobacterium avium subsp. avium 10-9275 GN=O972_08300 PE=4 SV=1 | V7L8Q7_MYCAV | + | 0 | 0 | 0 | 0 | 0 |
| 1. Putative uncharacterized protein OS=Mycobacterium tuberculosis C GN=TBCG_00190 PE=4 SV=1 | A2VNB6_MYCTX | 0 | + | 0 | 0 | 0 | 0 |
| 1. Uncharacterized protein OS=Mycobacterium avium (strain 104) GN=MAV_4701 PE=4 SV=1 | A0QLP0_MYCA1 | + | 0 | 0 | 0 | 0 | 0 |
| 1. Uncharacterized protein OS=Mycobacterium fortuitum subsp. fortuitum DSM 46621 GN=MFORT_10349 PE=4 SV=1 | K0VSD7_MYCFO | 0 | 0 | + | 0 | 0 | 0 |
| 1. Formamidase OS=Mycobacterium fortuitum subsp. fortuitum DSM 46621 GN=MFORT_00070 PE=4 SV=1 | K0VAV4_MYCFO | 0 | 0 | + | 0 | 0 | 0 |
| 1. 30S ribosomal protein S4 OS=Mycobacterium fortuitum subsp. fortuitum DSM 46621 GN=rpsD PE=3 SV=1 | K0UTQ0_MYCFO | 0 | + | + | 0 | + | + |
| 1. Arylsulfatase, AslA OS=Mycobacterium fortuitum subsp. fortuitum DSM 46621 GN=MFORT_24432 PE=4 SV=1 | K0UNS5_MYCFO | 0 | 0 | + | 0 | 0 | 0 |
| 1. Alpha/beta hydrolase OS=Mycobacterium fortuitum subsp. fortuitum DSM 46621 GN=MFORT_30354 PE=4 SV=1 | K0V1Z3_MYCFO | 0 | 0 | + | 0 | 0 | + |
| 1. Cyclophilin type peptidyl-prolyl cis-trans isomerase OS=Mycobacterium fortuitum subsp. fortuitum DSM 46621 GN=MFORT_07436 PE=3 SV=1 | K0V6T7_MYCFO | 0 | 0 | + | 0 | + | 0 |
| 1. Proteasome subunit alpha OS=Mycobacterium fortuitum subsp. fortuitum DSM 46621 GN=prcA PE=3 SV=1 | K0VA96_MYCFO | 0 | 0 | + | 0 | 0 | + |
| 1. Beta-lactamase OS=Mycobacterium fortuitum subsp. fortuitum DSM 46621 GN=MFORT_08116 PE=4 SV=1 | K0VAM4_MYCFO | 0 | 0 | + | 0 | + | + |
| 1. Uncharacterized protein OS=Mycobacterium fortuitum subsp. fortuitum DSM 46621 GN=MFORT_27605 PE=4 SV=1 | K0UH77_MYCFO | 0 | 0 | + | 0 | 0 | 0 |
| 1. Uncharacterized protein OS=Mycobacterium fortuitum subsp. fortuitum DSM 46621 GN=MFORT_28729 PE=4 SV=1 | K0UTZ4_MYCFO | 0 | 0 | + | 0 | + | 0 |
| 1. Transcription elongation factor GreA OS=Mycobacterium avium subsp. paratuberculosis S397 GN=greA PE=3 SV=1 | F7P3H3_MYCPC | + | 0 | 0 | 0 | 0 | 0 |
| 1. Uncharacterized protein OS=Mycobacterium avium (strain 104) GN=MAV_4288 PE=4 SV=1 | A0QKI9_MYCA1 | + | 0 | 0 | 0 | 0 | 0 |
| 1. Dioxygenase OS=Mycobacterium avium (strain 104) GN=MAV_0540 PE=4 SV=1 | A0QA82_MYCA1 | + | 0 | 0 | 0 | 0 | 0 |
| 1. Glutamine synthetase OS=Mycobacterium avium (strain 104) GN=glnA PE=3 SV=1 | A0QEY3_MYCA1 | + | 0 | 0 | 0 | 0 | 0 |
| 1. Probable cutinase cut2 OS=Mycobacterium bovis (strain BCG / Pasteur 1173P2) GN=cut2 PE=4 SV=1 | A1KKZ3_MYCBP | 0 | + | 0 | 0 | 0 | 0 |
| 1. Monooxygenase OS=Mycobacterium fortuitum subsp. fortuitum DSM 46621 GN=MFORT_24947 PE=4 SV=1 | K0UT08_MYCFO | 0 | 0 | + | 0 | 0 | + |
| 1. ATP-dependent Clp protease proteolytic subunit OS=Mycobacterium avium subsp. paratuberculosis S5 GN=clpP PE=3 SV=1 | L7DK09_MYCPC | + | 0 | 0 | 0 | 0 | 0 |
| 1. Cyclopropane-fatty-acyl-phospholipid synthase OS=Mycobacterium fortuitum subsp. fortuitum DSM 46621 GN=MFORT_05368 PE=4 SV=1 | K0V7V6_MYCFO | 0 | 0 | + | 0 | 0 | + |
| 1. Uncharacterized protein OS=Mycobacterium fortuitum subsp. fortuitum DSM 46621 GN=MFORT_02001 PE=4 SV=1 | K0VE63_MYCFO | 0 | 0 | + | 0 | 0 | + |
| 1. Polyribonucleotide nucleotidyltransferase OS=Mycobacterium canettii CIPT 140060008 GN=pnp PE=3 SV=1 | L0PYM0_9MYCO | 0 | + | 0 | 0 | 0 | 0 |
| 1. Putative lipoprotein lprF OS=Mycobacterium bovis (strain BCG / Pasteur 1173P2) GN=lprF PE=4 SV=1 | A1KIF8_MYCBP | 0 | + | 0 | 0 | 0 | 0 |
| 1. Metallopeptidase OS=Mycobacterium fortuitum subsp. fortuitum DSM 46621 GN=MFORT_15262 PE=4 SV=1 | K0VPU0_MYCFO | 0 | 0 | 0 | 0 | 0 | + |
| 1. ESX-1 secretion-associated protein EspA OS=Mycobacterium canettii CIPT 140070017 GN=BN45_100126 PE=4 SV=1 | L0QZY1_9MYCO | 0 | + | 0 | 0 | 0 | 0 |
| 1. Superoxide dismutase OS=Mycobacterium avium (strain 104) GN=MAV_0182 PE=3 SV=1 | A0Q988_MYCA1 | + | 0 | 0 | 0 | 0 | 0 |
| 1. Nucleoside diphosphate kinase regulator OS=Mycobacterium avium (strain 104) GN=MAV_0234 PE=4 SV=1 | A0Q9D9_MYCA1 | + | 0 | 0 | 0 | 0 | 0 |
| 1. Possible oxidoreductase OS=Mycobacterium bovis (strain BCG / Pasteur 1173P2) GN=BCG_0094 PE=4 SV=1 | A1KEN1_MYCBP | 0 | + | 0 | 0 | 0 | 0 |
| 1. Putative pterin-4-alpha-carbinolamine dehydratase OS=Mycobacterium tuberculosis str. Haarlem GN=TBHG_01143 PE=3 SV=1 | A4KG87_MYCTX | 0 | + | 0 | 0 | 0 | 0 |
| 1. Alkyl hydroperoxide reductase OS=Mycobacterium fortuitum subsp. fortuitum DSM 46621 GN=MFORT_22390 PE=4 SV=1 | K0VEF0_MYCFO | 0 | 0 | 0 | 0 | 0 | + |
| 1. Uncharacterized protein OS=Mycobacterium avium subsp. hominissuis TH135 GN=MAH_2567 PE=4 SV=1 | T2GUG0_MYCAV | + | 0 | 0 | 0 | 0 | 0 |
| 1. ErfK/YbiS/YcfS/YnhG family protein OS=Mycobacterium avium (strain 104) GN=MAV_4986 PE=4 SV=1 | A0QMG9_MYCA1 | + | 0 | 0 | 0 | 0 | 0 |
| 1. ATP-dependent Clp protease proteolytic subunit OS=Mycobacterium fortuitum subsp. fortuitum DSM 46621 GN=clpP PE=3 SV=1 | K0V9Z4_MYCFO | 0 | 0 | + | 0 | + | + |
| 1. Triosephosphate isomerase OS=Mycobacterium fortuitum subsp. fortuitum DSM 46621 GN=tpiA PE=3 SV=1 | K0V898_MYCFO | 0 | 0 | + | 0 | 0 | 0 |
| 1. Serine protease htrA OS=Mycobacterium tuberculosis T17 GN=TBJG_01731 PE=4 SV=1 | D5ZEX8_MYCTX | 0 | + | 0 | 0 | 0 | 0 |
| 1. Trypsin OS=Mycobacterium fortuitum subsp. fortuitum DSM 46621 GN=MFORT_21860 PE=4 SV=1 | K0UXD3_MYCFO | 0 | 0 | + | 0 | 0 | 0 |
| 1. Fructose-2,6-bisphosphatase OS=Mycobacterium fortuitum subsp. fortuitum DSM 46621 GN=MFORT_15377 PE=4 SV=1 | K0VEH0_MYCFO | 0 | 0 | + | 0 | + | + |
| 1. Uncharacterized protein OS=Mycobacterium kansasii ATCC 12478 GN=MKAN_10295 PE=4 SV=1 | U5X1U5_MYCKA | 0 | 0 | 0 | + | 0 | 0 |
| 1. Uncharacterized protein OS=Mycobacterium fortuitum subsp. fortuitum DSM 46621 GN=MFORT_22475 PE=4 SV=1 | K0URE6_MYCFO | 0 | 0 | + | 0 | + | 0 |
| 1. Serine esterase cutinase OS=Mycobacterium avium (strain 104) GN=MAV_0369 PE=4 SV=1 | A0Q9R7_MYCA1 | + | 0 | 0 | 0 | 0 | 0 |
| 1. HIT family hydrolase, diadenosine tetraphosphate hydrolase OS=Mycobacterium avium subsp. paratuberculosis S397 GN=MAPs_13800 PE=4 SV=1 | F7PC21_MYCPC | + | 0 | 0 | 0 | 0 | 0 |
| 1. O-methyltransferase OS=Mycobacterium avium 05-4293 GN=O984_01700 PE=4 SV=1 | V7JEA2_MYCAV | + | 0 | 0 | 0 | 0 | 0 |
| 1. Proteasome-associated ATPase OS=Mycobacterium fortuitum subsp. fortuitum DSM 46621 GN=mpa PE=3 SV=1 | K0VLY9_MYCFO | 0 | 0 | + | 0 | 0 | 0 |
| 1. Fatty oxidation protein fadB OS=Mycobacterium tuberculosis C GN=TBCG_00850 PE=3 SV=1 | A2VGF6_MYCTX | 0 | + | 0 | 0 | 0 | 0 |
| 1. Malate synthase G OS=Mycobacterium tuberculosis str. Haarlem/NITR202 GN=glcB PE=3 SV=1 | R4M7L1_MYCTX | 0 | + | 0 | 0 | 0 | 0 |
| 1. 3-ketoacyl-(Acyl-carrier-protein) reductase (Fragment) OS=Mycobacterium fortuitum subsp. fortuitum DSM 46621 GN=fabG PE=3 SV=1 | K0UUR3_MYCFO | 0 | 0 | 0 | 0 | 0 | + |
| 1. Serine hydroxymethyltransferase OS=Mycobacterium tuberculosis (strain ATCC 25177 / H37Ra) GN=glyA1 PE=3 SV=1 | A5U1E0_MYCTA | 0 | + | 0 | 0 | 0 | 0 |
| 1. Uncharacterized protein OS=Mycobacterium tuberculosis (strain ATCC 25177 / H37Ra) GN=MRA_3428A PE=4 SV=1 | A5U862_MYCTA | 0 | + | 0 | 0 | 0 | 0 |
| 1. 6-phosphogluconate dehydrogenase, decarboxylating OS=Mycobacterium fortuitum subsp. fortuitum DSM 46621 GN=MFORT_13435 PE=3 SV=1 | K0V2V2_MYCFO | 0 | 0 | + | 0 | 0 | + |
| 1. Cutinase OS=Mycobacterium fortuitum subsp. fortuitum DSM 46621 GN=MFORT_20540 PE=4 SV=1 | K0UTQ3_MYCFO | 0 | 0 | + | 0 | 0 | 0 |
| 1. Probable serine protease OS=Mycobacterium bovis (strain BCG / Pasteur 1173P2) GN=BCG_1038 PE=4 SV=1 | A1KHB9_MYCBP | 0 | + | 0 | 0 | 0 | 0 |
| 1. Uncharacterized protein, probably involved in trehalose biosynthesis OS=Mycobacterium avium subsp. paratuberculosis S397 GN=MAPs_45790 PE=4 SV=1 | F7P7T7_MYCPC | + | 0 | 0 | 0 | 0 | 0 |
| 1. Cellobiohydrolase A (1,4-beta-cellobiosidase A) OS=Mycobacterium avium subsp. paratuberculosis S397 GN=MAPs_23360 PE=4 SV=1 | F7PER0_MYCPC | + | 0 | 0 | 0 | 0 | 0 |
| 1. Uncharacterized protein OS=Mycobacterium avium (strain 104) GN=MAV_3218 PE=4 SV=1 | A0QHL2_MYCA1 | + | 0 | 0 | 0 | 0 | 0 |
| 1. Lipoprotein, ATP binding protein OS=Mycobacterium vaccae ATCC 25954 GN=MVAC_19346 PE=4 SV=1 | K0UKI4_MYCVA | 0 | 0 | 0 | 0 | 0 | 0 |
| 1. DNA gyrase subunit A OS=Mycobacterium fortuitum subsp. fortuitum DSM 46621 GN=gyrA PE=3 SV=1 | K0UVA8_MYCFO | 0 | 0 | 0 | 0 | 0 | + |
| 1. Arginine biosynthesis bifunctional protein ArgJ OS=Mycobacterium fortuitum subsp. fortuitum DSM 46621 GN=argJ PE=3 SV=1 | K0V230_MYCFO | 0 | 0 | + | 0 | 0 | 0 |
| 1. Probable acetyl-CoA acyltransferase fadA2 OS=Mycobacterium bovis (strain BCG / Pasteur 1173P2) GN=fadA2 PE=3 SV=1 | A1KF64_MYCBP | 0 | + | 0 | 0 | 0 | 0 |
| 1. Putative uncharacterized protein OS=Mycobacterium tuberculosis EAS054 GN=TBGG_03363 PE=4 SV=1 | D5YMQ2_MYCTX | 0 | + | 0 | 0 | 0 | 0 |
| 1. Adenylate kinase OS=Mycobacterium tuberculosis C GN=adk PE=3 SV=1 | A2VG38_MYCTX | 0 | + | 0 | 0 | 0 | 0 |
| 1. Eptc-inducible aldehyde dehydrogenase OS=Mycobacterium avium (strain 104) GN=MAV_4691 PE=3 SV=1 | A0QLN1_MYCA1 | + | 0 | 0 | 0 | 0 | 0 |
| 1. Uncharacterized protein OS=Mycobacterium bovis (strain BCG / Pasteur 1173P2) GN=BCG_1931 PE=4 SV=1 | A1KJV7_MYCBP | 0 | + | 0 | 0 | 0 | 0 |
| 1. Two component transcriptional regulator OS=Mycobacterium fortuitum subsp. fortuitum DSM 46621 GN=MFORT_08835 PE=4 SV=1 | K0V6I9_MYCFO | 0 | 0 | + | 0 | 0 | + |
| 1. D-3-phosphoglycerate dehydrogenase OS=Mycobacterium fortuitum subsp. fortuitum DSM 46621 GN=MFORT_08481 PE=3 SV=1 | K0VAG8_MYCFO | 0 | 0 | 0 | 0 | 0 | + |
| 1. Uncharacterized protein OS=Mycobacterium fortuitum subsp. fortuitum DSM 46621 GN=MFORT_13480 PE=4 SV=1 | K0V3S4_MYCFO | 0 | 0 | + | 0 | 0 | + |
| 1. Uncharacterized protein OS=Mycobacterium kansasii ATCC 12478 GN=MKAN_29175 PE=4 SV=1 | U5X3E8_MYCKA | 0 | 0 | 0 | + | 0 | 0 |
| 1. 50S ribosomal protein L10 OS=Mycobacterium fortuitum subsp. fortuitum DSM 46621 GN=rplJ PE=3 SV=1 | K0V7X9_MYCFO | 0 | 0 | + | 0 | 0 | 0 |
| 1. Transmembrane transport protein MmpL10 OS=Mycobacterium tuberculosis str. Haarlem/NITR202 GN=I917_08410 PE=4 SV=1 | R4LWQ9_MYCTX | 0 | 0 | 0 | 0 | + | 0 |
| 1. Enoyl-CoA hydratase OS=Mycobacterium avium (strain 104) GN=MAV_4534 PE=4 SV=1 | A0QL77_MYCA1 | + | 0 | 0 | 0 | 0 | 0 |
| 1. Periplasmic binding protein OS=Mycobacterium avium subsp. hominissuis TH135 GN=MAH_3333 PE=4 SV=1 | T2GUZ7_MYCAV | + | 0 | 0 | 0 | 0 | 0 |
| 1. Pyruvate dehydrogenase (E2 component) sucB OS=Mycobacterium tuberculosis C GN=TBCG_02162 PE=3 SV=1 | A2VJV6_MYCTX | 0 | + | 0 | 0 | 0 | 0 |
| 1. Uncharacterized protein OS=Mycobacterium bovis (strain BCG / Pasteur 1173P2) GN=BCG_0288c PE=4 SV=1 | A1KF71_MYCBP | 0 | + | 0 | 0 | 0 | 0 |
| 1. Short chain dehydrogenase OS=Mycobacterium avium (strain 104) GN=MAV_0895 PE=3 SV=1 | A0QB71_MYCA1 | + | 0 | 0 | 0 | 0 | 0 |
| 1. Hydrolase OS=Mycobacterium fortuitum subsp. fortuitum DSM 46621 GN=MFORT_10055 PE=4 SV=1 | K0V5Z0_MYCFO | 0 | 0 | + | 0 | 0 | 0 |
| 1. Uncharacterized protein OS=Mycobacterium tuberculosis str. Haarlem/NITR202 GN=I917_10075 PE=4 SV=1 | R4M6G6_MYCTX | 0 | + | 0 | 0 | 0 | 0 |
| 1. Acyl carrier protein OS=Mycobacterium vanbaalenii (strain DSM 7251 / PYR-1) GN=acpP PE=3 SV=1 | A1TBI7_MYCVP | 0 | 0 | 0 | 0 | 0 | + |
| 1. Uncharacterized protein OS=Mycobacterium tuberculosis str. Haarlem/NITR202 GN=I917_10575 PE=4 SV=1 | R4LUQ9_MYCTX | 0 | + | 0 | 0 | 0 | 0 |
| 1. 6-phosphogluconate dehydrogenase, decarboxylating (Fragment) OS=Mycobacterium fortuitum subsp. fortuitum DSM 46621 GN=MFORT_28060 PE=3 SV=1 | K0V658_MYCFO | 0 | 0 | + | 0 | 0 | 0 |
| 1. Polyketide synthase pks13 OS=Mycobacterium bovis (strain BCG / Pasteur 1173P2) GN=pks13 PE=4 SV=1 | A1KQD4_MYCBP | 0 | + | 0 | 0 | 0 | 0 |
| 1. Aconitate hydratase OS=Mycobacterium tuberculosis (strain CCDC5079) GN=CCDC5079_1371 PE=4 SV=1 | F7WJA8_MYCTC | 0 | + | 0 | 0 | 0 | 0 |
| 1. 2,3-bisphosphoglycerate-dependent phosphoglycerate mutase OS=Mycobacterium fortuitum subsp. fortuitum DSM 46621 GN=gpmA PE=3 SV=1 | K0VIB0_MYCFO | 0 | 0 | + | 0 | 0 | 0 |
| 1. Ribonucleoside-diphosphate reductase OS=Mycobacterium fortuitum subsp. fortuitum DSM 46621 GN=MFORT_03506 PE=3 SV=1 | K0VL43_MYCFO | 0 | 0 | 0 | 0 | 0 | + |
| 1. Uncharacterized protein OS=Mycobacterium fortuitum subsp. fortuitum DSM 46621 GN=MFORT_19851 PE=4 SV=1 | K0V753_MYCFO | 0 | 0 | 0 | 0 | 0 | + |
| 1. Uncharacterized protein OS=Mycobacterium fortuitum subsp. fortuitum DSM 46621 GN=MFORT_29254 PE=4 SV=1 | K0ULC0_MYCFO | 0 | 0 | + | 0 | + | 0 |
| 1. Uncharacterized protein OS=Mycobacterium fortuitum subsp. fortuitum DSM 46621 GN=MFORT_24567 PE=4 SV=1 | K0UMJ9_MYCFO | 0 | 0 | + | 0 | 0 | 0 |
| 1. Universal stress protein UspA-like protein OS=Mycobacterium fortuitum subsp. fortuitum DSM 46621 GN=MFORT_24067 PE=4 SV=1 | K0V1J0_MYCFO | 0 | 0 | 0 | 0 | 0 | + |
| 1. Phosphoserine aminotransferase OS=Mycobacterium fortuitum subsp. fortuitum DSM 46621 GN=serC PE=3 SV=1 | K0V2M1_MYCFO | 0 | 0 | 0 | 0 | 0 | + |
| 1. Naphthoate synthase OS=Mycobacterium avium (strain 104) GN=menB PE=4 SV=1 | A0QLD7_MYCA1 | + | 0 | 0 | 0 | 0 | 0 |
| 1. Nitroreductase family protein OS=Mycobacterium avium (strain 104) GN=MAV_4334 PE=4 SV=1 | A0QKN2_MYCA1 | + | 0 | 0 | 0 | 0 | 0 |
| 1. Ribosome-recycling factor OS=Mycobacterium avium subsp. paratuberculosis S397 GN=frr PE=3 SV=1 | F7PA73_MYCPC | + | 0 | 0 | 0 | 0 | 0 |
| 1. Catalase-peroxidase OS=Mycobacterium tuberculosis GN=katG PE=3 SV=1 | M1JUT6_MYCTX | 0 | + | 0 | 0 | 0 | 0 |
| 1. GntR family transcriptional regulator OS=Mycobacterium fortuitum subsp. fortuitum DSM 46621 GN=MFORT_10764 PE=4 SV=1 | K0V4G5_MYCFO | 0 | 0 | + | 0 | 0 | 0 |
| 1. FHA domain-containing protein OS=Mycobacterium fortuitum subsp. fortuitum DSM 46621 GN=MFORT_16749 PE=4 SV=1 | K0VD97_MYCFO | 0 | 0 | + | 0 | 0 | 0 |
| 1. Cyanate hydratase OS=Mycobacterium avium (strain 104) GN=cynS PE=3 SV=1 | CYNS_MYCA1 | + | 0 | 0 | 0 | 0 | 0 |
| 1. Universal stress protein family protein OS=Mycobacterium fortuitum subsp. fortuitum DSM 46621 GN=MFORT_17998 PE=4 SV=1 | K0UZ85_MYCFO | 0 | 0 | 0 | 0 | 0 | + |
| 1. Molybdenum ABC transporter periplasmic molybdate-binding protein OS=Mycobacterium fortuitum subsp. fortuitum DSM 46621 GN=MFORT_19604 PE=4 SV=1 | K0VIY0_MYCFO | 0 | 0 | + | 0 | 0 | 0 |
| 1. Signal-transduction protein OS=Mycobacterium fortuitum subsp. fortuitum DSM 46621 GN=MFORT_23917 PE=4 SV=1 | K0UNL7_MYCFO | 0 | 0 | 0 | 0 | 0 | + |
| 1. Ferredoxin sulfite reductase OS=Mycobacterium fortuitum subsp. fortuitum DSM 46621 GN=MFORT_00485 PE=4 SV=1 | K0VF31_MYCFO | 0 | 0 | 0 | 0 | 0 | + |
| 1. D-alanyl-D-alanine carboxypeptidase/D-alanyl-D-alanine-endopeptidase OS=Mycobacterium avium (strain 104) GN=dacB PE=4 SV=1 | A0QA71_MYCA1 | + | 0 | 0 | 0 | 0 | 0 |
| 1. Possible thioredoxin OS=Mycobacterium bovis (strain BCG / Pasteur 1173P2) GN=BCG_1386 PE=4 SV=1 | A1KIB4_MYCBP | 0 | + | 0 | 0 | 0 | 0 |
| 1. Pyridoxamine 5''-phosphate oxidase OS=Mycobacterium fortuitum subsp. fortuitum DSM 46621 GN=MFORT_13990 PE=4 SV=1 | K0VQG9_MYCFO | 0 | 0 | 0 | 0 | 0 | + |
| 1. Probable cytosol aminopeptidase OS=Mycobacterium avium (strain 104) GN=pepA PE=3 SV=1 | A0QEZ5_MYCA1 | + | 0 | 0 | 0 | 0 | 0 |
| 1. Probable thiosulfate sulfurtransferase sseA OS=Mycobacterium bovis (strain BCG / Pasteur 1173P2) GN=sseA PE=4 SV=1 | A1KNT5_MYCBP | 0 | + | 0 | 0 | 0 | 0 |
| 1. 2-oxoglutarate dehydrogenase, E2 component, dihydrolipoamide succinyltransferase OS=Mycobacterium avium (strain 104) GN=sucB PE=3 SV=1 | A0QEY9_MYCA1 | + | 0 | 0 | 0 | 0 | 0 |
| 1. Alpha-ketoglutarate decarboxylase OS=Mycobacterium fortuitum subsp. fortuitum DSM 46621 GN=kgd PE=4 SV=1 | K0UZY0_MYCFO | 0 | 0 | 0 | 0 | 0 | + |
| 1. Possible conserved transmembrane alanine and glycine rich protein OS=Mycobacterium bovis (strain BCG / Pasteur 1173P2) GN=BCG_2734c PE=4 SV=1 | A1KM59_MYCBP | 0 | + | 0 | 0 | 0 | 0 |
| 1. Glycosyl hydrolase OS=Mycobacterium fortuitum subsp. fortuitum DSM 46621 GN=MFORT_28674 PE=4 SV=1 | K0UFN0_MYCFO | 0 | 0 | + | 0 | + | + |
| 1. Acetyl-CoA acetyltransferase OS=Mycobacterium fortuitum subsp. fortuitum DSM 46621 GN=MFORT_29394 PE=3 SV=1 | K0UL52_MYCFO | 0 | 0 | + | 0 | 0 | 0 |
| 1. Alpha/beta hydrolase fold protein OS=Mycobacterium fortuitum subsp. fortuitum DSM 46621 GN=MFORT_10569 PE=4 SV=1 | K0VSA3_MYCFO | 0 | 0 | + | 0 | 0 | + |
| 1. Ribose-phosphate pyrophosphokinase OS=Mycobacterium fortuitum subsp. fortuitum DSM 46621 GN=prs PE=3 SV=1 | K0V2H2_MYCFO | 0 | 0 | + | 0 | 0 | 0 |
| 1. Probable thiol peroxidase OS=Mycobacterium bovis (strain BCG / Pasteur 1173P2) GN=tpx PE=3 SV=1 | A1KJZ7_MYCBP | 0 | + | 0 | 0 | 0 | 0 |
| 1. Uncharacterized protein OS=Mycobacterium fortuitum subsp. fortuitum DSM 46621 GN=MFORT_14717 PE=4 SV=1 | K0V7E6_MYCFO | 0 | 0 | + | 0 | 0 | 0 |
| 1. Probable fructose-bisphosphate aldolase fba OS=Mycobacterium bovis (strain BCG / Pasteur 1173P2) GN=fba PE=4 SV=1 | A1KFI4_MYCBP | 0 | + | 0 | 0 | 0 | 0 |
| 1. 6-phosphogluconate dehydrogenase, decarboxylating OS=Mycobacterium avium (strain 104) GN=gnd PE=4 SV=1 | A0QC60_MYCA1 | + | 0 | 0 | 0 | 0 | 0 |
| 1. Uncharacterized protein OS=Mycobacterium avium subsp. hominissuis TH135 GN=MAH_0266 PE=3 SV=1 | T2GMN3_MYCAV | + | 0 | 0 | 0 | 0 | 0 |
| 1. Chaperone protein ClpB OS=Mycobacterium paratuberculosis (strain ATCC BAA-968 / K-10) GN=clpB PE=3 SV=1 | CLPB_MYCPA | + | 0 | 0 | 0 | 0 | 0 |
| 1. Meromycolate extension acyl carrier protein OS=Mycobacterium aurum GN=acpM PE=3 SV=2 | ACPM_MYCAU | 0 | 0 | 0 | 0 | 0 | + |
| 1. D-alanyl-D-alanine carboxypeptidase/D-alanyl-D-alanine-endopeptidase OS=Mycobacterium fortuitum subsp. fortuitum DSM 46621 GN=MFORT_23852 PE=4 SV=1 | K0V1M2_MYCFO | 0 | 0 | + | 0 | 0 | 0 |
| 1. Uncharacterized protein OS=Mycobacterium kansasii ATCC 12478 GN=MKAN_04170 PE=3 SV=1 | U5WNW8_MYCKA | 0 | 0 | 0 | + | 0 | 0 |
| 1. Uncharacterized protein OS=Mycobacterium fortuitum subsp. fortuitum DSM 46621 GN=MFORT_13870 PE=4 SV=1 | K0VFF0_MYCFO | 0 | 0 | + | 0 | + | + |
| 1. Anti-sigma factor antagonist OS=Mycobacterium fortuitum subsp. fortuitum DSM 46621 GN=MFORT_23872 PE=3 SV=1 | K0UNP9_MYCFO | 0 | 0 | + | 0 | 0 | 0 |
| 1. Cytoplasmic peptidase OS=Mycobacterium tuberculosis (strain ATCC 35801 / TMC 107 / Erdman) GN=pepQ PE=4 SV=1 | H8EU58_MYCTE | 0 | + | 0 | 0 | 0 | 0 |
| 1. NAD-dependent malic enzyme (Fragment) OS=Mycobacterium fortuitum subsp. fortuitum DSM 46621 GN=MFORT_31371 PE=3 SV=1 | K0UBD0_MYCFO | 0 | 0 | + | 0 | + | 0 |
| 1. R4V0A1\|R4V0A1_MYCAB-DECOY |  | 0 | 0 | 0 | 0 | + | 0 |
| 1. Proteasome subunit beta OS=Mycobacterium avium subsp. avium 11-4751 GN=O973_11070 PE=3 SV=1 | V7LGT8_MYCAV | + | 0 | 0 | 0 | 0 | 0 |
| 1. Uncharacterized protein OS=Mycobacterium avium (strain 104) GN=MAV_2810 PE=4 SV=1 | A0QGF9_MYCA1 | + | 0 | 0 | 0 | 0 | 0 |
| 1. B1MMN6\|B1MMN6_MYCA9-DECOY | B1MMN6\|DECOY | 0 | 0 | 0 | 0 | + | 0 |
| 1. Polyketide-type polyunsaturated fatty acid synthase PfaA OS=Mycobacterium rhodesiae JS60 GN=MycrhDRAFT_5147 PE=4 SV=1 | G4I5E7_MYCRH | 0 | 0 | 0 | 0 | + | 0 |
| 1. Uncharacterized protein OS=Mycobacterium fortuitum subsp. fortuitum DSM 46621 GN=MFORT_06631 PE=4 SV=1 | K0V7T7_MYCFO | 0 | 0 | + | 0 | 0 | + |
| 1. Succinate dehydrogenase flavoprotein subunit OS=Mycobacterium fortuitum subsp. fortuitum DSM 46621 GN=sdhA PE=4 SV=1 | K0V2T1_MYCFO | 0 | 0 | 0 | 0 | 0 | + |
| 1. Methylmalonate-semialdehyde dehydrogenase OS=Mycobacterium fortuitum subsp. fortuitum DSM 46621 GN=MFORT_30399 PE=3 SV=1 | K0UJ90_MYCFO | 0 | 0 | + | 0 | 0 | 0 |
| 1. I6ZHP2\|I6ZHP2_MYCAB-DECOY | I6ZHP2\|I6ZHP2_ | 0 | 0 | 0 | 0 | + | 0 |
| 1. Probable acyl-CoA dehydrogenase fadE35 OS=Mycobacterium bovis (strain BCG / Pasteur 1173P2) GN=fadE35 PE=3 SV=1 | A1KQD1_MYCBP | 0 | + | 0 | 0 | 0 | 0 |
| 1. Uncharacterized protein OS=Mycobacterium avium (strain 104) GN=MAV_1706 PE=4 SV=1 | A0QDE7_MYCA1 | + | 0 | 0 | 0 | 0 | 0 |
| 1. Aspartate transaminase OS=Mycobacterium avium (strain 104) GN=MAV_0381 PE=4 SV=1 | A0Q9S8_MYCA1 | + | 0 | 0 | 0 | 0 | 0 |
| 1. Bifunctional acetyl-/propionyl-coenzyme A carboxylase subunit alpha accA3 OS=Mycobacterium africanum K85 GN=TBOG_03849 PE=4 SV=1 | D6FRI7_9MYCO | 0 | + | 0 | 0 | 0 | 0 |
| 1. Uncharacterized protein OS=Mycobacterium kansasii ATCC 12478 GN=MKAN_17890 PE=4 SV=1 | U5WRT3_MYCKA | 0 | 0 | 0 | 0 | 0 | 0 |
| 1. Cutinase OS=Mycobacterium kansasii ATCC 12478 GN=MKAN_20050 PE=4 SV=1 | U5WWB6_MYCKA | 0 | 0 | 0 | 0 | 0 | 0 |
| 1. Probable enoyl-CoA hydratase echa16 OS=Mycobacterium bovis (strain BCG / Pasteur 1173P2) GN=echA16 PE=4 SV=1 | A1KMH4_MYCBP | 0 | + | 0 | 0 | 0 | 0 |
| 1. Dihydrolipoamide acetyltransferase (Fragment) OS=Mycobacterium fortuitum subsp. fortuitum DSM 46621 GN=MFORT_27485 PE=3 SV=1 | K0UNN7_MYCFO | 0 | 0 | 0 | 0 | 0 | + |
| 1. Uncharacterized protein OS=Mycobacterium kansasii ATCC 12478 GN=MKAN_08035 PE=4 SV=1 | U5WYC5_MYCKA | 0 | 0 | 0 | 0 | 0 | 0 |
| 1. Serine esterase, cutinase OS=Mycobacterium fortuitum subsp. fortuitum DSM 46621 GN=MFORT_19032 PE=4 SV=1 | K0V8N7_MYCFO | 0 | 0 | + | 0 | + | 0 |
| 1. Pyruvate kinase OS=Mycobacterium fortuitum subsp. fortuitum DSM 46621 GN=MFORT_26049 PE=3 SV=1 | K0V8Y4_MYCFO | 0 | 0 | 0 | 0 | 0 | + |
| 1. Uncharacterized protein OS=Mycobacterium fortuitum subsp. fortuitum DSM 46621 GN=MFORT_20990 PE=4 SV=1 | K0UYS8_MYCFO | 0 | 0 | + | 0 | 0 | 0 |
| 1. Methyltransferase OS=Mycobacterium fortuitum subsp. fortuitum DSM 46621 GN=MFORT_09905 PE=3 SV=1 | K0V5W4_MYCFO | 0 | 0 | 0 | 0 | 0 | + |
| 1. ATP synthase subunit alpha OS=Mycobacterium tuberculosis SUMu004 GN=atpA PE=3 SV=1 | E2TXC6_MYCTX | 0 | + | 0 | 0 | 0 | 0 |
| 1. Glycerophosphoryl diester phosphodiesterase family protein OS=Mycobacterium avium (strain 104) GN=MAV_0576 PE=4 SV=1 | A0QAB7_MYCA1 | + | 0 | 0 | 0 | 0 | 0 |
| 1. R3H domain-containing protein OS=Mycobacterium avium (strain 104) GN=MAV_5309 PE=4 SV=1 | A0QND1_MYCA1 | + | 0 | 0 | 0 | 0 | 0 |
| 1. Uncharacterized protein OS=Mycobacterium bovis (strain BCG / Pasteur 1173P2) GN=BCG_0595c PE=4 SV=1 | A1KG27_MYCBP | 0 | + | 0 | 0 | 0 | 0 |
| 1. Uncharacterized protein OS=Mycobacterium bovis (strain BCG / Pasteur 1173P2) GN=BCG_1771c PE=4 SV=1 | A1KJE9_MYCBP | 0 | + | 0 | 0 | 0 | 0 |
| 1. Probable NADP-dependent alcohol dehydrogenase adhC OS=Mycobacterium bovis (strain BCG / Pasteur 1173P2) GN=adhC PE=3 SV=1 | A1KN42_MYCBP | 0 | + | 0 | 0 | 0 | 0 |
| 1. Methyltransferase, putative, TIGR00027 family OS=Mycobacterium avium subsp. paratuberculosis S397 GN=MAPs_01730 PE=3 SV=1 | F7P8M6_MYCPC | + | 0 | 0 | 0 | 0 | 0 |
| 1. Acetyl-CoA acetyltransferase OS=Mycobacterium avium subsp. silvaticum ATCC 49884 GN=P863_08545 PE=3 SV=1 | V7KNS5_MYCAV | + | 0 | 0 | 0 | 0 | 0 |
| 1. Uncharacterized protein OS=Mycobacterium kansasii ATCC 12478 GN=MKAN_21780 PE=4 SV=1 | U5WUN6_MYCKA | 0 | 0 | 0 | 0 | 0 | 0 |
| 1. Uncharacterized protein OS=Mycobacterium kansasii ATCC 12478 GN=MKAN_25885 PE=4 SV=1 | U5WVT2_MYCKA | 0 | 0 | 0 | 0 | 0 | 0 |
| 1. D-alanyl-D-alanine carboxypeptidase OS=Mycobacterium fortuitum subsp. fortuitum DSM 46621 GN=MFORT_13560 PE=3 SV=1 | K0V3L8_MYCFO | 0 | 0 | + | 0 | 0 | 0 |
| 1. Endonuclease IV OS=Mycobacterium avium subsp. hominissuis TH135 GN=MAH_3909 PE=3 SV=1 | T2GWR1_MYCAV | + | 0 | 0 | 0 | 0 | 0 |
| 1. MmpL protein OS=Mycobacterium smegmatis (strain ATCC 700084 / mc(2)155) GN=MSMEG_4741 PE=4 SV=1 | A0R1G2_MYCS2 | 0 | 0 | 0 | 0 | + | 0 |
| 1. Trigger factor OS=Mycobacterium canettii CIPT 140070008 GN=tig PE=3 SV=1 | L0Q9X7_9MYCO | 0 | + | 0 | 0 | 0 | 0 |
| 1. I4BR24\|I4BR24_MYCCN-DECOY |  | 0 | 0 | 0 | 0 | + | 0 |
| 1. Enoyl-CoA hydratase echA21 OS=Mycobacterium tuberculosis SUMu012 GN=TMLG_00265 PE=4 SV=1 | E2WNJ1_MYCTX | 0 | + | 0 | 0 | 0 | 0 |
| 1. Acyl carrier protein OS=Mycobacterium vaccae ATCC 25954 GN=acpP PE=3 SV=1 | K0UD35_MYCVA | + | 0 | 0 | 0 | 0 | 0 |
| 1. Outer membrane protein OmpA OS=Mycobacterium fortuitum subsp. fortuitum DSM 46621 GN=MFORT_17241 PE=3 SV=1 | K0VN20_MYCFO | 0 | 0 | + | 0 | 0 | 0 |
| 1. Uncharacterized protein OS=Mycobacterium fortuitum subsp. fortuitum DSM 46621 GN=MFORT_11006 PE=4 SV=1 | K0V4C0_MYCFO | 0 | 0 | + | 0 | 0 | 0 |
| 1. Cystathionine gamma-synthase OS=Mycobacterium fortuitum subsp. fortuitum DSM 46621 GN=MFORT_25604 PE=3 SV=1 | K0UYN9_MYCFO | 0 | 0 | + | 0 | 0 | 0 |
| 1. Uncharacterized protein OS=Mycobacterium bovis BCG str. Korea 1168P GN=K60_032490 PE=4 SV=1 | M1IP81_MYCBI | 0 | + | 0 | 0 | 0 | 0 |
| 1. Uncharacterized protein OS=Mycobacterium tuberculosis str. Haarlem/NITR202 GN=I917_27180 PE=4 SV=1 | R4MBZ4_MYCTX | 0 | + | 0 | 0 | 0 | 0 |
| 1. Pyruvate synthase OS=Mycobacterium fortuitum subsp. fortuitum DSM 46621 GN=MFORT_23647 PE=4 SV=1 | K0UP66_MYCFO | 0 | 0 | 0 | 0 | 0 | + |
| 1. Phosphoserine aminotransferase OS=Mycobacterium avium subsp. paratuberculosis S397 GN=serC PE=3 SV=1 | F7P437_MYCPC | + | 0 | 0 | 0 | 0 | 0 |
| 1. NAD(P)H nitroreductase OS=Mycobacterium avium 05-4293 GN=O984_12220 PE=4 SV=1 | V7J5B3_MYCAV | + | 0 | 0 | 0 | 0 | 0 |
| 1. Hydrolase, peptidase M42 family protein OS=Mycobacterium avium (strain 104) GN=MAV_2729 PE=4 SV=1 | A0QG80_MYCA1 | + | 0 | 0 | 0 | 0 | 0 |
| 1. Uncharacterized protein OS=Mycobacterium bovis (strain BCG / Pasteur 1173P2) GN=BCG_3070c PE=4 SV=1 | A1KN43_MYCBP | 0 | + | 0 | 0 | 0 | 0 |
| 1. PE family protein (Fragment) OS=Mycobacterium avium subsp. paratuberculosis S397 GN=MAPs_38890 PE=4 SV=1 | F7P5V6_MYCPC | + | 0 | 0 | 0 | 0 | 0 |
| 1. Ribose-5-phosphate isomerase B OS=Mycobacterium fortuitum subsp. fortuitum DSM 46621 GN=MFORT_02248 PE=4 SV=1 | K0VA03_MYCFO | 0 | 0 | + | 0 | 0 | 0 |
| 1. Gamma-glutamyl phosphate reductase OS=Mycobacterium canettii CIPT 140070017 GN=proA PE=3 SV=1 | L0QY66_9MYCO | 0 | + | 0 | 0 | 0 | 0 |
| 1. Uncharacterized protein OS=Mycobacterium bovis (strain BCG / Pasteur 1173P2) GN=BCG_2051 PE=4 SV=1 | A1KK78_MYCBP | 0 | + | 0 | 0 | 0 | 0 |
| 1. Pyruvate kinase OS=Mycobacterium rhodesiae JS60 GN=MycrhDRAFT_4014 PE=4 SV=1 | G4I2V3_MYCRH | 0 | 0 | 0 | 0 | + | 0 |
| 1. Uncharacterized protein (Fragment) OS=Mycobacterium fortuitum subsp. fortuitum DSM 46621 GN=MFORT_22045 PE=4 SV=1 | K0URT2_MYCFO | 0 | 0 | + | 0 | 0 | 0 |
| 1. Uncharacterized protein OS=Mycobacterium canettii CIPT 140060008 GN=cfp PE=4 SV=1 | L0PUB7_9MYCO | 0 | + | 0 | 0 | 0 | 0 |

| 1. **6 kDa early secretory antigenic target esxA (Esat-6) OS=Mycobacterium tuberculosis C GN=TBCG_03802 PE=4 SV=1** | A2VMQ0_MYCTX | 0 | **+** | 0 | + | 0 | 0 |
| --- | --- | --- | --- | --- | --- | --- | --- |
| 1. **10 kDa chaperonin OS=Mycobacterium tuberculosis C GN=groS PE=3 SV=1** | **A2VPK5_MYCTX** | **+** | **+** | + | + | 0 | + |
| 1. Major secreted immunogenic protein mpb70 OS=Mycobacterium bovis (strain BCG / Pasteur 1173P2) GN=mpb70 PE=4 SV=1 | A1KMM0_MYCBP | 0 | **+** | 0 | 0 | 0 | 0 |
| 1. Bacterioferritin OS=Mycobacterium avium 05-4293 GN=O984_13350 PE=3 SV=1 | V7J3W1_MYCAV | + | 0 | + | 0 | 0 | + |
| 1. 10 kDa culture filtrate antigen esxB (Cfp10) OS=Mycobacterium tuberculosis C GN=TBCG_03801 PE=4 SV=1 | A2VMP9_MYCTX | 0 | **+** | 0 | 0 | + | 0 |
| 1. Esat6 OS=Mycobacterium riyadhense GN=esat6 PE=4 SV=2 | B2CX99_9MYCO | 0 | + | 0 | 0 | 0 | 0 |
| 1. 6**0 kDa chaperonin OS=Mycobacterium tuberculosis str. Haarlem/NITR202 GN=groEL PE=3 SV=1** | R4LUN2_MYCTX | 0 | + | + | + | + | + |
| 1. Antigen 85-B OS=Mycobacterium avium (strain 104) GN=MAV_2816 PE=4 SV=1 | A0QGG5_MYCA1 | + | 0 | 0 | 0 | 0 | 0 |
| 1. Uncharacterized protein OS=Mycobacterium avium (strain 104) GN=MAV_4695 PE=4 SV=1 | A0QLN4_MYCA1 | + | 0 | 0 | 0 | 0 | 0 |
| 1. P**utative ESAT-6 like protein 5 OS=Mycobacterium bovis (strain BCG / Pasteur 1173P2) GN=esxN PE=4 SV=1** | A1KJK3_MYCBP | 0 | + | 0 | + | 0 | 0 |
| 1. Alanine and proline rich secreted protein apa OS=Mycobacterium avium subsp. hominissuis TH135 GN=MAH_2430 PE=4 SV=1 | T2GV03_MYCAV | + | 0 | 0 | 0 | 0 | 0 |
| 1. Cell surface lipoprotein mpt83 (Lipoprotein P23) OS=Mycobacterium tuberculosis C GN=TBCG_02811 PE=4 SV=1 | A2VLK6_MYCTX | 0 | **+** | 0 | 0 | 0 | 0 |
| 1. Antigen 85-C OS=Mycobacterium fortuitum subsp. fortuitum DSM 46621 GN=MFORT_28224 PE=4 SV=1 | K0UMT4_MYCFO | 0 | 0 | + | 0 | + | + |
| 1. 5**0S ribosomal protein L7/L12 OS=Mycobacterium parascrofulaceum ATCC BAA-614 GN=rplL PE=3 SV=1** | D5PC72_9MYCO | + | + | 0 | 0 | + | 0 |
| 1. Uncharacterized protein OS=Mycobacterium avium subsp. avium 10-9275 GN=O972_23545 PE=4 SV=1 | V7KKI2_MYCAV | + | 0 | 0 | 0 | 0 | 0 |
| 1. Uncharacterized protein OS=Mycobacterium avium (strain 104) GN=MAV_3362 PE=4 SV=1 | A0QI06_MYCA1 | + | 0 | 0 | 0 | 0 | 0 |
| 1. Acyl carrier protein OS=Mycobacterium avium (strain 104) GN=acpP PE=3 SV=1 | A0QER4_MYCA1 | + | 0 | 0 | + | + | + |
| 1. P40 protein OS=Mycobacterium avium PE=4 SV=1 | Q9AIQ0_MYCAV | + | 0 | 0 | 0 | 0 | 0 |
| 1. **Elongation factor Tu OS=Mycobacterium fortuitum subsp. fortuitum DSM 46621 GN=tuf PE=3 SV=1** | K0VK30_MYCFO | + | + | + | + | + | + |
| 1. Putative esat-6 like protein OS=Mycobacterium tuberculosis (strain ATCC 25177 / H37Ra) GN=MRA_2375 PE=4 SV=1 | A5U544_MYCTA | 0 | + | 0 | 0 | 0 | 0 |
| 1. Uncharacterized protein OS=Mycobacterium fortuitum subsp. fortuitum DSM 46621 GN=MFORT_04593 PE=4 SV=1 | K0VKN4_MYCFO | 0 | 0 | + | 0 | + | + |
| 1. Serine protease OS=Mycobacterium avium subsp. silvaticum ATCC 49884 GN=P863_01225 PE=4 SV=1 | V7KYG3_MYCAV | + | 0 | 0 | 0 | 0 | 0 |
| 1. **DivIVA domain containing protein OS=Mycobacterium avium subsp. paratuberculosis S397 GN=MAPs_25430 PE=3 SV=1** | F7P246_MYCPC | + | + | + | + | 0 | 0 |
| 1. **Chaperone protein DnaK OS=Mycobacterium tuberculosis C GN=dnaK PE=3 SV=1** | A2VF50_MYCTX | + | + | + | + | + | + |
| 1. Antigen 85-A (Mycolyl transferase) (Fragment) OS=Mycobacterium paratuberculosis GN=fbpa PE=4 SV=1 | Q70E87_MYCPC | + | 0 | 0 | 0 | 0 | 0 |
| 1. 14 kDa antigen OS=Mycobacterium bovis (strain ATCC BAA-935 / AF2122/97) GN=hspX PE=3 SV=2 | 14KD_MYCBO | 0 | + | 0 | 0 | 0 | 0 |
| 1. Esat-6 like protein esxL (Esat-6 like protein 4) OS=Mycobacterium tuberculosis C GN=TBCG_01180 PE=4 SV=1 | A2VHA0_MYCTX | 0 | + | 0 | 0 | 0 | 0 |
| 1. Protease OS=Mycobacterium avium (strain 104) GN=MAV_1096 PE=4 SV=1 | A0QBR1_MYCA1 | + | 0 | 0 | 0 | 0 | 0 |
| 1. 10 kDa culture filtrate antigen EsxB_1 OS=Mycobacterium marinum (strain ATCC BAA-535 / M) GN=esxB_1 PE=4 SV=1 | B2HJI8_MYCMM | 0 | + | 0 | 0 | 0 | 0 |
| 1. Uncharacterized protein OS=Mycobacterium fortuitum subsp. fortuitum DSM 46621 GN=MFORT_07456 PE=4 SV=1 | K0V7A7_MYCFO | 0 | 0 | + | 0 | + | + |
| 1. Immunogenic protein MPT64 OS=Mycobacterium avium (strain 104) GN=MAV_4130 PE=4 SV=1 | A0QK41_MYCA1 | **+** | 0 | 0 | 0 | 0 | 0 |
| 1. A**conitate hydratase OS=Mycobacterium avium subsp. silvaticum ATCC 49884 GN=P863_14265 PE=4 SV=1** | V7KF43_MYCAV | + | + | 0 | 0 | + | + |
| 1. Glutamate binding protein OS=Mycobacterium fortuitum subsp. fortuitum DSM 46621 GN=MFORT_10289 PE=3 SV=1 | K0V4Q6_MYCFO | 0 | 0 | + | 0 | + | + |
| 1. Immunogenic protein mpt64 OS=Mycobacterium tuberculosis C GN=TBCG_01928 PE=4 SV=1 | A2VJ88_MYCTX | 0 | **+** | 0 | 0 | 0 | 0 |
| 1. Antigen 85-A OS=Mycobacterium fortuitum subsp. fortuitum DSM 46621 GN=MFORT_11571 PE=4 SV=1 | K0V3Z5_MYCFO | 0 | 0 | + | 0 | + | + |
| 1. Immunogenic protein MPT63 (Fragment) OS=Mycobacterium fortuitum subsp. fortuitum DSM 46621 GN=MFORT_21420 PE=4 SV=1 | K0US52_MYCFO | 0 | 0 | **+** | 0 | + | **+** |
| 1. Bacterioferritin OS=Mycobacterium fortuitum subsp. fortuitum DSM 46621 GN=MFORT_04403 PE=3 SV=1 | K0VCV8_MYCFO | 0 | 0 | **+** | 0 | + | **+** |
| 1. Probable cutinase Cut3 OS=Mycobacterium avium (strain 104) GN=MAV_4283 PE=4 SV=1 | A0QKI4_MYCA1 | + | 0 | 0 | 0 | 0 | 0 |
| 1. Alanine and proline rich secreted protein apa OS=Mycobacterium tuberculosis C GN=TBCG_01812 PE=4 SV=1 | A2VIX6_MYCTX | 0 | + | 0 | 0 | 0 | 0 |
| 1. E**longation factor Tu OS=Mycobacterium avium (strain 104) GN=tuf PE=3 SV=1** | EFTU_MYCA1 | + | + | 0 | 0 | + | + |
| 1. 60 kDa chaperonin 2 OS=Mycobacterium avium (strain 104) GN=groL2 PE=3 SV=1 | CH602_MYCA1 | + | 0 | 0 | 0 | 0 | 0 |
| 1. Uncharacterized protein OS=Mycobacterium bovis (strain BCG / Pasteur 1173P2) GN=cfp17 PE=4 SV=1 | A1KJN9_MYCBP | 0 | + | 0 | 0 | 0 | 0 |
| 1. 35kd antigen OS=Mycobacterium avium subsp. hominissuis TH135 GN=MAH_3039 PE=4 SV=1 | T2GU78_MYCAV | + | 0 | 0 | 0 | 0 | 0 |
| 1. Transaldolase OS=Mycobacterium avium (strain 104) GN=tal PE=3 SV=1 | A0QHX2_MYCA1 | + | 0 | 0 | 0 | 0 | 0 |
| 1. Electron transfer protein, beta subunit OS=Mycobacterium avium (strain 104) GN=MAV_3876 PE=4 SV=1 | A0QJF0_MYCA1 | + | 0 | + | + | 0 | 0 |
| 1. Uncharacterized protein OS=Mycobacterium colombiense CECT 3035 GN=MCOL_V201805 PE=4 SV=1 | J5ESB1_9MYCO | + | 0 | 0 | 0 | 0 | 0 |
| 1. Mannose-binding lectin OS=Mycobacterium fortuitum subsp. fortuitum DSM 46621 GN=MFORT_27326 PE=4 SV=1 | K0UPC2_MYCFO | 0 | 0 | + | 0 | + | + |
| 1. Low molecular weight antigen MTB12 OS=Mycobacterium fortuitum subsp. fortuitum DSM 46621 GN=MFORT_01821 PE=4 SV=1 | K0VWZ9_MYCFO | 0 | 0 | + | 0 | 0 | + |
| 1. **60 kDa chaperonin OS=Mycobacterium tuberculosis str. Haarlem GN=groL PE=3 SV=1** | A4KEC8_MYCTX | + | + | 0 | 0 | + | + |
| 1. Uncharacterized protein (Fragment) OS=Mycobacterium fortuitum subsp. fortuitum DSM 46621 GN=MFORT_08605 PE=4 SV=1 | K0VA79_MYCFO | 0 | 0 | + | 0 | + | + |
| 1. Acyl carrier protein OS=Mycobacterium bovis (strain BCG / Pasteur 1173P2) GN=acpM PE=3 SV=1 | A1KKT7_MYCBP | 0 | + | 0 | 0 | 0 | 0 |
| 1. Uncharacterized protein OS=Mycobacterium avium (strain 104) GN=MAV_2763 PE=4 SV=1 | A0QGB4_MYCA1 | + | 0 | 0 | 0 | 0 | 0 |
| 1. S**ecreted antigen 85-c fbpC (85C) OS=Mycobacterium bovis (strain BCG / Pasteur 1173P2) GN=fbpC PE=4 SV=1** | A1KEV0_MYCBP | + | + | 0 | 0 | 0 | 0 |
| 1. Uncharacterized protein OS=Mycobacterium fortuitum subsp. fortuitum DSM 46621 GN=MFORT_26634 PE=4 SV=1 | K0UX16_MYCFO | 0 | 0 | + | 0 | + | + |
| 1. Signal peptide protein OS=Mycobacterium avium subsp. silvaticum ATCC 49884 GN=P863_23515 PE=4 SV=1 | V7K1R1_MYCAV | + | 0 | 0 | 0 | 0 | 0 |
| 1. Uncharacterized protein OS=Mycobacterium avium subsp. paratuberculosis S397 GN=MAPs_23480 PE=4 SV=1 | F7PES2_MYCPC | + | 0 | 0 | 0 | 0 | 0 |
| 1. Chaperone protein DnaK OS=Mycobacterium avium (strain 104) GN=dnaK PE=3 SV=1 | DNAK_MYCA1 + | + | 0 | + | 0 | 0 | 0 |
| 1. N-acetylmuramoyl-L-alanine amidase OS=Mycobacterium avium subsp. paratuberculosis 10-8425 GN=O976_20900 PE=4 SV=1 | V7NKM8_MYCPC | + | 0 | 0 | 0 | 0 | 0 |
| 1. **I**mmunogenic protein MPT63 (Antigen MPT63/MPB63) (16 kDa immunoprotective extracellular protein) OS=Mycobacterium tuberculosis str. Haarlem/NITR202 GN=I917_13650 PE=4 SV=1 | R4M6J4_MYCTX | 0 | **+** | 0 | 0 | 0 | 0 |
| 1. **Diacylglycerol acyltransferase/mycolyltransferase Ag85B OS=Mycobacterium kansasii GN=fbpB PE=1 SV=1** | A85B_MYCKA | 0 | + | 0 | + | 0 | 0 |
| 1. Uncharacterized protein OS=Mycobacterium avium subsp. paratuberculosis S397 GN=MAPs_28750 PE=4 SV=1 | F7P319_MYCPC | + | 0 | 0 | 0 | 0 | 0 |
| 1. LpqE protein OS=Mycobacterium avium (strain 104) GN=MAV_0569 PE=4 SV=1 | A0QAB1_MYCA1 | + | 0 | 0 | 0 | 0 | 0 |
| 1. ATP synthase subunit alpha OS=Mycobacterium avium (strain 104) GN=atpA PE=3 SV=1 | ATPA_MYCA1 | + | 0 | + | 0 | 0 | + |
| 1. Phosphate-binding protein PstS OS=Mycobacterium fortuitum subsp. fortuitum DSM 46621 GN=MFORT_17441 PE=3 SV=1 | K0UZG6_MYCFO | 0 | 0 | + | 0 | + | + |
| 1. Universal stress protein family protein OS=Mycobacterium fortuitum subsp. fortuitum DSM 46621 GN=MFORT_14210 PE=4 SV=1 | K0V7H4_MYCFO | 0 | 0 | + | 0 | + | + |
| 1. Antigen 85-C OS=Mycobacterium avium (strain 104) GN=MAV_0215 PE=4 SV=1 | A0Q9C1_MYCA1 | + | 0 | 0 | 0 | 0 | 0 |
| 1. **Malate synthase G OS=Mycobacterium avium (strain 104) GN=glcB PE=3 SV=1** | MASZ_MYCA1 | + | + | 0 | 0 | 0 | 0 |
| 1. Fructose-bisphosphate aldolase class-I OS=Mycobacterium avium (strain 104) GN=MAV_5271 PE=4 SV=1 | A0QN95_MYCA1 | + | 0 | + | 0 | 0 | 0 |
| 1. Uncharacterized protein OS=Mycobacterium avium subsp. paratuberculosis S397 GN=MAPs_09420 PE=4 SV=1 | F7PAT6_MYCPC | + | 0 | 0 | 0 | 0 | 0 |
| 1. Hydroxymethylglutaryl-CoA lyase OS=Mycobacterium avium subsp. silvaticum ATCC 49884 GN=P863_03630 PE=4 SV=1 | V7KWT7_MYCAV | + | 0 | 0 | 0 | 0 | 0 |
| 1. Probable thiol peroxidase OS=Mycobacterium avium (strain 104) GN=tpx PE=3 SV=1 | A0QGC1_MYCA1 | + | 0 | 0 | 0 | 0 | 0 |
| 1. Uncharacterized protein OS=Mycobacterium fortuitum subsp. fortuitum DSM 46621 GN=MFORT_04338 PE=4 SV=1 | K0V8M4_MYCFO | 0 | 0 | + | 0 | + | + |
| 1. 6 kDa early secretory antigenic target OS=Mycobacterium marinum MB2 GN=MMMB2_3804 PE=4 SV=1 | S7RG91_MYCMR | 0 | + | 0 | 0 | 0 | 0 |
| 1. 5-methyltetrahydropteroyltriglutamate--homocysteine methyltransferase OS=Mycobacterium tuberculosis str. Haarlem GN=metE PE=3 SV=2 | A4KG62_MYCTX | 0 | + | 0 | 0 | 0 | 0 |
| 1. 10 kDa culture filtrate protein OS=Mycobacterium szulgai GN=cfp-10 PE=4 SV=1 | B5TV80_MYCSZ | 0 | **+** | 0 | 0 | 0 | 0 |
| 1. A**TP synthase subunit beta OS=Mycobacterium fortuitum subsp. fortuitum DSM 46621 GN=atpD PE=3 SV=1** | K0UJQ0_MYCFO | 0 | 0 | + | 0 | + | 0 |
| 1. Malate dehydrogenase OS=Mycobacterium avium subsp. paratuberculosis S397 GN=mdh PE=3 SV=1 | F7PBF5_MYCPC | + | 0 | 0 | 0 | 0 | 0 |
| 1. Uncharacterized protein OS=Mycobacterium avium (strain 104) GN=MAV_0628 PE=4 SV=1 | A0QAH0_MYCA1 | + | 0 | 0 | 0 | 0 | 0 |
| 1. Electron transfer flavoprotein, alpha subunit OS=Mycobacterium avium subsp. paratuberculosis S397 GN=MAPs_06000 PE=4 SV=1 | F7P9V0_MYCPC | + | 0 | 0 | + | 0 | 0 |
| 1. Uncharacterized protein OS=Mycobacterium avium (strain 104) GN=MAV_0239 PE=4 SV=1 | A0Q9E4_MYCA1 | + | 0 | 0 | 0 | 0 | 0 |
| 1. Uncharacterized protein OS=Mycobacterium avium 05-4293 GN=O984_06270 PE=4 SV=1 | V7J9T0_MYCAV | + | 0 | 0 | 0 | 0 | 0 |
| 1. 60 kDa chaperonin OS=Mycobacterium avium subsp. silvaticum ATCC 49884 GN=groEL PE=3 SV=1 | V7JZC8_MYCAV | + | 0 | 0 | 0 | 0 | 0 |
| 1. Uncharacterized protein OS=Mycobacterium avium subsp. paratuberculosis S397 GN=MAPs_08920 PE=4 SV=1 | F7PAP0_MYCPC | + | 0 | 0 | 0 | 0 | 0 |
| 1. Putative acetyltransferase OS=Mycobacterium avium subsp. paratuberculosis S397 GN=MAPs_11260 PE=4 SV=1 | F7PBB8_MYCPC | + | 0 | 0 | 0 | 0 | 0 |
| 1. Peptidyl-prolyl cis-trans isomerase OS=Mycobacterium avium subsp. paratuberculosis S397 GN=MAPs_23220 PE=4 SV=1 | F7PEP6_MYCPC | + | 0 | 0 | 0 | 0 | 0 |
| 1. Integration host factor OS=Mycobacterium smegmatis (strain ATCC 700084 / mc(2)155) GN=mihF PE=4 SV=1 | A0QWS8_MYCS2 | + | 0 | + | 0 | + | + |
| 1. **DNA-directed RNA polymerase subunit alpha OS=Mycobacterium smegmatis JS623 GN=rpoA PE=3 SV=1** | L0IRX4_MYCSM | + | 0 | 0 | + | 0 | + |
| 1. Transglycosylase OS=Mycobacterium avium (strain 104) GN=MAV_0446 PE=4 SV=1 | A0Q9Z0_MYCA1 | + | 0 | 0 | 0 | 0 | 0 |
| 1. Uncharacterized protein OS=Mycobacterium fortuitum subsp. fortuitum DSM 46621 GN=MFORT_27321 PE=4 SV=1 | K0V769_MYCFO | 0 | 0 | + | 0 | + | + |
| 1. Serine esterase, cutinase OS=Mycobacterium fortuitum subsp. fortuitum DSM 46621 GN=MFORT_00909 PE=4 SV=1 | K0VAQ7_MYCFO | 0 | 0 | + | 0 | + | + |
| 1. Phosphate-binding protein PstS OS=Mycobacterium avium subsp. paratuberculosis S397 GN=MAPs_31960 PE=3 SV=1 | F7P3Y4_MYCPC | + | 0 | 0 | 0 | 0 | 0 |
| 1. Carbohydrate degrading enzyme OS=Mycobacterium avium (strain 104) GN=MAV_1218 PE=4 SV=1 | A0QC26_MYCA1 | + | 0 | 0 | 0 | 0 | 0 |
| 1. Elongation factor Ts OS=Mycobacterium fortuitum subsp. fortuitum DSM 46621 GN=tsf PE=3 SV=1 | K0UW15_MYCFO | 0 | 0 | + | 0 | + | + |
| 1. Fasciclin domain-containing protein OS=Mycobacterium fortuitum subsp. fortuitum DSM 46621 GN=MFORT_25382 PE=4 SV=1 | K0UME3_MYCFO | 0 | 0 | + | 0 | + | + |
| 1. Catalase-peroxidase OS=Mycobacterium avium subsp. paratuberculosis S397 GN=katG PE=3 SV=1 | F7PEL7_MYCPC | + | 0 | 0 | 0 | 0 | 0 |
| 1. **Diacylglycerol acyltransferase/mycolyltransferase Ag85B OS=Mycobacterium smegmatis (strain ATCC 700084 / mc(2)155) GN=fbpB PE=2 SV=1** | A85B_MYCS2 | + | + | 0 | 0 | 0 | 0 |
| 1. Peroxisomal multifunctional enzyme type 2 OS=Mycobacterium avium (strain 104) GN=MAV_5146 PE=3 SV=1 | A0QMX5_MYCA1 | + | 0 | 0 | 0 | 0 | 0 |
| 1. O-methyltransferase OS=Mycobacterium avium subsp. avium 10-9275 GN=O972_00660 PE=4 SV=1 | V7LLA4_MYCAV | + | 0 | 0 | 0 | 0 | 0 |
| 1. 60 kDa chaperonin OS=Mycobacterium fortuitum subsp. fortuitum DSM 46621 GN=groL PE=3 SV=1 | K0V8R4_MYCFO | 0 | 0 | + | 0 | + | + |
| 1. Uncharacterized protein OS=Mycobacterium avium (strain 104) GN=MAV_4742 PE=4 SV=1 | A0QLT1_MYCA1 | + | 0 | 0 | 0 | 0 | 0 |
| 1. Acetyl/propionyl-CoA carboxylase subunit alpha OS=Mycobacterium fortuitum subsp. fortuitum DSM 46621 GN=MFORT_07781 PE=4 SV=1 | K0VB51_MYCFO | 0 | 0 | + | 0 | + | + |
| 1. Enolase OS=Mycobacterium fortuitum subsp. fortuitum DSM 46621 GN=eno PE=3 SV=1 | K0UMN7_MYCFO | + | 0 | + | 0 | 0 | + |
| 1. Adenylate kinase OS=Mycobacterium avium subsp. paratuberculosis S397 GN=adk PE=3 SV=1 | F7P5P8_MYCPC | + | 0 | 0 | 0 | 0 | 0 |
| 1. Acyl-CoA dehydrogenase family protein OS=Mycobacterium avium (strain 104) GN=MAV_4027 PE=3 SV=1 | A0QJU2_MYCA1 | + | 0 | 0 | 0 | 0 | 0 |
| 1. **Probable glyceraldehyde 3-phosphate dehydrogenase gap OS=Mycobacterium bovis (strain BCG / Pasteur 1173P2) GN=gap PE=3 SV=1** | A1KIM5_MYCBP | + | + | 0 | 0 | 0 | 0 |
| 1. Uncharacterized protein OS=Mycobacterium paratuberculosis (strain ATCC BAA-968 / K-10) GN=MAP_0904 PE=4 SV=1 | I3NIF2_MYCPA | + | 0 | 0 | 0 | 0 | 0 |
| 1. Peptidyl-prolyl cis-trans isomerase OS=Mycobacterium fortuitum subsp. fortuitum DSM 46621 GN=MFORT_16694 PE=3 SV=1 | K0V0B7_MYCFO | 0 | 0 | + | 0 | + | + |
| 1. Trigger factor OS=Mycobacterium avium 05-4293 GN=tig PE=3 SV=1 | V7J4X3_MYCAV | + | 0 | 0 | 0 | 0 | 0 |
| 1. Uncharacterized protein OS=Mycobacterium avium (strain 104) GN=MAV_1314 PE=4 SV=1 | A0QCC1_MYCA1 | + | 0 | 0 | 0 | 0 | 0 |
| 1. Antigen 85-C OS=Mycobacterium fortuitum subsp. fortuitum DSM 46621 GN=MFORT_11566 PE=4 SV=1 | K0V526_MYCFO | 0 | 0 | + | 0 | + | + |
| 1. Uncharacterized protein OS=Mycobacterium bovis (strain BCG / Pasteur 1173P2) GN=BCG_2653c PE=4 SV=1 | A1KLX8_MYCBP | 0 | + | 0 | 0 | 0 | 0 |
| 1. ATP synthase subunit beta OS=Mycobacterium avium subsp. hominissuis 10-5606 GN=atpD PE=3 SV=1 | V7N8Q0_MYCAV | + | 0 | 0 | + | 0 | 0 |
| 1. Porin M1 OS=Mycobacterium fortuitum subsp. fortuitum GN=porM1 PE=4 SV=1 | A1ING8_MYCFO | 0 | 0 | + | 0 | 0 | + |
| 1. Phosphoglycerate kinase OS=Mycobacterium fortuitum subsp. fortuitum DSM 46621 GN=pgk PE=3 SV=1 | K0V8M9_MYCFO | 0 | 0 | + | 0 | + | + |
| 1. Bacteriocin OS=Mycobacterium avium subsp. silvaticum ATCC 49884 GN=P863_17350 PE=4 SV=1 | V7KBQ7_MYCAV | + | 0 | 0 | 0 | 0 | 0 |
| 1. Uncharacterized protein OS=Mycobacterium fortuitum subsp. fortuitum DSM 46621 GN=MFORT_20470 PE=4 SV=1 | K0UZS8_MYCFO | 0 | 0 | + | 0 | + | + |
| 1. Uncharacterized protein OS=Mycobacterium fortuitum subsp. fortuitum DSM 46621 GN=MFORT_03321 PE=4 SV=1 | K0V9J9_MYCFO | 0 | 0 | + | 0 | + | + |
| 1. Uncharacterized protein OS=Mycobacterium avium (strain 104) GN=MAV_2808 PE=4 SV=1 | A0QGF7_MYCA1 | + | 0 | 0 | 0 | 0 | 0 |
| 1. Universal stress protein UspA-like protein OS=Mycobacterium avium subsp. hominissuis TH135 GN=MAH_2160 PE=4 SV=1 | T2GU96_MYCAV | + | 0 | 0 | 0 | 0 | 0 |
| 1. Single-stranded DNA-binding protein OS=Mycobacterium avium subsp. hominissuis TH135 GN=ssb PE=4 SV=1 | T2GMI5_MYCAV | + | 0 | + | 0 | + | 0 |
| 1. Uncharacterized protein OS=Mycobacterium fortuitum subsp. fortuitum DSM 46621 GN=MFORT_27840 PE=4 SV=1 | K0UND9_MYCFO | 0 | 0 | + | 0 | 0 | + |
| 1. Uncharacterized protein OS=Mycobacterium avium subsp. silvaticum ATCC 49884 GN=P863_11575 PE=4 SV=1 | V7KKF2_MYCAV | + | 0 | 0 | 0 | 0 | 0 |
| 1. Homogentisate 1,2-dioxygenase OS=Mycobacterium avium subsp. hominissuis TH135 GN=hmgA PE=4 SV=1 | T2GVD0_MYCAV | + | 0 | 0 | 0 | 0 | 0 |
| 1. Adenosylhomocysteinase OS=Mycobacterium tuberculosis EAS054 GN=ahcY PE=3 SV=1 | D5YJL6_MYCTX | 0 | + | 0 | 0 | 0 | 0 |
| 1. 60 kDa chaperonin OS=Mycobacterium fortuitum subsp. fortuitum DSM 46621 GN=groL PE=3 SV=1 | K0VKG7_MYCFO | 0 | 0 | + | 0 | + | + |
| 1. Uncharacterized protein OS=Mycobacterium avium (strain 104) GN=MAV_4234 PE=4 SV=1 | A0QKD7_MYCA1 | + | 0 | 0 | 0 | 0 | 0 |
| 1. Dihydrolipoyl dehydrogenase OS=Mycobacterium fortuitum subsp. fortuitum DSM 46621 GN=MFORT_22940 PE=3 SV=1 | K0UR46_MYCFO | 0 | 0 | + | 0 | 0 | + |
| 1. Uncharacterized protein OS=Mycobacterium bovis (strain BCG / Pasteur 1173P2) GN=BCG_3679c PE=4 SV=1 | A1KPV0_MYCBP | 0 | + | 0 | 0 | 0 | 0 |
| 1. 30S ribosomal protein S6 OS=Mycobacterium fortuitum subsp. fortuitum DSM 46621 GN=rpsF PE=3 SV=1 | K0V3Q6_MYCFO | 0 | 0 | + | 0 | + | + |
| 1. Uncharacterized protein OS=Mycobacterium avium (strain 104) GN=MAV_1445 PE=4 SV=1 | A0QCQ0_MYCA1 | + | 0 | 0 | 0 | 0 | 0 |
| 1. Citrate lyase beta chain citrase beta chain family protein OS=Mycobacterium avium subsp. hominissuis 10-4249 GN=O971_12205 PE=4 SV=1 | V7M5M1_MYCAV | + | 0 | 0 | 0 | 0 | 0 |
| 1. Putative acyl-CoA transferase/carnitine dehydratase OS=Mycobacterium avium subsp. paratuberculosis S397 GN=MAPs_04540 PE=4 SV=1 | F7P9F6_MYCPC | + | 0 | 0 | 0 | 0 | 0 |
| 1. Cutinase OS=Mycobacterium avium subsp. paratuberculosis S397 GN=MAPs_23050 PE=4 SV=1 | F7PEM9_MYCPC | + | 0 | 0 | 0 | 0 | 0 |
| 1. Uncharacterized protein (Fragment) OS=Mycobacterium fortuitum subsp. fortuitum DSM 46621 GN=MFORT_30732 PE=4 SV=1 | K0UQD6_MYCFO | 0 | 0 | + | 0 | + | + |
| 1. Aminopeptidase N OS=Mycobacterium avium (strain 104) GN=pepN PE=4 SV=1 | A0QDE6_MYCA1 | + | 0 | 0 | 0 | 0 | 0 |
| 1. Uncharacterized protein OS=Mycobacterium sp. MOTT36Y GN=W7S_13055 PE=4 SV=1 | I2AE52_9MYCO | + | 0 | 0 | 0 | 0 | 0 |
| 1. Trypsin OS=Mycobacterium avium (strain 104) GN=MAV_1366 PE=4 SV=1 | A0QCH2_MYCA1 | + | 0 | 0 | 0 | 0 | 0 |
| 1. 50S ribosomal protein L29 OS=Mycobacterium avium subsp. paratuberculosis S397 GN=rpmC PE=3 SV=1 | F7P5S9_MYCPC | + | 0 | 0 | 0 | 0 | 0 |
| 1. Uncharacterized protein OS=Mycobacterium bovis (strain BCG / Pasteur 1173P2) GN=wag31 PE=3 SV=1 | A1KKI8_MYCBP | 0 | + | 0 | 0 | 0 | 0 |
| 1. PPE family protein OS=Mycobacterium africanum K85 GN=TBOG_01699 PE=4 SV=1 | D6FR36_9MYCO | 0 | + | 0 | 0 | 0 | 0 |
| 1. Uncharacterized protein OS=Mycobacterium fortuitum subsp. fortuitum DSM 46621 GN=MFORT_18228 PE=4 SV=1 | K0V3D4_MYCFO | 0 | 0 | + | 0 | 0 | 0 |
| 1. Inorganic pyrophosphatase OS=Mycobacterium avium (strain 104) GN=ppa PE=3 SV=1 | A0QA70_MYCA1 | + | 0 | 0 | 0 | 0 | 0 |
| 1. Metallopeptidase, zinc binding OS=Mycobacterium avium (strain 104) GN=MAV_3451 PE=4 SV=1 | A0QI94_MYCA1 | + | 0 | 0 | 0 | 0 | 0 |
| 1. Ribonucleoside-diphosphate reductase subunit beta OS=Mycobacterium fortuitum subsp. fortuitum DSM 46621 GN=nrdF PE=3 SV=1 | K0VW47_MYCFO | 0 | 0 | + | 0 | 0 | + |
| 1. Aconitate hydratase 1 OS=Mycobacterium fortuitum subsp. fortuitum DSM 46621 GN=MFORT_20690 PE=4 SV=1 | K0UZ04_MYCFO | 0 | 0 | + | 0 | + | + |
| 1. Antibiotic biosynthesis monooxygenase domain protein OS=Mycobacterium avium (strain 104) GN=MAV_0562 PE=4 SV=1 | A0QAA4_MYCA1 | + | 0 | 0 | 0 | 0 | 0 |
| 1. Uncharacterized protein OS=Mycobacterium avium subsp. silvaticum ATCC 49884 GN=P863_19285 PE=4 SV=1 | V7K6T2_MYCAV | + | 0 | 0 | 0 | 0 | 0 |
| 1. DNA-directed RNA polymerase subunit beta OS=Mycobacterium fortuitum subsp. fortuitum DSM 46621 GN=rpoB PE=3 SV=1 | K0V8E8_MYCFO | 0 | 0 | + | 0 | + | + |
| 1. Putative BACTERIOFERRITIN BFRB OS=Mycobacterium tuberculosis 7199-99 GN=MT7199_3910 PE=4 SV=1 | L0NZH9_MYCTX | 0 | + | 0 | 0 | 0 | 0 |
| 1. Uncharacterized protein OS=Mycobacterium fortuitum subsp. fortuitum DSM 46621 GN=MFORT_06029 PE=4 SV=1 | K0VBZ4_MYCFO | 0 | 0 | + | 0 | + | + |
| 1. Uncharacterized protein OS=Mycobacterium fortuitum subsp. fortuitum DSM 46621 GN=MFORT_24502 PE=4 SV=1 | K0UTU9_MYCFO | 0 | 0 | 0 | 0 | 0 | + |
| 1. Uncharacterized protein OS=Mycobacterium avium (strain 104) GN=MAV_3557 PE=4 SV=1 | A0QIJ7_MYCA1 | + | 0 | 0 | 0 | 0 | 0 |
| 1. 60 kDa chaperonin OS=Mycobacterium gastri 'Wayne' GN=groEL PE=3 SV=1 | W4HVY4_MYCGS | + | 0 | + | + | 0 | 0 |
| 1. **Iron-dependent repressor IdeR OS=Mycobacterium avium (strain 104) GN=MAV_3604 PE=4 SV=1** | A0QIP3_MYCA1 | + | + | 0 | 0 | 0 | 0 |
| 1. PadR family transcriptional regulator OS=Mycobacterium fortuitum subsp. fortuitum DSM 46621 GN=MFORT_23167 PE=4 SV=1 | K0UVP1_MYCFO | 0 | 0 | 0 | 0 | 0 | + |
| 1. Serine protease pepA OS=Mycobacterium tuberculosis (strain KZN 1435 / MDR) GN=TBMG_00126 PE=4 SV=1 | C6DQZ0_MYCTK | 0 | + | 0 | 0 | 0 | 0 |
| 1. Zn-dependent alcohol dehydrogenase OS=Mycobacterium fortuitum subsp. fortuitum DSM 46621 GN=MFORT_00964 PE=3 SV=1 | K0VMI7_MYCFO | 0 | 0 | 0 | 0 | 0 | + |
| 1. Uncharacterized protein OS=Mycobacterium fortuitum subsp. fortuitum DSM 46621 GN=MFORT_04883 PE=4 SV=1 | K0VCR8_MYCFO | 0 | 0 | + | 0 | + | + |
| 1. Secreted antigen 85-B fbpB (Fibronectin-binding protein B) OS=Mycobacterium tuberculosis C GN=TBCG_01838 PE=4 SV=1 | A2VJ01_MYCTX | 0 | + | 0 | 0 | 0 | 0 |
| 1. Lipoprotein, ATP binding protein OS=Mycobacterium fortuitum subsp. fortuitum DSM 46621 GN=MFORT_02051 PE=4 SV=1 | K0VE69_MYCFO | 0 | 0 | + | 0 | + | + |
| 1. Trypsin OS=Mycobacterium fortuitum subsp. fortuitum DSM 46621 GN=MFORT_10754 PE=4 SV=1 | K0V994_MYCFO | 0 | 0 | + | 0 | + | + |
| 1. Uncharacterized protein OS=Mycobacterium avium (strain 104) GN=MAV_2866 PE=4 SV=1 | A0QGL4_MYCA1 | + | 0 | 0 | 0 | 0 | 0 |
| 1. Polyribonucleotide nucleotidyltransferase OS=Mycobacterium fortuitum subsp. fortuitum DSM 46621 GN=pnp PE=3 SV=1 | K0VIG7_MYCFO | 0 | 0 | + | 0 | 0 | + |
| 1. Phosphoenolpyruvate carboxykinase [GTP] OS=Mycobacterium avium (strain 104) GN=pckG PE=3 SV=1 | A0QME6_MYCA1 | + | 0 | 0 | 0 | 0 | 0 |
| 1. D-3-phosphoglycerate dehydrogenase OS=Mycobacterium avium (strain 104) GN=serA PE=3 SV=1 | A0QJC3_MYCA1 | + | 0 | 0 | 0 | 0 | 0 |
| 1. **30S ribosomal protein S1 OS=Mycobacterium fortuitum subsp. fortuitum DSM 46621 GN=rpsA PE=3 SV=1** | K0V7I2_MYCFO | 0 | + | + | 0 | 0 | + |
| 1. Transketolase OS=Mycobacterium fortuitum subsp. fortuitum DSM 46621 GN=MFORT_26874 PE=3 SV=1 | K0UJQ7_MYCFO | 0 | 0 | + | 0 | 0 | + |
| 1. Uncharacterized protein OS=Mycobacterium fortuitum subsp. fortuitum DSM 46621 GN=MFORT_27790 PE=4 SV=1 | K0UND2_MYCFO | 0 | 0 | + | 0 | + | + |
| 1. Uncharacterized protein OS=Mycobacterium fortuitum subsp. fortuitum DSM 46621 GN=MFORT_26979 PE=4 SV=1 | K0UWR7_MYCFO | 0 | 0 | + | 0 | + | + |
| 1. Uncharacterized protein OS=Mycobacterium fortuitum subsp. fortuitum DSM 46621 GN=MFORT_14737 PE=4 SV=1 | K0VQB7_MYCFO | 0 | 0 | + | 0 | + | + |
| 1. **Forkhead-associated protein OS=Mycobacterium avium (strain 104) GN=MAV_2888 PE=4 SV=1** | A0QGN6_MYCA1 (+20) | + | + | 0 | + | 0 | 0 |
| 1. Iron-regulated conserved protein OS=Mycobacterium ulcerans (strain Agy99) GN=MUL_1619 PE=4 SV=1 | A0PP51_MYCUA (+5) | 0 | + | 0 | 0 | 0 | 0 |
| 1. Lipoprotein Lpps OS=Mycobacterium fortuitum subsp. fortuitum DSM 46621 GN=MFORT_15267 PE=4 SV=1 | K0V6V4_MYCFO | 0 | 0 | + | 0 | + | + |
| 1. Beta-1,3-glucanase OS=Mycobacterium fortuitum subsp. fortuitum DSM 46621 GN=MFORT_02769 PE=4 SV=1 | K0V9N4_MYCFO | 0 | 0 | + | 0 | + | + |
| 1. Conserved protein OS=Mycobacterium marinum (strain ATCC BAA-535 / M) GN=MMAR_4227 PE=4 SV=1 | B2HS57_MYCMM | 0 | + | 0 | 0 | 0 | 0 |
| 1. Glucose-6-phosphate isomerase OS=Mycobacterium avium subsp. avium 10-9275 GN=pgi PE=3 SV=1 | V7KYA2_MYCAV | + | 0 | 0 | 0 | 0 | 0 |
| 1. Acetyl-CoA acetyltransferase OS=Mycobacterium avium (strain 104) GN=MAV_1198 PE=3 SV=1 | A0QC06_MYCA1 | + | 0 | 0 | 0 | 0 | 0 |
| 1. Biotin carboxyl carrier protein OS=Mycobacterium avium subsp. avium 10-9275 GN=O972_01960 PE=4 SV=1 | V7LGX8_MYCAV | + | 0 | 0 | 0 | 0 | 0 |
| 1. Enolase OS=Mycobacterium avium (strain 104) GN=eno PE=3 SV=1 | ENO_MYCA1 | + | 0 | 0 | 0 | 0 | 0 |
| 1. ATP synthase subunit alpha OS=Mycobacterium fortuitum subsp. fortuitum DSM 46621 GN=atpA PE=3 SV=1 | K0V8M3_MYCFO | + | 0 | + | 0 | + | + |
| 1. Acyl-ACP desaturase OS=Mycobacterium fortuitum subsp. fortuitum DSM 46621 GN=MFORT_17978 PE=4 SV=1 | K0UYA2_MYCFO | 0 | 0 | 0 | 0 | 0 | + |
| 1. Thioredoxin OS=Mycobacterium avium (strain 104) GN=trx PE=3 SV=1 | A0QNC4_MYCA1 | + | 0 | 0 | 0 | 0 | 0 |
| 1. Chaperone ClpB OS=Mycobacterium fortuitum subsp. fortuitum DSM 46621 GN=MFORT_28849 PE=3 SV=1 | K0UGP7_MYCFO | 0 | 0 | + | 0 | + | + |
| 1. Phosphotriesterase-like protein OS=Mycobacterium avium (strain 104) GN=MAV_4940 PE=4 SV=1 | A0QMC3_MYCA1 | + | 0 | 0 | 0 | 0 | 0 |
| 1. 6-phosphogluconolactonase OS=Mycobacterium avium subsp. paratuberculosis S397 GN=MAPs_16990 PE=4 SV=1 | F7PCY3_MYCPC | + | 0 | 0 | 0 | 0 | 0 |
| 1. Uncharacterized protein OS=Mycobacterium avium 05-4293 GN=O984_01620 PE=4 SV=1 | V7JCA2_MYCAV | + | 0 | 0 | 0 | 0 | 0 |
| 1. Phosphoglycerate kinase OS=Mycobacterium avium 10-5581 GN=pgk PE=3 SV=1 | V7JEF1_MYCAV | + | 0 | 0 | 0 | 0 | 0 |
| 1. Serine esterase cutinase OS=Mycobacterium avium (strain 104) GN=MAV_2169 PE=4 SV=1 | A0QEP1_MYCA1 | + | 0 | 0 | 0 | 0 | 0 |
| 1. UPF0234 protein OCU_44330 OS=Mycobacterium intracellulare (strain ATCC 13950 / DSM 43223 / JCM 6384 / NCTC 13025 / 3600) GN=OCU_44330 PE=3 SV=1 | H8ISK7_MYCIA | + | 0 | 0 | 0 | 0 | 0 |
| 1. Uncharacterized protein OS=Mycobacterium avium subsp. hominissuis TH135 GN=MAH_3546 PE=4 SV=1 | T2GWX9_MYCAV | + | 0 | 0 | 0 | 0 | 0 |
| 1. Amino acid ABC transporter OS=Mycobacterium fortuitum subsp. fortuitum DSM 46621 GN=MFORT_14757 PE=4 SV=1 | K0V755_MYCFO | 0 | 0 | + | 0 | + | + |
| 1. Uncharacterized protein OS=Mycobacterium intracellulare (strain ATCC 13950 / DSM 43223 / JCM 6384 / NCTC 13025 / 3600) GN=OCU_01440 PE=4 SV=1 | H8IJI5_MYCIA | + | 0 | 0 | 0 | 0 | 0 |
| 1. Uncharacterized protein OS=Mycobacterium avium subsp. hominissuis TH135 GN=MAH_4287 PE=3 SV=1 | T2GZ63_MYCAV | + | 0 | 0 | 0 | 0 | 0 |
| 1. Uncharacterized protein OS=Mycobacterium bovis (strain BCG / Pasteur 1173P2) GN=BCG_2580 PE=4 SV=1 | A1KLQ5_MYCBP | 0 | + | 0 | 0 | 0 | 0 |
| 1. Uncharacterized protein OS=Mycobacterium fortuitum subsp. fortuitum DSM 46621 GN=MFORT_29369 PE=4 SV=1 | K0V456_MYCFO | 0 | 0 | + | 0 | + | + |
| 1. Uncharacterized protein OS=Mycobacterium fortuitum subsp. fortuitum DSM 46621 GN=MFORT_05984 PE=4 SV=1 | K0V812_MYCFO | 0 | 0 | + |  | + | + |
| 1. Uncharacterized protein OS=Mycobacterium fortuitum subsp. fortuitum DSM 46621 GN=MFORT_11131 PE=4 SV=1 | K0VRZ3_MYCFO | 0 | 0 | + |  | + | + |
| 1. Cutinase OS=Mycobacterium fortuitum subsp. fortuitum DSM 46621 GN=MFORT_23055 PE=4 SV=1 | K0UR15_MYCFO | 0 | 0 | + |  | 0 | + |
| 1. Heparin-binding hemagglutinin OS=Mycobacterium kansasii ATCC 12478 GN=MKAN_18280 PE=4 SV=1 | U5WVQ5_MYCKA | 0 | 0 | 0 | + | 0 | 0 |
| 1. Peptide synthetase OS=Mycobacterium gastri 'Wayne' GN=MGAST_11190 PE=4 SV=1 | W4HYG5_MYCGS | 0 | 0 | 0 | + | 0 | 0 |
| 1. Nucleoid-associated protein MKAN_13420 OS=Mycobacterium kansasii ATCC 12478 GN=MKAN_13420 PE=3 SV=1 | U5WQE3_MYCKA | 0 | 0 | 0 | + | 0 | 0 |
| 1. Secretion protein EspL OS=Mycobacterium kansasii ATCC 12478 GN=MKAN_14565 PE=3 SV=1 | U5WTG7_MYCKA | 0 | 0 | 0 | + | 0 | 0 |
| 1. Thioredoxin OS=Mycobacterium kansasii ATCC 12478 GN=MKAN_14760 PE=3 SV=1 | U5WQD9_MYCKA | 0 | 0 | 0 | + | 0 | 0 |
| 1. ATP-dependent Clp protease proteolytic subunit OS=Mycobacterium kansasii ATCC 12478 GN=clpP PE=3 SV=1 | U5WPE7_MYCKA | 0 | 0 | 0 | + | 0 | 0 |
| 1. Uncharacterized protein OS=Mycobacterium kansasii ATCC 12478 GN=MKAN_10620 PE=4 SV=1 | U5WS21_MYCKA | 0 | 0 | 0 | + | 0 | 0 |
| 1. Uncharacterized protein OS=Mycobacterium kansasii ATCC 12478 GN=MKAN_13700 PE=4 SV=1 | U5WPW7_MYCKA | 0 | 0 | 0 | + | 0 | 0 |
| 1. Acyl-ACP desaturase OS=Mycobacterium kansasii ATCC 12478 GN=MKAN_08055 PE=4 SV=1 | U5WLZ6_MYCKA | 0 | 0 | 0 | + | 0 | 0 |
| 1. Uncharacterized protein OS=Mycobacterium kansasii ATCC 12478 GN=MKAN_22600 PE=4 SV=1 | U5WXL3_MYCKA | 0 | 0 | 0 | + | 0 | 0 |
| 1. Uncharacterized protein OS=Mycobacterium paratuberculosis (strain ATCC BAA-968 / K-10) GN=MAP_0298 PE=4 SV=1 | Q744P3_MYCPA | 0 | 0 | 0 | + | 0 | 0 |
| 1. Uncharacterized protein OS=Mycobacterium gastri 'Wayne' GN=MGAST_04715 PE=4 SV=1 | W4I3H2_MYCGS | 0 | 0 | 0 | + | 0 | 0 |
| 1. Uncharacterized protein OS=Mycobacterium xenopi RIVM700367 GN=MXEN_19184 PE=4 SV=1 | I0RG43_MYCXE | 0 | 0 | 0 | + | 0 | 0 |
| 1. Porin MspB OS=Mycobacterium smegmatis (strain ATCC 700084 / mc(2)155) GN=mspB PE=1 SV=1 | MSPB_MYCS2 | 0 | 0 | 0 | 0 | + | + |
| 1. Glycosyl hydrolase family protein OS=Mycobacterium fortuitum subsp. fortuitum DSM 46621 GN=MFORT_18233 PE=4 SV=1 | K0UYS3_MYCFO | 0 | 0 | 0 | 0 | + | + |
| 1. Cutinase OS=Mycobacterium fortuitum subsp. fortuitum DSM 46621 GN=MFORT_05563 PE=4 SV=1 | K0VV66_MYCFO | 0 | 0 | 0 | 0 | + | 0 |
| 1. Electron transfer flavoprotein subunit alpha OS=Mycobacterium fortuitum subsp. fortuitum DSM 46621 GN=MFORT_03271 PE=4 SV=1 | K0V9J2_MYCFO | 0 | 0 | 0 | 0 | + | + |
| 1. Uncharacterized protein OS=Mycobacterium fortuitum subsp. fortuitum DSM 46621 GN=MFORT_27004 PE=4 SV=1 | K0UWS1_MYCFO | 0 | 0 | 0 | 0 | + | + |
| 1. ErfK/YbiS/YcfS/YnhG family protein OS=Mycobacterium fortuitum subsp. fortuitum DSM 46621 GN=MFORT_24012 PE=4 SV=1 | K0UNJ1_MYCFO | 0 | 0 | 0 | 0 | + | + |
| 1. Uncharacterized protein OS=Mycobacterium fortuitum subsp. fortuitum DSM 46621 GN=MFORT_19097 PE=4 SV=1 | K0UWS9_MYCFO | 0 | 0 | 0 | 0 | + | + |
| 1. D-alanyl-D-alanine dipeptidase OS=Mycobacterium fortuitum subsp. fortuitum DSM 46621 GN=MFORT_00904 PE=4 SV=1 | K0VAY5_MYCFO | 0 | 0 | 0 | 0 | + | + |
| 1. Transcription elongation factor GreA OS=Mycobacterium fortuitum subsp. fortuitum DSM 46621 GN=greA PE=3 SV=1 | K0VBA2_MYCFO | 0 | 0 | 0 | 0 | + | 0 |
| 1. Nucleoid-associated protein MFORT_10794 OS=Mycobacterium fortuitum subsp. fortuitum DSM 46621 GN=MFORT_10794 PE=3 SV=1 | K0VH29_MYCFO | 0 | 0 | 0 | 0 | + | 0 |
| 1. Uncharacterized protein OS=Mycobacterium fortuitum subsp. fortuitum DSM 46621 GN=MFORT_16679 PE=4 SV=1 | K0V0I2_MYCFO | 0 | 0 | 0 | 0 | + | + |
| 1. ErfK/YbiS/YcfS/YnhG family protein OS=Mycobacterium fortuitum subsp. fortuitum DSM 46621 GN=MFORT_17748 PE=4 SV=1 | K0UYU7_MYCFO | 0 | 0 | 0 | 0 | + |  |
| 1. Glyceraldehyde-3-phosphate dehydrogenase OS=Mycobacterium fortuitum subsp. fortuitum DSM 46621 GN=MFORT_05213 PE=3 SV=1 | K0VCJ6_MYCFO | 0 | 0 | 0 | 0 | + | + |
| 1. Uncharacterized protein OS=Mycobacterium fortuitum subsp. fortuitum DSM 46621 GN=MFORT_10259 PE=4 SV=1 | K0V5L1_MYCFO | 0 | 0 | 0 | 0 | + | 0 |
| 1. Uncharacterized protein OS=Mycobacterium fortuitum subsp. fortuitum DSM 46621 GN=MFORT_13775 PE=4 SV=1 | K0VQJ9_MYCFO | 0 | 0 | 0 | 0 | + | 0 |
| 1. Uncharacterized protein OS=Mycobacterium fortuitum subsp. fortuitum DSM 46621 GN=MFORT_12646 PE=4 SV=1 | K0V4G8_MYCFO | 0 | 0 | 0 | 0 | + | 0 |
| 1. Lipoprotein OS=Mycobacterium fortuitum subsp. fortuitum DSM 46621 GN=MFORT_19504 PE=4 SV=1 | K0UWA4_MYCFO | 0 | 0 | 0 | 0 | + | 0 |
| 1. NLP/P60 protein OS=Mycobacterium fortuitum subsp. fortuitum DSM 46621 GN=MFORT_20700 PE=4 SV=1 | K0UT56_MYCFO | 0 | 0 | 0 | 0 | + | 0 |
| 1. PPE family protein OS=Mycobacterium fortuitum subsp. fortuitum DSM 46621 GN=MFORT_10334 PE=4 SV=1 | K0V5M8_MYCFO | 0 | 0 | 0 | 0 | + | 0 |
| 1. Putative uncharacterized protein OS=Mycobacterium rhodesiae JS60 GN=MycrhDRAFT_5555 PE=4 SV=1 | G4I642_MYCRH | 0 | 0 | 0 | 0 | + | 0 |
| 1. N-acetylmuramoyl-L-alanine amidase OS=Mycobacterium fortuitum subsp. fortuitum DSM 46621 GN=MFORT_10789 PE=4 SV=1 | K0V4H1_MYCFO | 0 | 0 | 0 | 0 | 0 | + |
| 1. Uncharacterized protein OS=Mycobacterium fortuitum subsp. fortuitum DSM 46621 GN=MFORT_12231 PE=4 SV=1 | K0VRK2_MYCFO | 0 | 0 | 0 | 0 | 0 | + |
| 1. Uncharacterized protein OS=Mycobacterium fortuitum subsp. fortuitum DSM 46621 GN=MFORT_24522 PE=4 SV=1 | K0VBI1_MYCFO | 0 | 0 | 0 | 0 | 0 | + |
| 1. 3-oxoacyl-(Acyl carrier protein) synthase II OS=Mycobacterium fortuitum subsp. fortuitum DSM 46621 GN=MFORT_12861 PE=3 SV=1 | K0V8A4_MYCFO | 0 | 0 | 0 | 0 | 0 | + |
| 1. Steroid delta-isomerase OS=Mycobacterium fortuitum subsp. fortuitum DSM 46621 GN=MFORT_00959 PE=4 SV=1 | K0VAR6_MYCFO | 0 | 0 | 0 | 0 | 0 | + |
| 1. **DNA-directed RNA polymerase subunit alpha OS=Mycobacterium kansasii ATCC 12478 GN=rpoA PE=3 SV=1** | U5WSX2\|U5WSX2_MYCKA | + | + | + | + | + | + |
| 1. **ATP-dependent Clp protease proteolytic subunit OS=Mycobacterium bovis (strain BCG / Pasteur 1173P2) GN=clpP2 PE=3 SV=1** | A1KLF6\|A1KLF6_MYCBP | + | + | 0 |  | + | 0 |
| 1. **Universal stress protein OS=Mycobacterium kansasii ATCC 12478 GN=MKAN_28080 PE=4 SV=1** | U5WXR9\|U5WXR9_MYCKA | 0 | + | 0 | + | 0 | 0 |
| 1. Ribonucleoside-diphosphate reductase subunit beta OS=Mycobacterium intracellulare (strain ATCC 13950 / DSM 43223 / JCM 6384 / NCTC 13025 / 3600) GN=OCU_37470 PE=3 SV=1 | H8IK89\|H8IK89_MYCIA | + | 0 | 0 | 0 | + | + |
| 1. Fructose-bisphosphate aldolase class-I OS=Mycobacterium avium (strain 104) GN=MAV_5271 PE=4 SV=1 | A0QN95\|A0QN95_MYCA1 | + | 0 | 0 | 0 | 0 | + |
| 1. Uncharacterized protein OS=Mycobacterium kansasii ATCC 12478 GN=MKAN_00535 PE=4 SV=1 | U5WIH7\|U5WIH7_MYCKA | + | 0 | 0 | + | 0 | 0 |
| 1. ATP synthase subunit beta OS=Mycobacterium avium (strain 104) GN=atpD PE=3 SV=1 | A0QCX8\|ATPB_MYCA1 | + | + |  | + |  | + |

+, protein detected in the PPD; 0, the protein was not detected in the PPD.
